# Synthesis and Optical Characterization of Hydrazone-Substituted Push–Pull-Type NLOphores

**DOI:** 10.1021/acs.joc.4c01328

**Published:** 2024-09-10

**Authors:** Kübra Erden, Dilek Soyler, Alberto Barsella, Onur Şahin, Saniye Soylemez, Cagatay Dengiz

**Affiliations:** † Department of Chemistry, Middle East Technical University, 06800 Ankara, Turkey; ‡ Department of Biomedical Engineering, Necmettin Erbakan University, 42090 Konya, Turkey; § Science and Technology Research and Application Center (BİTAM), Necmettin Erbakan University, 42090 Konya, Turkey; ∥ Département d’Optique Ultra-Rapide et Nanophotonique, IPCMS-CNRS, 23 Rue du Loess, BP 43, 67034 Strasbourg, Cedex 2, France; ⊥ Department of Occupational Health & Safety, Faculty of Health Sciences, Sinop University, Sinop 57000, Turkey

## Abstract

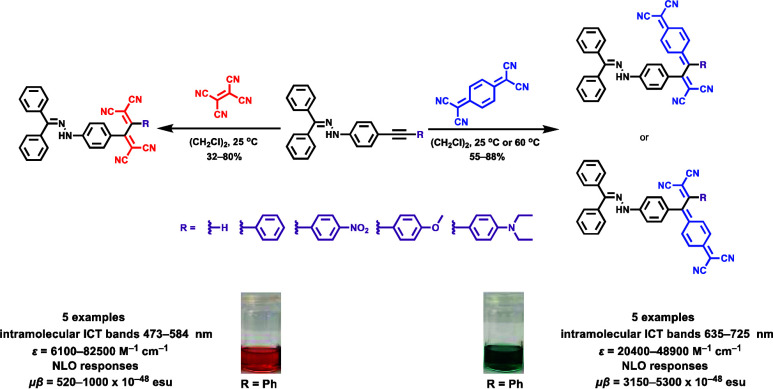

Two distinct families of NLOphores featuring hydrazone donors were synthesized using click-type [2 + 2] cycloaddition retroelectrocyclizations (CA-RE). Despite the limitations in the substrate scope, it was shown for the first time that hydrazone-activated alkynes could undergo reactions with TCNE/TCNQ. The electrochemical, photophysical, and second-order nonlinear optical (NLO) characteristics of the chromophores were analyzed utilizing experimental and computational approaches. Chromophores **17**–**21** and **23**–**27** exhibited two reduction waves, along with one oxidation wave that can be attributed to the hydrazone moiety. All chromophores exhibit charge-transfer bands extending from the visible to the near-infrared region. The λ_max_ of hydrazone-based chromophores falls within the range of 473 to 725 nm. Additionally, all chromophores exhibited positive solvatochromism. Computational studies have been performed to elucidate the origin of the low-energy absorption bands. Parameters such as dipole moment, band gaps, electronegativity, global chemical hardness/softness, average polarizability, and first hyperpolarizability were calculated to obtain information about NLO properties of the target structures. The thermal stabilities of the NLOphores were assessed through TGA. Experimental NLO measurements were conducted using the electric field-induced second harmonic generation (EFISHG) technique. The studied structures demonstrated NLO responses, with μβ values between 520 × 10^–48^ esu and 5300 × 10^–48^ esu.

## Introduction

The demand is increasing for the design and synthesis of new materials with tailor-made properties to address current global challenges in areas such as energy, health, and sustainability.^1^ In order to prevent the emergence of additional issues while seeking solutions to these global problems, environmentally friendly and sustainable synthetic strategies are required to be developed.^2^ In 2001, Sharpless, along with Kolb and Finn, introduced the concept of “click” chemistry for the first time.^3^ Click chemistry was emerged from the need for a robust and practical approach to chemical synthesis that could be used in a variety of scientific fields. For a reaction to fall within the scope of “click chemistry,″ certain conditions need to be met. These include readily available starting materials, easily extractable solvents, simple product purification, high yields in a short time frame, and ideally minimal or no byproducts.^3−5^ These strict criteria significantly limit the scope of chemical transformations that can be classified within this category. Besides azide–alkyne Huisgen cycloadditions, Diels–Alder reactions, and alkene hydrothiolations also fall within the scope of click chemistry, constituting some of the most recognized reactions associated with this definition.^6−8^ Each of these three reactions encounters certain constraints, including the utilization of high-energy starting materials, the generation of unconjugated products, and issues related to high-temperature requirements. In efforts to widen the scope of these limited group of reactions, researchers are actively engaged in developing new, highly efficient reactions. In recent years, one of the most noteworthy advancements in this field is the exploration of [2 + 2] cycloaddition-retroelectrocyclization (CA-RE) reactions.^9,10^ The first example of this reaction was documented in 1981 by Bruce and colleagues.^11^ They observed the reaction between metal acetylides and tetracyanoethylene (TCNE), yielding 1,1,4,4-tetracyanobutadienes. In two independent studies published in 1999, [2 + 2] CA-RE was documented for the first time on metal-free organic substrates.^12,13^ In 2005, the [2 + 2] CA-RE reactions of dimethylaniline-substituted alkynes, achieving yields of 97–100% by Diederich and co-workers, attracted significant attention within the research community, leading to a significant surge in the number of subsequent studies.^9,10,14^ The interest in these reactions arises not only from their high yields but also from the fact that the products are donor–acceptor-type conjugated molecules with a nonplanar structure.^10^ Taking advantage of conjugated nature of the resulting structures, the [2 + 2] CA-RE have recently been used to obtain structurally demanding systems such as dendrimers,^15^ active layer materials for solar cells,^16−19^ nonlinear optical (NLO) materials,^20−22^ ion-sensors,^23^ polymers,^10^ fluorophores,^24−33^ Aviram-Ratner-type molecular rectifiers.^34^ Despite providing significant results, [2 + 2] CA-RE reactions have not seen widespread application, primarily due to constraints such as a limited substrate scope and the inability to perform postreaction modifications. The current research on [2 + 2] CA-RE can be categorized into three primary areas: the exploration of new donor^20,21,28,35−38^ and acceptor groups,^39−41^ modifications applied after the [2 + 2] CA-RE,^42−44^ and utilizing the [2 + 2] CA-RE in synthesizing target products with desired optical properties.^10^ When the history of [2 + 2] CA-RE reactions for about half a century is examined, the donor groups used in these reactions can be listed as metal acetylides,^11^ thiophene,^12^ dialkylanilines,^14^ anisole,^45^ azulene,^46^ ferrocene,^47^ tetrathiafulvalene,^47^ cyclopenta[*b*]furan-2-one,^36^ porphyrin,^48^ ynamides,^38^ carbazole,^49^ phenothiazine,^50^ urea,^28^ triazene,^20^*N*-alkylindole,^37^ and γ-pyranylidene.^21^ The primary olefins typically employed in such reactions alongside donor-substituted alkynes are TCNE and tetracyanoquinodimethane (TCNQ).^10^ However, the options for donor groups suitable for reactions involving both TCNE and comparatively larger TCNQ are quite limited. Hence, there is a significant demand to identify donor-substituted alkynes capable of reacting with both alkenes efficiently. As part of our ongoing research, we have recently demonstrated the effectiveness *N-*alkylindoles as new donor group in [2 + 2] CA-RE reactions.^37^ Herein, we have shown that hydrazone-substituted alkynes, a previously unreported substrate for [2 + 2] CA-RE, react with both TCNE and TCNQ, yielding almost quantitative yields. This introduces hydrazones as new donor groups to the existing repertoire for such reactions documented in the literature.

## Results and Discussion

### Synthesis and Characterizations

**Scheme 1:**
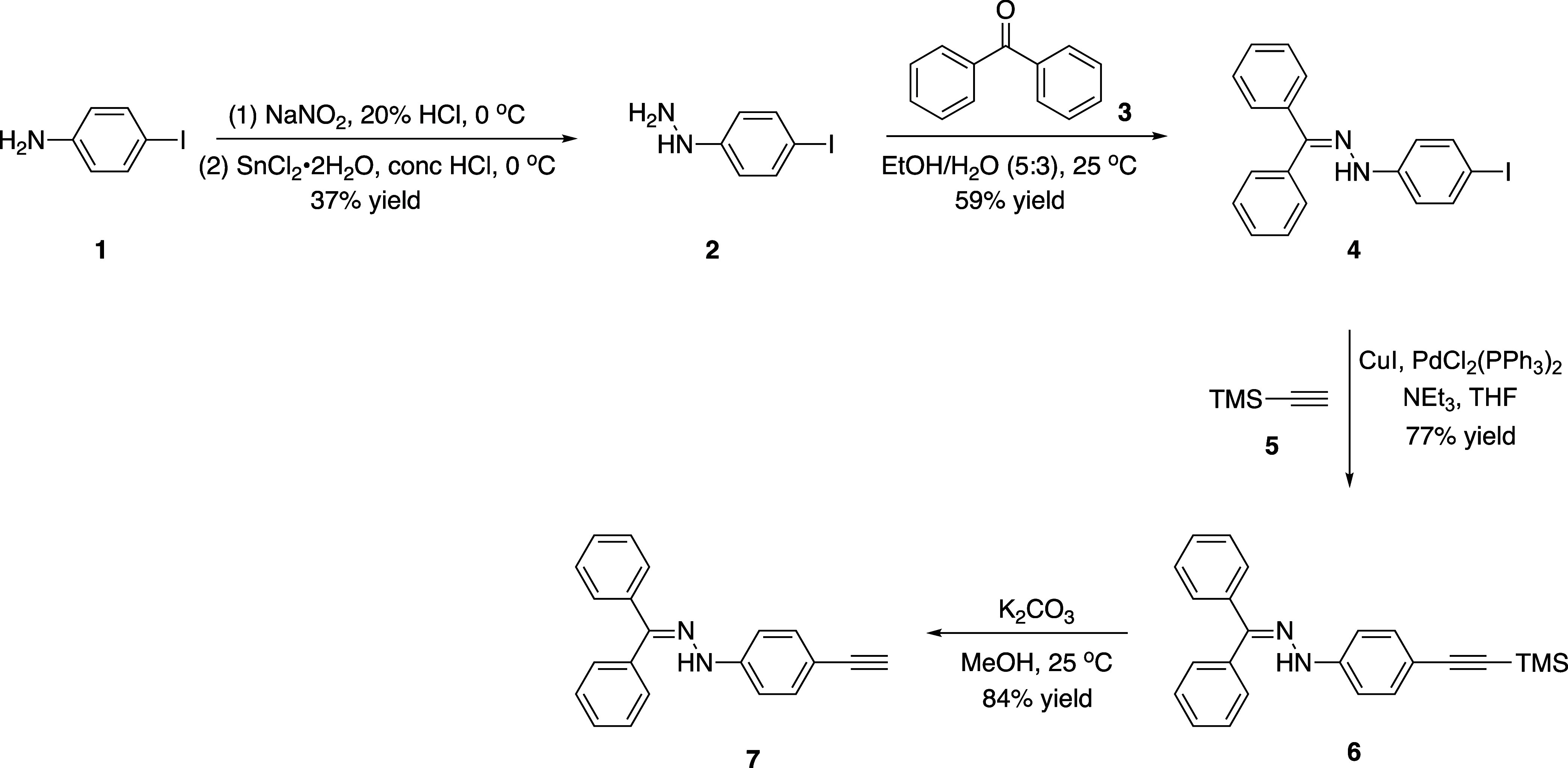
Synthesis of Hydrazone-Substituted Terminal Alkyne **7**

The synthetic part of the study was carried out in two steps: synthesis of donor-substituted alkynes and [2 + 2] CA-RE reactions of these alkynes with TCNE and TCNQ. Aryl iodide **4**, the key compound of the study, was synthesized through a two-step process (Scheme 1). Initially, commercially available 4-iodoaniline (**1**) was transformed into a phenylhydrazine derivative **2**.^51^ Subsequently, compound **4** was obtained by reacting compound **2** with benzophenone (**3**) under mild conditions. Having aryl iodide **4** in hand, the synthesis of hydrazone-substituted terminal alkyne **7** was carried out through Sonogashira cross-coupling with **4** and trimethylsilylacetylene (TMSA) (**5**), followed by a TMS deprotection step facilitated by K_2_CO_3_.

**Scheme 2:**
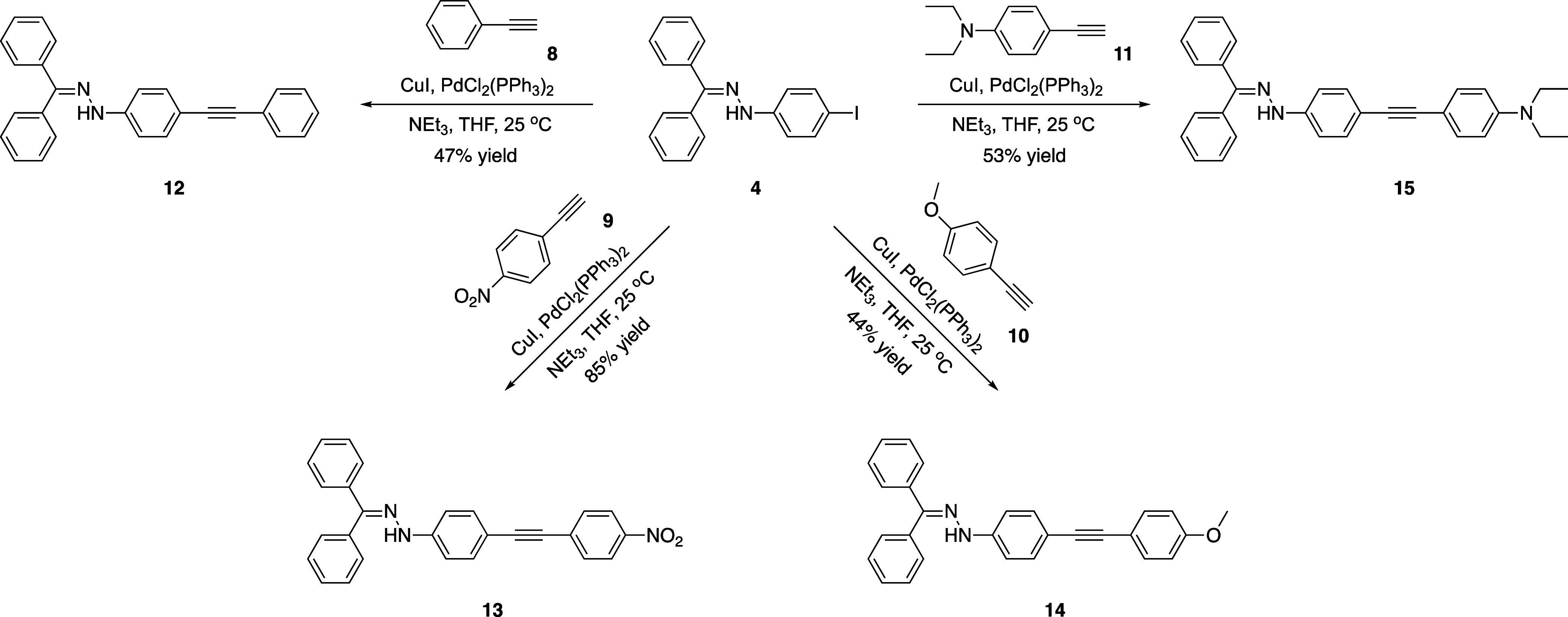
Synthesis of Disubstituted Alkynes **12**–**15** via Sonogashira Cross-Coupling Reactions

Scheme 2 outlines the synthesis of various disubstituted alkynes synthesized in the scope of the study. Apart from commercially available phenylacetylene (**8**), terminal alkynes **9**–**11** required for the synthesis of disubstituted alkynes were prepared according to previously published methods.^52−54^ Sonogashira cross-coupling reactions were conducted at room temperature using iodoarene **4** and terminal alkynes **8**, **9**, **10**, and **11**, resulting in the formation of the desired disubstituted alkynes **12**, **13**, **14**, and **15** with yields of 47, 85, 44, and 53%, respectively.

**Scheme 3:**
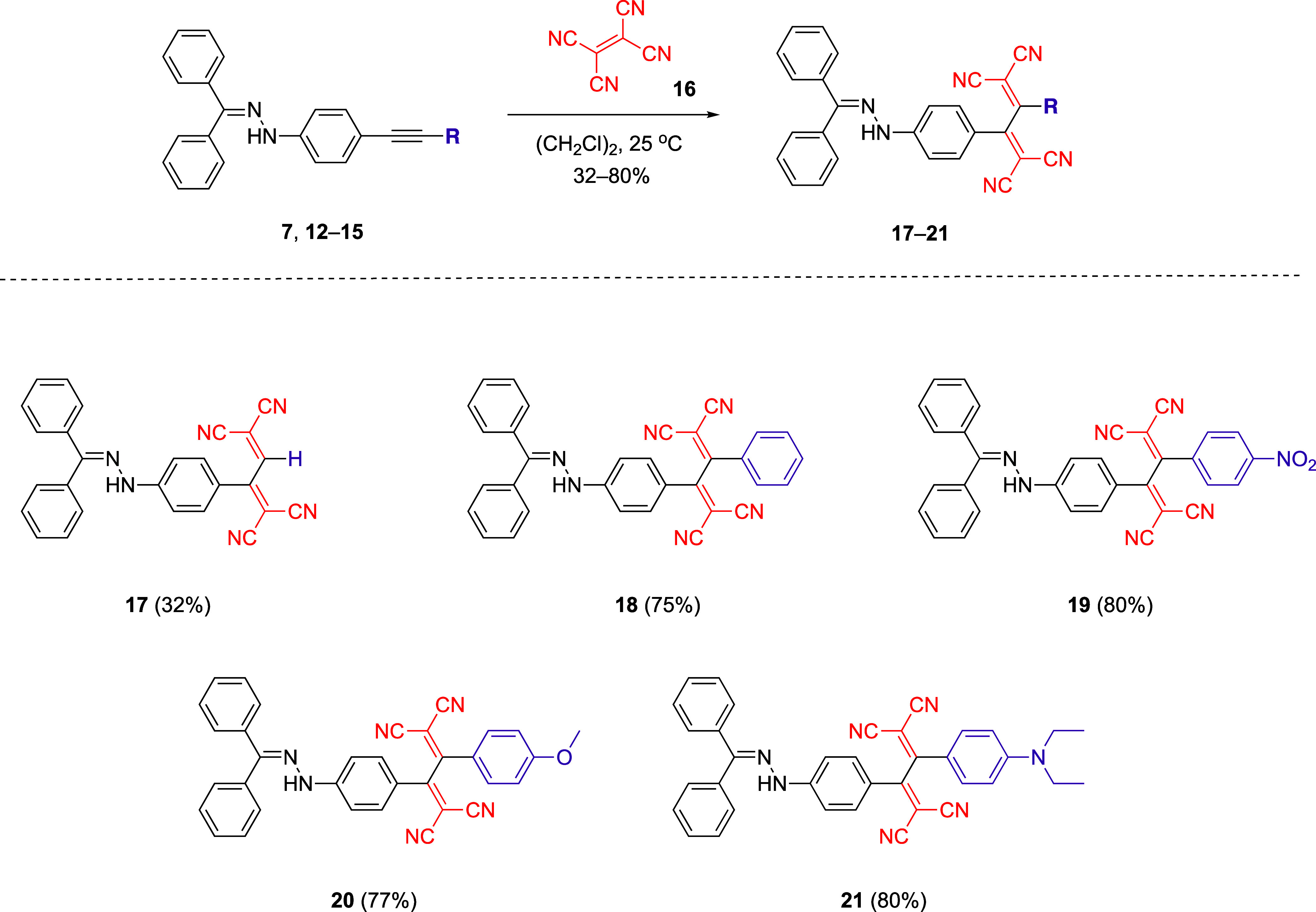
CA-RE Reactions between Hydrazone-Substituted Alkynes **7**, **12**–**15** and TCNE

Following the successful synthesis of alkynes incorporating hydrazone and diverse functional groups (such as donor, acceptor, and aromatic moieties), we initiated our [2 + 2] CA-RE experiments by employing TCNE as the electron acceptor (Scheme 3). TLC analysis revealed quantitative reaction of all substrates with TCNE; however, moderate yields were obtained due to undesired hydrolysis and decomposition reactions encountered during purification via column chromatography (SiO_2_). To assess the impact of hydrazone donor groups in activating alkynes for [2 + 2] CA-RE reactions, the initial experiment was conducted using substrate **7** and TCNE **16**. Although the isolated yield was as low as 32%, our findings confirm our hypothesis regarding the donor capabilities of hydrazones in [2 + 2] CA-RE. Substrates **12** and **13**, bearing phenyl and para-nitrophenyl substituents, respectively, were found to react with TCNE in higher yields (75 and 80% respectively) compared to substrate **7**. The reactions involving substrates **14** and **15** substituted with electron-donating groups indicate that there is no substantial difference in the yields of chromophores obtained when utilizing substrates with either electron-withdrawing or electron-donating groups.

**Scheme 4:**
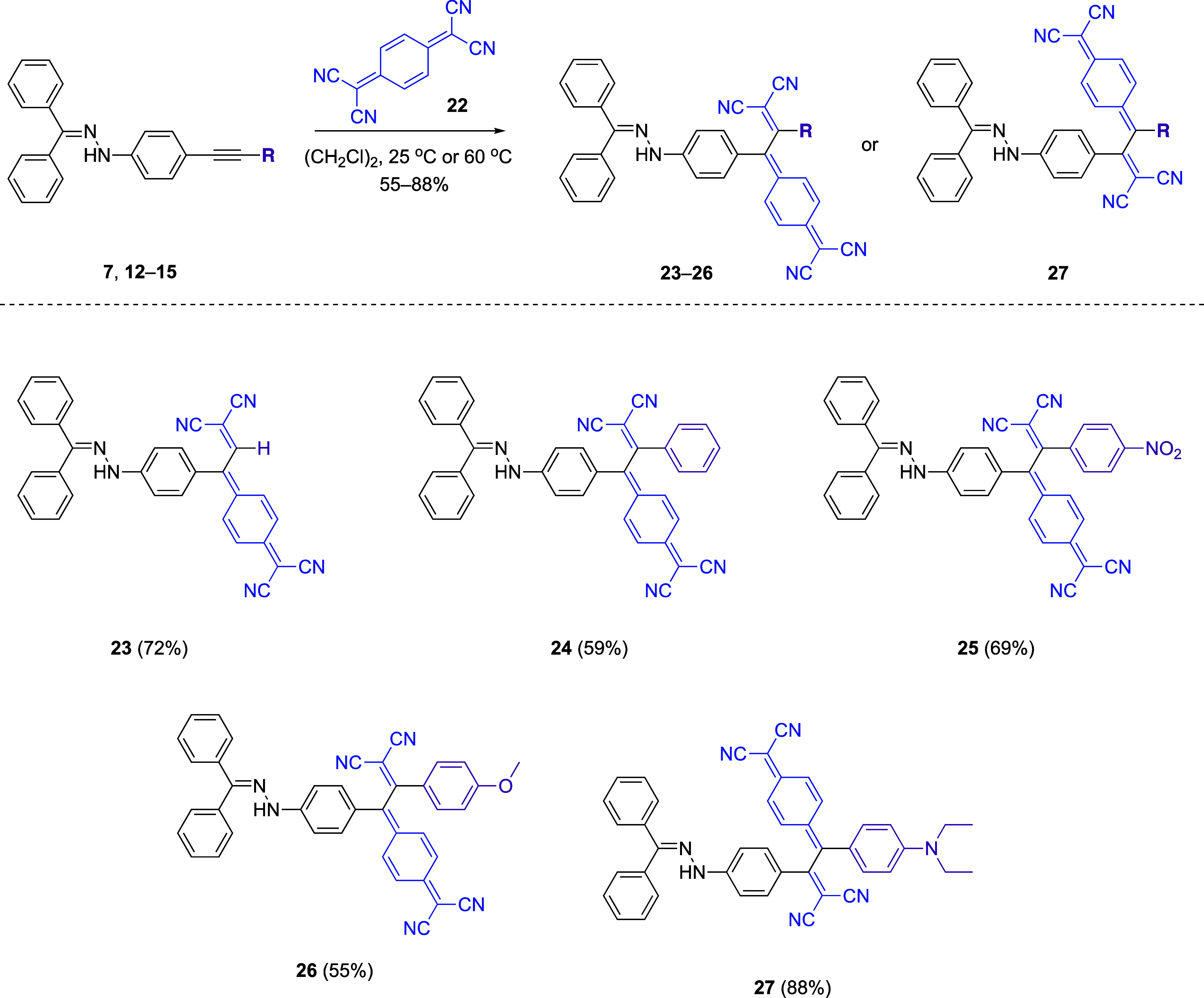
CA-RE Reactions between Hydrazone-Substituted Alkynes **7**, **12**–**15** and TCNQ

As previously mentioned, the range of donor-substituted substrates capable of reacting with TCNQ is significantly more restricted compared to those capable of reacting with TCNE.^37^ The reaction between terminal alkyne **7** and TCNQ **22** under ambient conditions demonstrated that hydrazone groups also effectively activate alkynes for [2 + 2] CA-RE with TCNQ, yielding the desired product **23**, which was isolated in a 72% yield (Scheme 4). Following this promising outcome, the subsequent reactions involving disubstituted alkynes bearing phenyl **12**, nitrophenyl **13**, and methoxyphenyl **14** groups with TCNQ **22** could not be carried at room temperature. Instead, the desired products **24**, **25**, and **26** were obtained in yields of 59, 69, and 55% respectively, from reactions performed at 60 °C in an oil bath. These results indicate that the reaction of TCNQ, a bulky electron acceptor, takes place at higher temperatures for steric reasons, while methoxybenzene, a mild donor group, cannot sufficiently activate the alkyne to react at room temperature.

**Figure 1:**
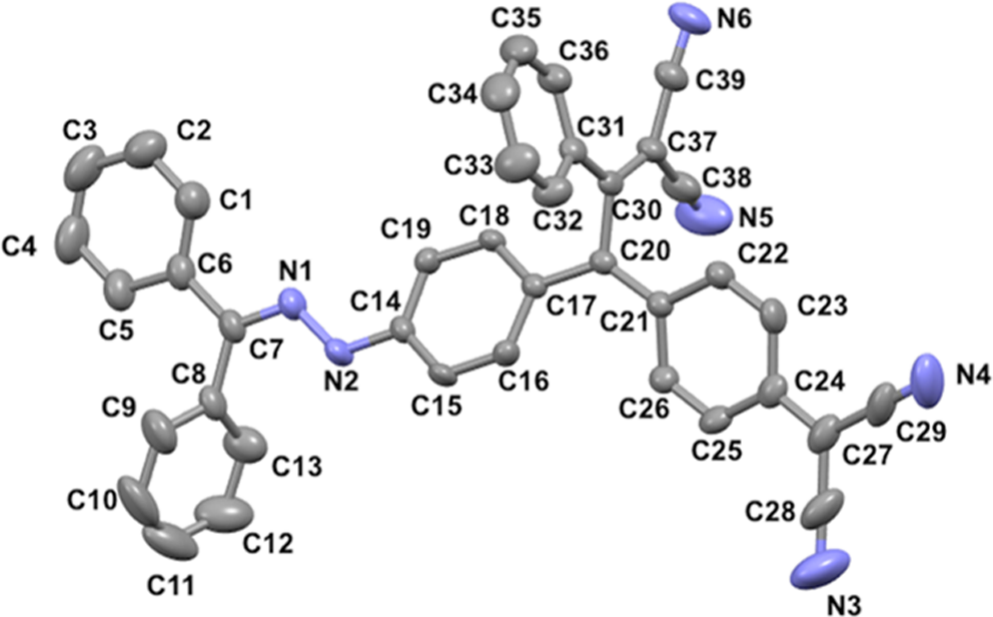
ORTEP representation of **24** with vibrational ellipsoids shown at the 50% probability level, arbitrary numbering. H atoms and solvent molecules are omitted for clarity.

Single crystals of chromophore **24** suitable for X-ray analysis were obtained through slow evaporation from a solution of DCM/*n*-hexane at a temperature of 25 °C. The structure of compound **24** (CCDC 2357634) was confirmed through X-ray analysis, as depicted in Figure 1. The reaction of TCNQ with alkyne **15**, which was substituted with a diethylaniline donor group known to be much stronger than methoxybenzene, proceeded at room temperature as anticipated. Interestingly, two-dimensional (2D) HMBC (heteronuclear multiple quantum coherence) NMR analysis confirmed that the regioselectivity of the reaction is completely changed due to the fact that the diethylaniline group is also a stronger donor group than hydrazone (Figure S37 in the Supporting Information).

### UV/vis Spectroscopy

**Figure 2:**
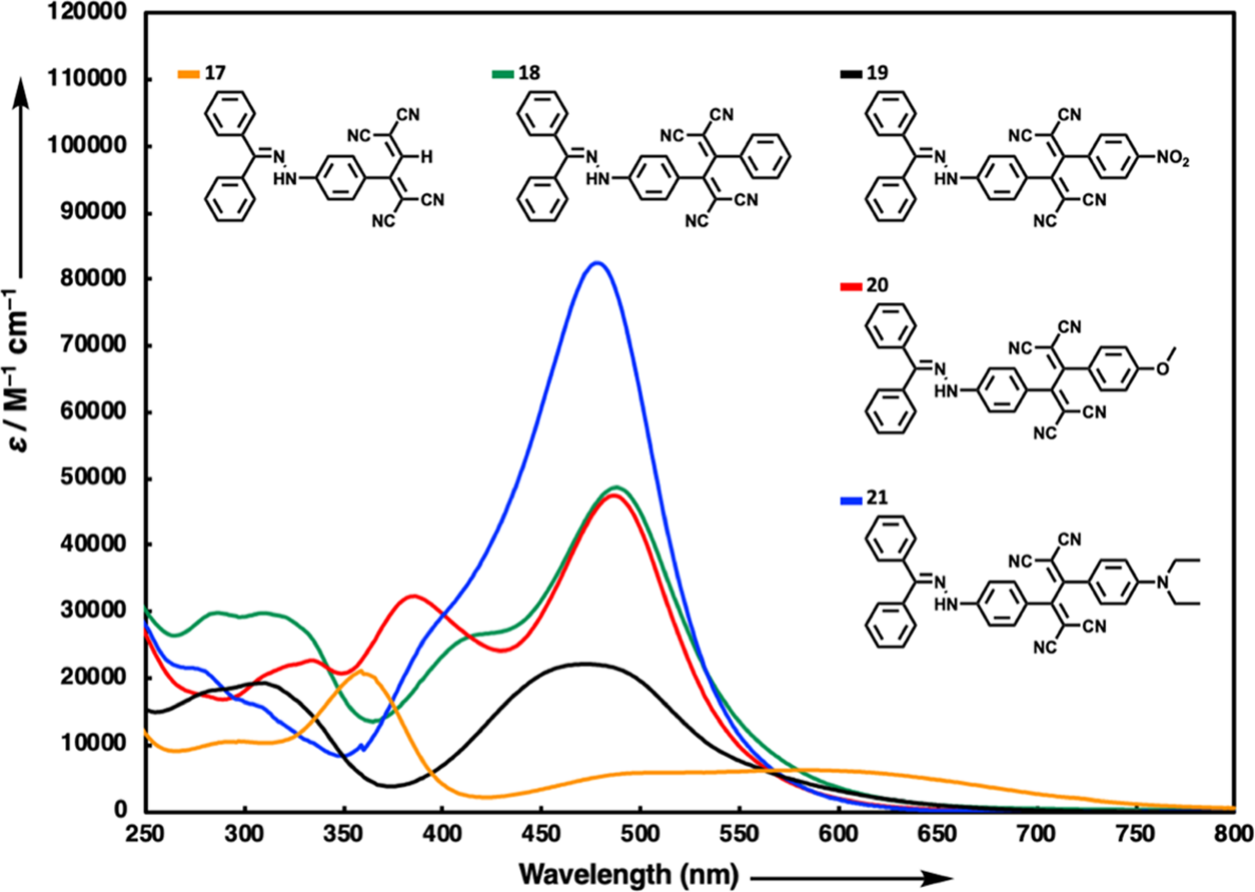
UV/vis spectra of NLOphores **17**–**21** in CH_2_Cl_2_ (2 × 10^–5^ M) at 25 °C.

The hydrazone-substituted chromophores obtained by both TCNE and TCNQ reactions are highly colored (refer to Figures S55 and S56 in the Supporting Information for images of chromophore solutions) and absorb light in a broad spectrum due to their intense intramolecular charge-transfer (ICT) bands. Figure 2 illustrates the absorption spectra of the initial chromophore group, 1,1,4,4-tetracyanobuta-1,3-dienes (TCBDs) **17**–**21**. The lowest energy ICT bands within this series exhibit peak wavelengths (λ_max_) ranging from 473 to 584 nm and molar extinction coefficients ranging from 6100 to 82,500 M^–1^ cm^–1^. The summarized characteristics of the ICT bands associated with each compound are as follows: λ_max_ = 584 nm (6100 M^–1^ cm^–1^ for **17**), λ_max_ = 488 nm (48,700 M^–1^ cm^–1^ for **18**), λ_max_ = 473 nm (22,200 M^–1^ cm^–1^ for **19**), λ_max_ = 487 nm (47,500 M^–1^ cm^–1^ for **20**), λ_max_ = 478 nm (82,500 M^–1^ cm^–1^ for **21**). The unsubstituted structure **17** shows a very pronounced bathochromic shift compared to all other chromophores. Initially, we speculated that this observation might be attributed to steric factors. However, we later realized that the dihedral angle between the hydrazone-substituted benzene ring and the conjugated dicyanovinyl group is greater in the compound **17** (28.3°) compared to **18**–**21** (18.4–24.5°). Considering TCBD unit of the chromophores, it is noted that the dihedral angle of TCBD significantly increases with the substitution of H atom in **17** with aromatic groups (64.2° for **17**, 88.1–89.9° for **18**–**21**). This indicates that the second dicyanovinyl group in compound **17**, which is directly conjugated to the aromatic ring, also participates in charge transfer, thereby forming a stronger donor–acceptor (D–A) system. Additionally, the through-space interaction between the hydrazone donor and the dicyanovinyl acceptor may represent a weaker donor–acceptor interaction. This interaction should not be overlooked, as it might result in charge transfer bands at a longer wavelength. No significant difference was observed in the λ_max_ values of the ICT bands among compounds **18**–**21**. Among this series, the chromophore substituted with electron-withdrawing nitrobenzene group exhibited the lowest λ_max_ value at 473 nm. On the other hand, compounds substituted with phenyl and electron-donating groups such as methoxybenzene and diethylaniline did not display any significant trend in λ_max_ values. This could be attributed to the orthogonal arrangement of the substituted side groups within the molecular structure.

**Figure 3:**
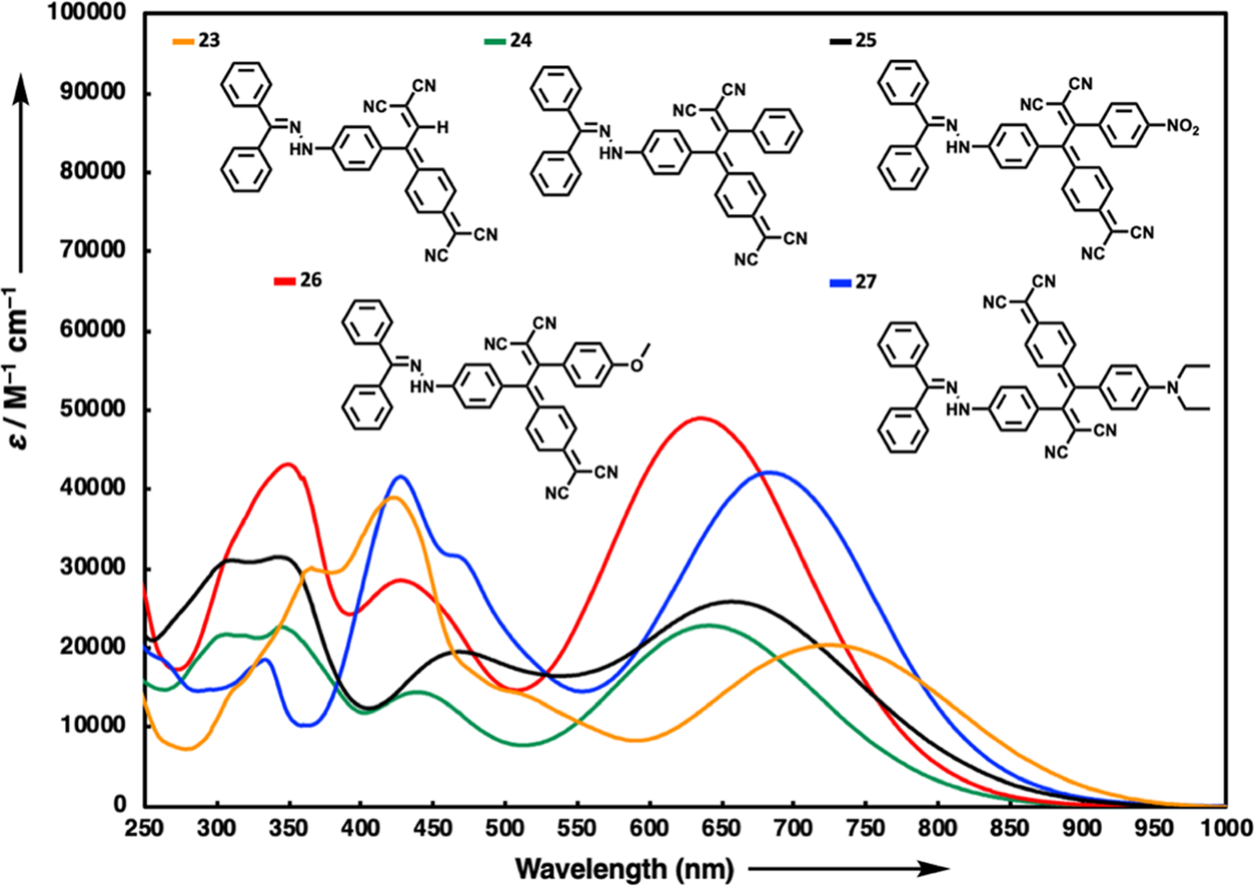
UV/vis spectra of NLOphores **23**–**27** in CH_2_Cl_2_ (2 × 10^–5^ M) at 25 °C.

Upon analyzing the UV/vis absorption spectra of chromophores **23**–**27** obtained with TCNQ as depicted in Figure 3, it becomes evident that their lowest energy CT bands are absorbed in the red region, positioning them closer to the near-infrared (NIR) compared to chromophores **17**–**21** obtained via TCNE reactions. The significant difference arises from the extended conjugation pathways present in the chromophores of the latter series. Additionally, the incorporation of a proaromatic 6-membered ring into the structure by TCNQ enhances the efficiency of charge transfer interactions. Below are the summarized characteristics of the lowest energy ICT bands for chromophores **23**–**27**: λ_max_ = 725 nm (20,400 M^–1^ cm^–1^ for **23**), λ_max_ = 641 nm (22,800 M^–1^ cm^–1^ for **24**), λ_max_ = 657 nm (25,800 M^–1^ cm^–1^ for **25**), λ_max_ = 635 nm (48,900 M^–1^ cm^–1^ for **26**), λ_max_ = 683 nm (42,200 M^–1^ cm^–1^ for **27**). Like compound **17**, the chromophore with the lowest energy ICT absorption band among the chromophores obtained with TCNQ was unsubstituted **23**. When comparing chromophores **24**, **25**, and **26** among themselves, it is observed that **26**, featuring the methoxybenzene side group as the electron donor, exhibits a hypsochromic shift compared to the phenyl-substituted chromophore **24**. Conversely, the presence of the electron-withdrawing nitrobenzene group in **25** significantly shifts the CT band bathochromically as expected.

**Figure 4:**
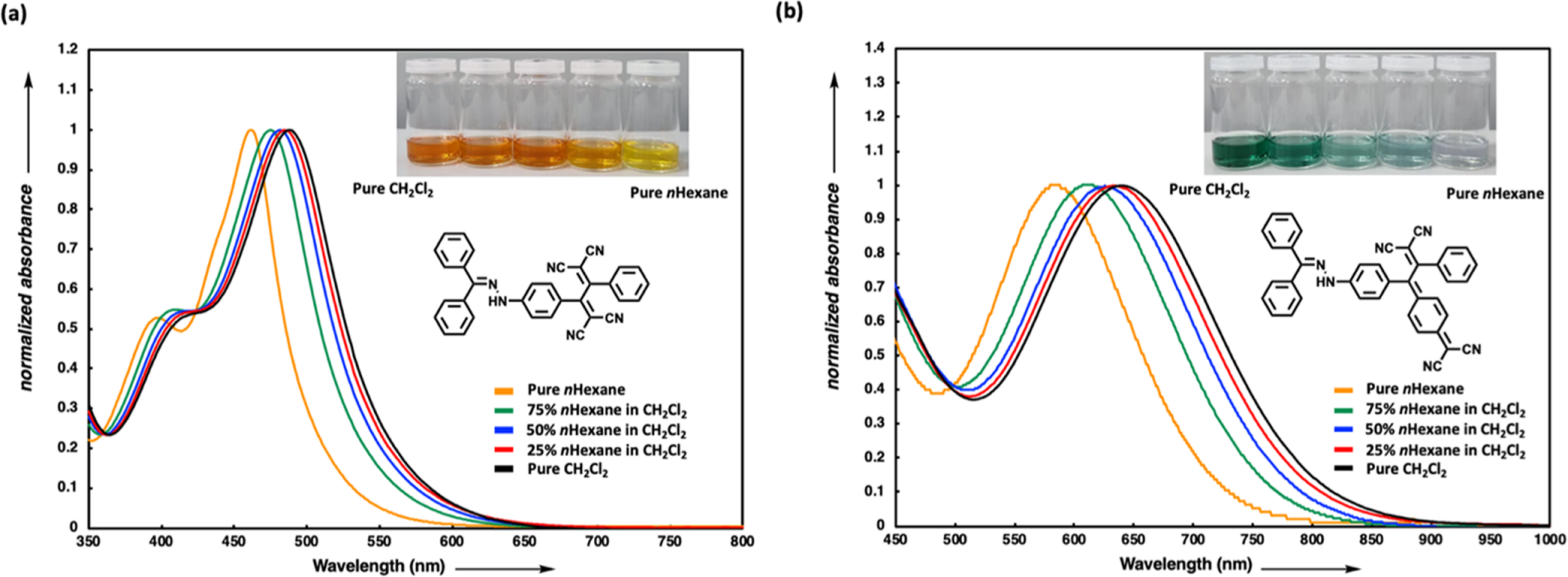
UV/vis spectra of the representative NLOphores (a) **18** and (b) **24** in CH_2_Cl_2_/*n*-hexane mixtures at 25 °C.

Solvatochromism, characterized by the changes in a molecule’s absorption spectrum across diverse solvents, serves as a valuable tool for elucidating ICT processes.^55^ The solvation of a molecule in various solvents has the potential to change its molecular geometry, subsequently impacting the electronic structure and influencing the likelihood of charge transfer occurring.^55^ Another reason for the observation of solvatochromism is that excited states are selectively stabilized by polar solvents relative to ground states.^56,57^Figure 4 shows the absorption spectra of compounds **18** and **24**, chosen as representatives from both groups, in DCM/*n-*hexane mixtures. In both cases, a positive solvatochromism is evident when moving from the nonpolar solvent *n-*hexane to the more polar dichloromethane (DCM), which confirms the ICT mechanism. In the initial series, a change in the solution color from yellow to dark orange was noted, overlapping with a shift in the ICT bands [462 nm (2.68 eV) to 488 nm (2.54 eV)] from a nonpolar solvent (*n-*hexane) to a polar solvent (DCM) (Figure 4a). Examination of the ICT absorption bands of chromophore **24** across different solvent mixtures reveals a particularly pronounced shift from 584 nm (2.12 eV) to 641 nm (1.93 eV). As a result of this shift, the color of the solutions changes from pale purple in pure *n-*hexane to dark green in pure DCM (Figure 4b). Additionally, NLOphores **17**–**21** and **23**–**27** demonstrated nonemissive behavior in DCM.

### Thermal Gravimetric Analysis (TGA)

**Figure 5:**
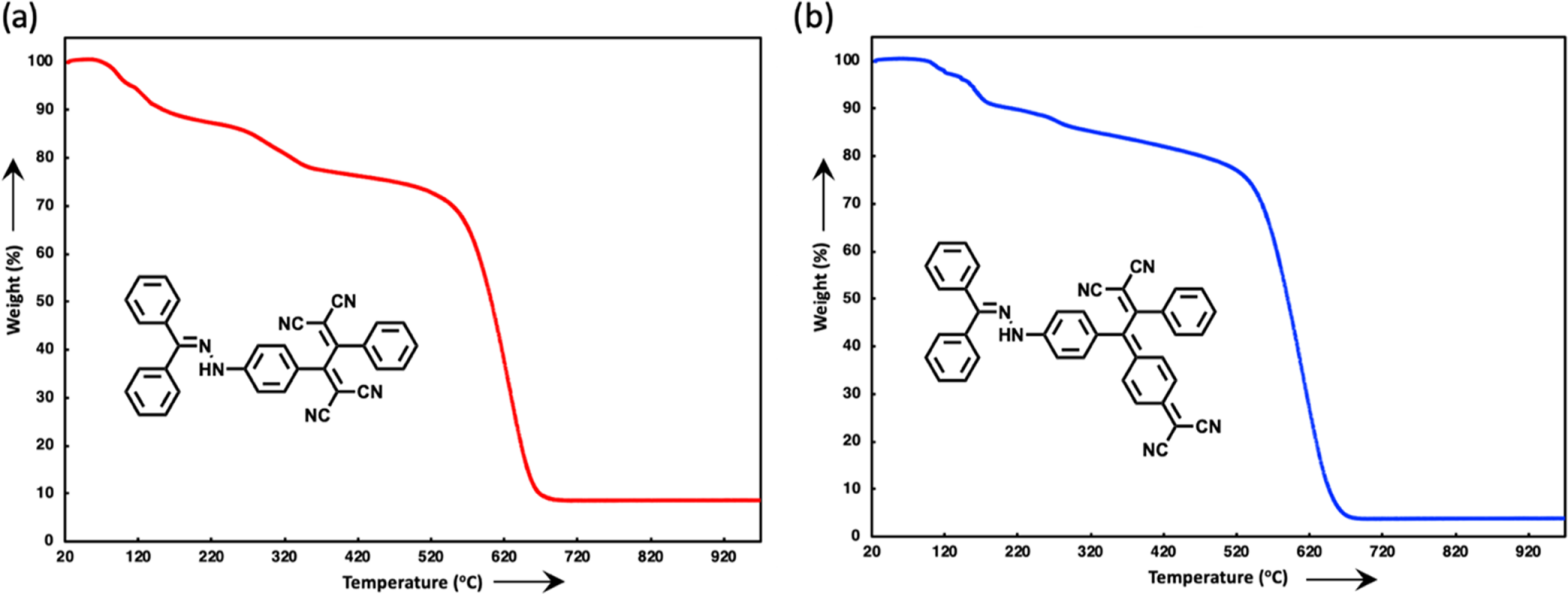
TGA curves for (a) **18** and (b) **24**.

Thermogravimetric analysis (TGA) is a routine technique employed to evaluate the thermal stability of a material through monitoring alterations in sample mass under controlled heating conditions.^58^ Compounds designed for utilization in NLO applications are required to demonstrate the desired nonlinearity while displaying a minimum sensitivity to temperature variations.^59,60^ Two representative chromophores **18** and **24** synthesized within the scope of the study and designated for NLO measurements were selected for TGA measurements (Figure 5). Both chromophores **18** and **24** started to lose weight around 100 °C, which persisted until 690 °C. Compound **18** reached 50% weight loss at 603 °C, while compound **24** achieved this point at 590 °C. When comparing compounds **18** and **24**, the anticipated rise in thermal stability attributed to the greater mass of compound **24** is counterbalanced by the projected decline in thermal stability owing to the increased conjugation (resulting in a decreased band gap) within its structure. Compound **18**, characterized by a higher band gap, demonstrates slightly greater thermal stability compared to compound **24**.

### Electrochemistry

**Figure 6:**
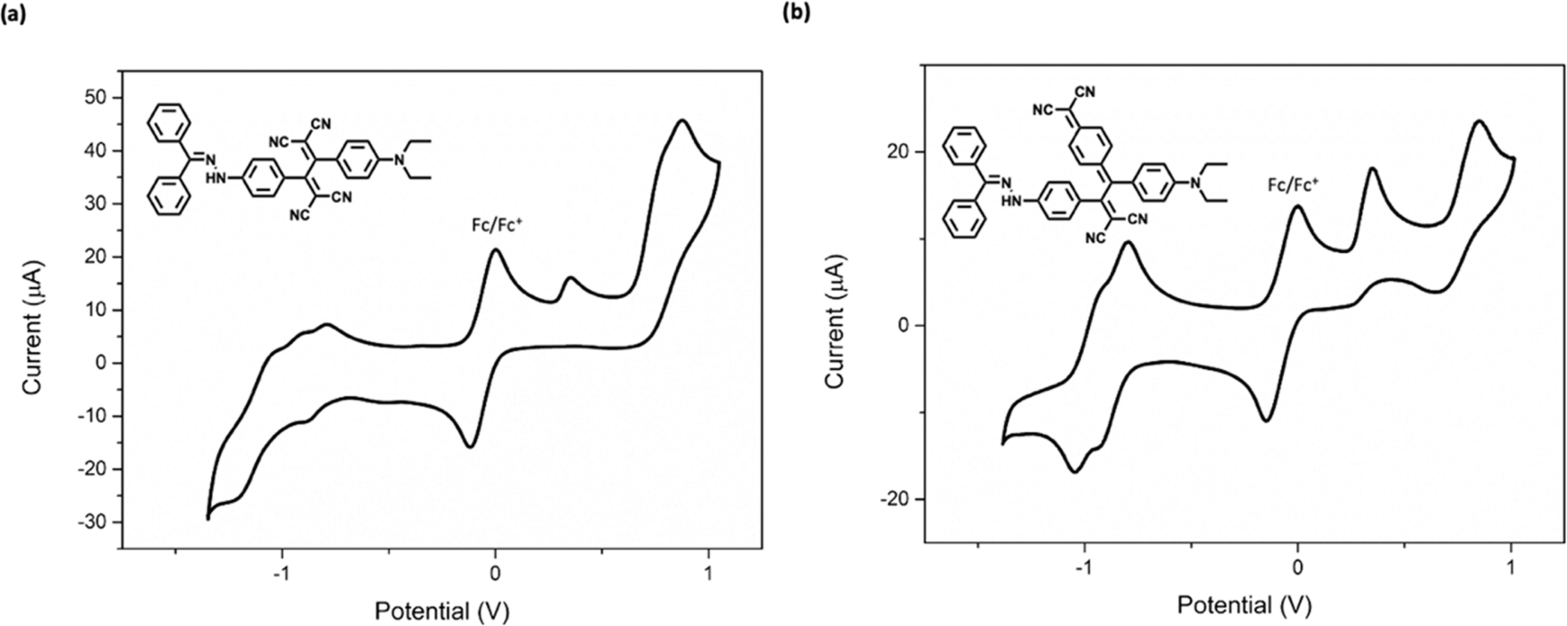
Cyclic voltammograms of the representative NLOphores **21** and **27** at a scan rate of 100 mVs^–1^ in DCM + 0.1 M Bu_4_NPF_6_. All potentials are indicated versus ferrocene/ferrocenium redox couple used as an internal reference (given as IUPAC convention).

Electrochemical behaviors of the corresponding compounds **17**–**21** and **23**–**27** were recorded by cyclic voltammetry (CV) technique (Figures 6 and S58–S65 in the SI). The results of two different series of compound groups, **17**–**21** and **23**–**27**, obtained using TCNE and TCNQ, respectively, were compared with each other. All compounds **17**–**20** except **21** in the first series give a well-defined irreversible oxidation peak at around +0.90 V, while **21** shows two oxidation peak potentials occurring at +0.34 and +0.87 V. These oxidation peaks can be assigned to hydrazone and dialkylaniline donor groups present in the structure of compound **21**.^61,62^ Compounds **19** and **21** possess two reduction peaks at around −0.80 and −1.20 V while a single reduction peak was observed for the others. These two observed reductions are due to the electron uptake of TCBD units and are in agreement with the literature.^62^ Moreover, HOMO–LUMO band gap values were derived from the corresponding CVs, with the results summarized in Table S11. The findings indicate that compounds **23**–**27** have lower band gap values (∼0.9 eV) compared to compounds **17**–**21**, which have values exceeding 1.2 eV.

Similar to the results obtained for the compounds **17**–**21** in the first group, the second group of compounds **23**–**26** obtained with TCNQ, with the exception of compound **27**, show a well-defined irreversible oxidation peak around +0.50 V, while **27** shows two distinct oxidation peak potentials occurring at +0.35 and +0.84 V. The introduction of the dialkylaniline group to **27** as an additional donor group significantly alters the molecule’s redox characteristics. Furthermore, the cathodic peak potentials (*E*_pc_) of compounds **23**–**27** exhibited two distinct peaks. The voltammograms obtained within the scope of the study show the expected oxidation and reduction patterns and are consistent with the literature containing similar chromophore structures.^10,61,62^

### Computational Studies

**Figure 7:**
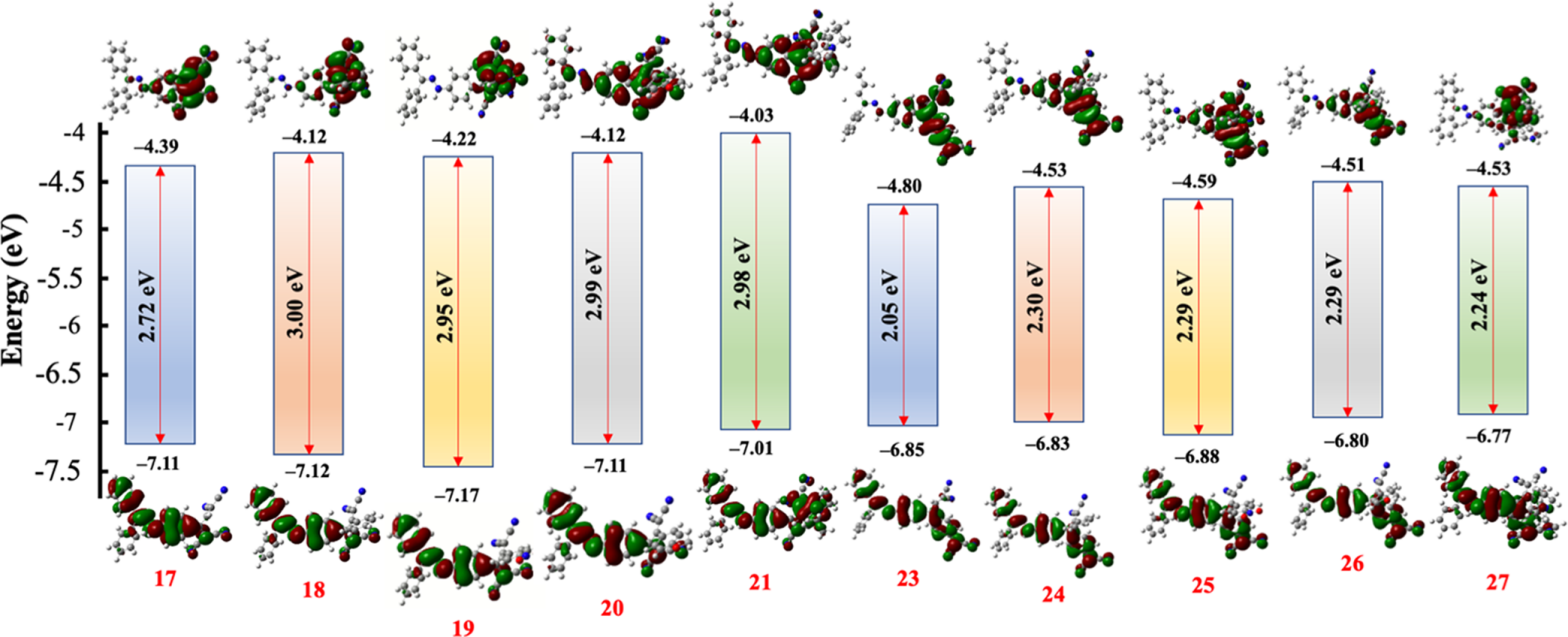
Energy level diagram of the frontier orbitals (HOMOs and LUMOs) of NLOphores **17**–**21** and **23**–**27**.

Computational methods were used to gain further insights into the ICT properties, molecular stability, charge distribution, and NLO properties of the synthesized chromophores. After manual and automated conformational searches, conformations above the global minimum and not exceeding the 3 kcal threshold were selected for Density functional theory (DFT) calculations. DFT calculations were conducted using the CAM-B3LYP/6-31G++(d,p) level of theory, incorporating CPCM solvation in DCM, employing the Gaussian09 software package.^63^ The band gap values were determined through two distinct approaches. These approaches elucidate the direct difference between the energy of the highest occupied molecular orbital (HOMO) and the energy of the lowest unoccupied molecular orbital (LUMO) for the optimized ground state (Δ*E*^direct^), as well as the vertical excitation energy for the lowest singlet excited state (Δ*E*^TD^). While it is acknowledged in the literature that DFT calculations typically underestimate band gaps (Δ*E*^direct^), the focus remains on the trend of band gap variations among the target chromophores, which holds significance.^64^Figure 7 displays the Δ*E*^TD^ values for all synthesized chromophores, which align more closely with the experimental findings (**17**–**21**: 2.72–3.00 eV; **23**–**27**: 2.05–2.30 eV) (Tables S1–S10 in the Supporting Information). When comparing chromophores **17**–**21** with those from **23**–**27**, it is noteworthy that the latter group exhibits lower band gap values. This can be attributed to enhanced conjugation and stronger donor–acceptor interactions within their molecular structures.

**Figure 8:**
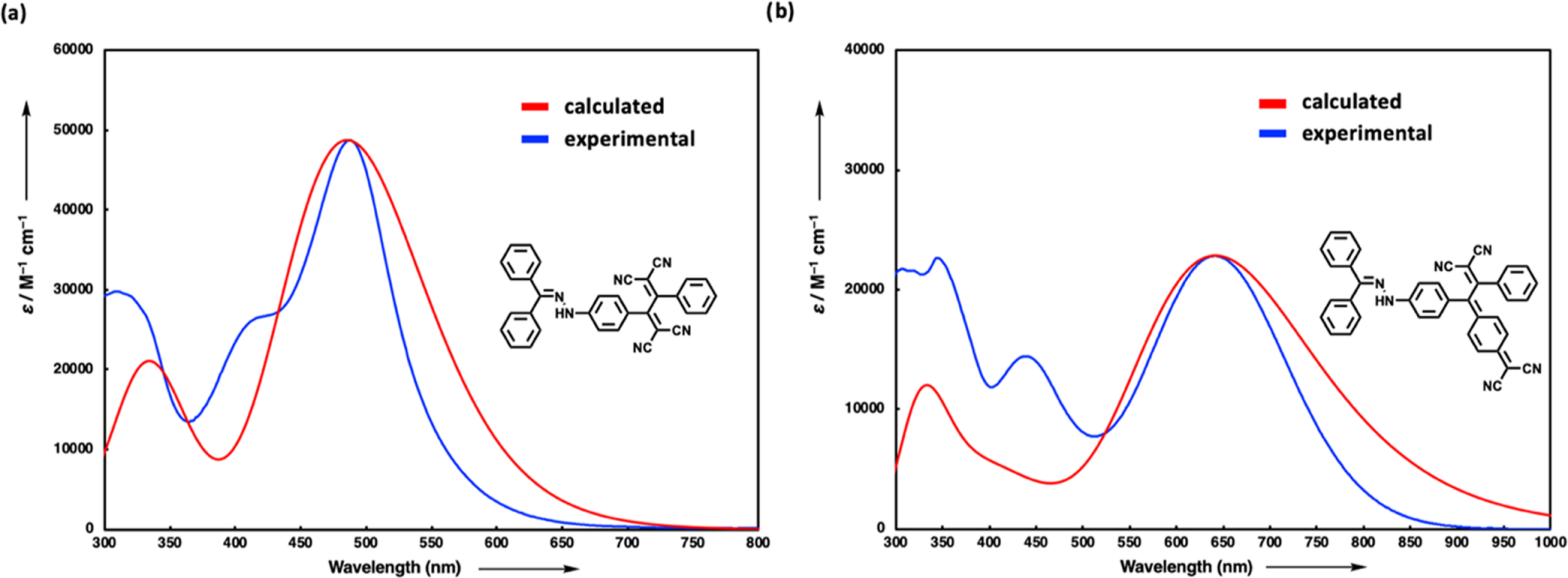
Calculated (red line) and experimental (blue line) UV/vis spectra of (a) **18** and (b) **24** [TD-DFT:CAM-B3LYP/6-31G++(d,p) in DCM].

Upon examination of the HOMO and LUMO of unsubstituted compounds **17** and **23**, it becomes apparent that the LUMO attains a lower energy level than those with electron-poor and electron-rich side groups. Consequently, this leads to reduced band gap values in comparison to other compounds, consistent with the findings from UV/vis absorption measurements. Starting from the optimized structures in DCM, vertical excitation energies and their corresponding absorption wavelengths were computed using TD-DFT using CPCM solvation in DCM at the CAM-B3LYP/6-31G++(d,p) level of theory.^65^ The calculated absorption spectra of the representative chromophores **18** and **24** using TD-DFT methods matched well with the experimental spectra, albeit with calculated extinction coefficients higher than anticipated (scaled by 1.20 for **18** and by 3.05 for **24**) (Figure 8). Additionally, there was a requirement for red-shifting the spectra (by 0.45 eV for **18** and by 0.37 eV for **24**) to align the calculated λ_max_ values with the experimental data.

These deviations between computed excitation energies and experimental absorption maxima are consistent with findings reported in the literature for similar push–pull chromophores.^66−68^ The natural transition orbitals (NTOs) were computed for the electronic transitions with the highest probability percentages at the lowest energy electronic transitions. These orbitals provide insight into the electronic characteristics of these transitions, revealing that the main factor behind the lowest energy absorption bands is primarily ICT interactions (refer to Figure S57 in the Supporting Information). Visualizations of HOMO and LUMO are useful for qualitative assessment of charge-transfer interactions within donor–acceptor systems. As summarized in Table 1, the HOMO tends to localize within the electron-rich hydrazone moiety, while the LUMO predominantly resides in the electron-deficient cyanide groups. This spatial distribution provides additional insights into the ICT mechanisms within these systems. Electrostatic potential map (ESP) visualizations are frequently employed as another method to qualitatively analyze ICT interactions. In these maps, regions of high negative potential typically indicate electron-rich areas, while regions of high positive potential denote electron-deficient regions. When studying ICT, a significant decrease in electron density around the donor moiety accompanied by an increase in electron density around the acceptor moiety suggests electron donation from the donor to the acceptor, indicative of an ICT process. Upon examination of the ESP surfaces of the synthesized chromophores listed in Table 1, it is clear that electron transfer occurs from the donor groups, namely hydrazone and diethylaniline (depicted by blue-colored regions), toward the electron-deficient cyano-rich regions (highlighted by red-colored regions).

**Table 1:**
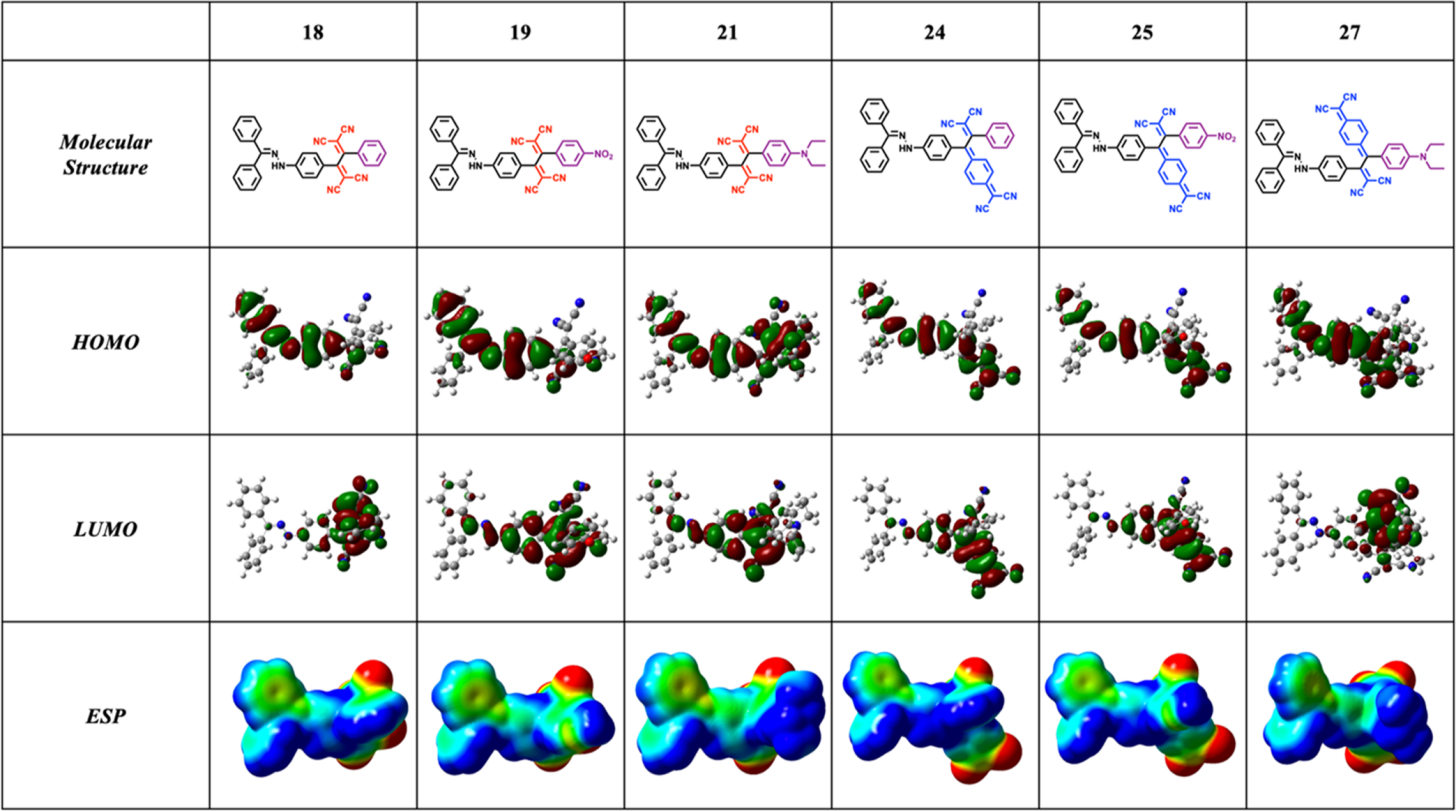
Qualitative Assessment of Charge-Transfer Interactions Using Frontier Orbital Visualizations and Electrostatic Potential Maps [−0.03 au (red) to 0.03 au (blue), DFT: CAM-B3LYP/6-31G++(d,p)] of Representative NLOphores **18**, **19**, **21**, **24**, **25**, and **27**

Donor–acceptor systems have attracted particular attention due to their remarkable NLO capabilities resulting from ICT characteristics. In this study, we have synthesized a range of D–A type chromophores using click-type [2 + 2] CA-RE reactions. After successfully obtaining the desired structures, we conducted comprehensive investigations, encompassing both theoretical calculations and experimental analyses, to explore their NLO properties. Chromophore stability is essential for their effectiveness in NLO applications.^13,69^ Theoretical calculations started with the parameters closely related to molecular stability and reactivity: electric dipole moment (μ), band gap (Δ*E*), electronegativity (χ), global chemical hardness (η) and softness (σ).
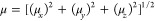
1
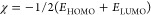
2
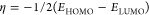
3
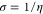
4
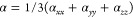
5
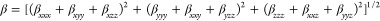
6The electric dipole moment simply measures the distribution of charge within a molecule (eq 1). The balanced charge distribution is often preferred since it minimizes interactions with neighboring molecules and increase stability while preserving the desired optical properties. As indicated in Table 2, chromophores **17**–**21** exhibit lower electric dipole moment values (μ = 15.7961–20.9967 D) compared to chromophores **23**–**27** (μ = 18.8103–30.2411 D). This difference is attributed to the enhanced efficiency of charge transfer interactions between the donor and acceptor groups present in the second group **23**–**27**. A comparable pattern is observed in the band gap values when comparing both groups **17**–**21** (Δ*E*^TD^ = 2.72–3.00 eV) and **23**–**27** (Δ*E*^TD^ = 2.05–2.30 eV). Electronegativity provides insight into a molecule’s ability to withdraw electrons, and it can be computed from the energies of the HOMO and LUMO using eq 2. An increase in this parameter can influence the reactivity of chemical bonds. As anticipated, the products **23**–**27** (χ = 4.72–4.95 eV) obtained with TCNQ exhibit higher electronegativity values compared to those **17**–**21** (χ = 4.47–4.86 eV) obtained with TCNE. Chemical hardness indicates a molecule’s tolerance against alterations in electron density.^70^ It describes how resistant a molecule is to gaining or losing electrons. Larger hardness values suggest greater stability, consequently reducing the molecule’s reactivity (eq 3). Softness, on the other hand, is the inverse of chemical hardness, with molecules possessing higher softness values typically exhibiting increased reactivity (eq 4). A soft molecule can easily accept or donate electrons and is therefore more reactive in chemical reactions. When comparing compounds **17**–**21** (η = 2.31–2.55 eV and σ = 0.39–0.43 eV^–^) and **23**–**27** (η = 1.90–2.08 eV and σ = 0.48–0.53 eV^–^) in terms of global hardness and softness values, it is anticipated that **23**–**27** will demonstrate higher reactivity compared to **17**–**21**. The NLO properties of molecules are significantly influenced by their molecular geometry, which is directly linked to the electronic structure of molecules.^71^ Upon examining the average polarizability and first hyperpolarizability values calculated via eqs 5 and 6, respectively, a noticeable trend emerges that correlates with the variations in band gaps. The average polarizability and first hyperpolarizability values of NLOphores **23**–**27**, characterized by a more pronounced D–A structure, exceed those of NLOphores **17**–**21** obtained through TCNE reactions. The average polarizability values exhibit an approximately 1.5-fold increase, while the first hyperpolarizability values show an almost 3-fold increase when comparing NLOphores **17**–**21** (α_(tot)_ = 81.184–117.539 × 10^–24^ esu and (β_(tot)_ = 261.841–278.984 × 10^–30^ esu)) to **23**–**27** (α_(tot)_ = 125.997–161.495 × 10^–24^ esu and (β_(tot)_ = 534.660–803.847 × 10^–30^ esu)). Among all synthesized chromophores, the highest average polarizability value belongs to compound **27** (α_(tot)_ = 161.495 × 10^–24^ esu) with the highest dipole moment (μ = 30.2411 D), while the highest first hyperpolarizability value belongs to compound **23** (β_(tot)_ = 803.847 × 10^–30^ esu) with the lowest band gap (Δ*E*^TD^ = 2.05).

**Table 2:** Electric Dipole Moment (μ), HOMO-LUMO Gap (Δ*E* and Δ*E*^TD^), Electronegativity (χ), Global Chemical Hardness (η), Softness (σ), Average Polarizability [α_(tot)_], and First Hyperpolarizability [β_(tot)_], Calculated at the CAM-B3LYP/6-31G++(d,p) Theory in CH_2_Cl_2_ (CPCM)

	**μ** (D)	***E***_**HOMO**_**(eV)**	***E***_**LUMO**_**(eV)**	**Δ***E*^**direct**^**(eV)**	**Δ***E*^**TD**^**(eV)**	**χ (eV)**	**η (eV)**	**σ (eV**^**–**^**)**	**α**_**(tot)**_**(×10**^**–24**^**esu)**	**β**_**(tot)**_**(×10**^**–30**^**esu)**
**17**	16.3609	–7.11	–2.48	4.63	2.72	4.80	2.32	0.43	81.184	267.677
**18**	16.5704	–7.12	–2.04	5.08	3.00	4.58	2.54	0.39	97.240	261.897
**19**	15.7961	–7.17	–2.55	4.62	2.95	4.86	2.31	0.43	100.863	278.984
**20**	15.8527	–7.11	–2.01	5.10	2.99	4.56	2.55	0.39	103.099	261.841
**21**	20.9967	–7.01	–1.93	5.08	2.98	4.47	2.54	0.39	117.539	278.029
**23**	20.5175	–6.85	–3.05	3.80	2.05	4.95	1.90	0.53	125.997	803.847
**24**	21.2200	–6.83	–2.67	4.16	2.30	4.75	2.08	0.48	138.459	714.478
**25**	18.8103	–6.88	–2.78	4.10	2.29	4.83	2.05	0.49	140.938	720.432
**26**	21.0718	–6.80	–2.65	4.15	2.29	4.72	2.08	0.48	144.704	723.041
**27**	30.2411	–6.77	–2.70	4.07	2.24	4.74	2.04	0.49	161.495	534.660

### NLO Studies

Typically, NLO responses are closely linked to the efficiency of ICT, which can be fine-tuned by altering the strength of the donor and acceptor groups. Due to the straightforward and effective nature of this method, most small organic^12,21,72,73^ and polymeric^74−76^ second-order NLO materials are constructed using a donor-π-conjugated bridge-acceptor framework. The theoretical calculations, which suggested a high first hyperpolarizability values for all synthesized chromophores, were the motivation for us to perform the corresponding experimental measurements. The NLO activity of the chromophores **17**–**21** and **23**–**27** was evaluated using the electric field-induced second harmonic generation (EFISHG) technique, which allows for the determination of the scalar μβ product, where μ represents the dipole moment and β denotes the vector part of the hyperpolarizability tensor.^77−79^ The μβ values are listed in Table 3. All measurements were conducted in a CHCl_3_ solution using a nonresonant incident wavelength of 1907 nm. The solutions were prepared within the range of 10^–2^–10^–3^ M and the measurements were conducted using a Raman-shifted Nd:YAG laser source emitting at λ = 1907 nm. Positive μβ values were obtained for all chromophores, aligning with their positive solvatochromism and ICT properties. This suggests that both the ground and excited states are polarized in the same direction, indicating a higher level of polarization in the excited state compared to the ground state. Due to the ±10% margin of error inherent in the μβ values determined by the EFISH method employed, meaningful comparison within the two groups derived from the TCNE and TCNQ reactions becomes challenging. As a result, interpreting the effect of the side groups on μβ values becomes difficult. However, upon comparing these two groups (**17**–**21** and **23**–**27**) with each other, there is a significant increase in the measured μβ values for the compounds in the second group **23**–**27** (520–1000 × 10^–48^ esu in **17**–**21**, 3150–5300 × 10^–48^ esu in **23**–**27**). While the calculated μβ values in CHCl_3_ were found to be overestimated in comparison to the measured values for both groups, the general trend between the two groups remained consistent. The two highest μβ values in both groups are attributed to NLOphores containing diethylaniline side groups (1000 × 10^–48^ esu for **21** and 5200 × 10^–48^ esu for **27**), aligning well with theoretical calculations. This result is expected, considering diethylaniline’s status as the strongest donor group among the side groups.

**Table 3:** Calculated and Measured μβ Values of Chromophores **17**–**21** and **23**–**27**

**compound**	**μ**^a^**(Debye)**	**β**^a^**(**10^**–30**^ esu)	**μβ(10**^**–48**^**esu**^**2**^·cm)^b^	**μβ(**10^**–48**^ esu)^c^
**17**	15.9899	235	3758	840
**18**	16.0944	227	3653	820
**19**	15.4348	245	3782	520
**20**	15.3865	227	3493	900
**21**	20.2361	238	4816	1000
**23**	19.7819	680	13,452	5300
**24**	20.4653	601	12,300	3150
**25**	18.1885	610	11,095	4800
**26**	20.3186	608	12,354	4450
**27**	28.9238	451	13,045	5200

a Calculated at the DFT CAM-B3LYP/6-31G++(d,p) level in CHCl_3_.

b 1D = 1 × 10^–18^ esu·cm.

c μβ (2ω) at 1907 nm in CHCl_3_, molecular concentrations used for the measurements were in the range of 10^–3^ to 10^–2^ M, μβ ± 10%.

Upon reviewing recent literature on EFISH method, it is evident that the μβ values obtained within the scope of our study are quite promising. The first group **17**–**21** yields result closely aligned with measurements conducted for Disperse Red 1 (500 × 10^–48^ esu), a recognized benchmark for EFISH.^21^ Conversely, the second group **23**–**27** exhibits μβ values significantly higher than first group **17**–**21**, demonstrating superior performance compared to the majority of values reported for push–pull chromophores in existing literature.^22,59,77,80,81^

## Conclusions

In this study, reactions of electron-rich alkynes with TCNE and TCNQ produced two distinct groups of NLOphores in moderate to high yields. Hydrazones were evaluated for the first time as substrates for the synthesis of target NLOphore structures in CA-RE reactions, which are known for their limited substrate diversity. Hydrazone-based NLOphores have maximum absorption wavelengths (λ_max_) between 473 and 725 nm. The synthesized nonplanar D–A systems were extensively analyzed through a combination of experimental and computational methods, revealing intriguing electrochemical, photophysical, and second-order nonlinear optical properties. Compounds from both groups exhibit positive solvatochromism properties, consistent with their ICT characteristics. Besides the computational approach, experimental NLO measurements conducted using the EFISHG technique showed that the studied structures demonstrated significant NLO responses, μβ values ranging from 520 × 10^–48^ esu to 5300 × 10^–48^ esu. We are currently exploring the adaptability of the synthetic method used in this study to produce chromophore structures with enhanced NLO properties.

## Experimental Section

### General

Commercially available chemicals were purchased and no further purification steps were undertaken. Compounds **2**, **9**, **10**, **11** were synthesized following established procedures outlined in the literature.^51−54^ Solvents (such as dichloromethane, hexanes, and ethyl acetate) utilized for extraction or column chromatography were distilled prior to use. Sonogashira cross-coupling reactions were conducted in a nitrogen atmosphere using dry glassware. Column chromatography (CC) using SiO_2_-60 mesh was employed to purify the target compounds. Analytical thin-layer chromatography (TLC) was performed on aluminum sheets coated with 0.2 mm silica gel 60 F254, and visualization was achieved using a UV lamp (254 or 366 nm). The solvents were evaporated under vacuum at temperatures ranging from 25 to 60 °C and pressures between 900 and 10 mbar. ^1^H and ^13^C{^1^H} nuclear magnetic resonance (NMR) spectra were acquired at frequencies of 400 MHz for ^1^H and 100 MHz for ^13^C{^1^H}, respectively. Structural assignments were corroborated using supplementary data obtained from gCOSY, gHSQC, and gHMBC experiments.

Chemical shifts (δ) are expressed in parts per million (ppm) relative to tetramethylsilane (TMS), utilizing the residual deuterated solvent signal as an internal reference (CDCl_3_: δ_H_ = 7.26 ppm, δ_C_ = 77.0 ppm). In ^1^H NMR spectroscopy, resonance multiplicity is denoted as s (singlet), d (doublet), t (triplet), q (quartet), quint (quintet), sext (sextet), sept (septet), m (multiplet), and br. (broad). Coupling constants (*J*) are provided in hertz (Hz). Additionally, all spectra were acquired at room temperature. High-resolution mass spectrometry (HR-MS) analysis was conducted by the mass spectrometry service at the Central Laboratory of Middle East Technical University, Turkey. Masses are presented in units of mass-to-charge ratio (*m*/*z*) as the molecular ion, represented as [M + H]^+^. Electrochemical measurements were performed with Gamry Interface 1010E potentiostat/galvanostat in a cell equipped with three electrodes. A glassy carbon electrode (3.00 mm in diameter) was used for the working electrode. The working electrode was polished with 0.05 μm alumina slurry and then, sonicated within ethanol to remove the disturbing particles before each experiment. A platinum wire as the counter electrode and an Ag wire as the pseudo-reference electrode were employed. 0.1 M tetrabutylammonium hexafluorophosphate (Bu_4_NPF_6_) was used as a supporting electrode. The molecules were dissolved in 0.1 M Bu_4_NPF_6_ containing DCM. Before performing the electrochemical tests, all solutions were deaerated by the bubbling of pure nitrogen gas to eliminate any dissolved oxygen. All potentials are measured against the Fc/Fc^+^ ion as an internal redox reference at a scan rate of 0.1 V s^–1^. UV/vis spectra were obtained using a T80+ UV/vis spectrophotometer. Measurements were taken in a 1 cm quartz cuvette at 298 K. The absorption maxima (λ_max_) are given in nm, with the extinction coefficient (ε) in M^–1^ cm^–1^ provided in parentheses. A representative sample from each of the two different chromophore groups was dissolved in DCM at concentrations ranging from 1 × 10^–5^ to 3.2 × 10^–5^ to verify compliance with the Beer–Lambert law. After confirmation, UV/vis measurements for all chromophores were conducted in DCM at a concentration of 2 × 10^–5^ M.

#### Synthesis of Compound **4**

Benzophenone (**3**) (85 mg, 0.47 mmol) was dissolved in 5 mL of ethanol and combined with a solution containing 4-iodohydrazine (**2**) (219 mg, 0.94 mmol) in 3 mL of deionized water. The resulting mixture was stirred at room temperature for 1 h. After stirring, the reaction mixture was subjected to extraction with ethyl acetate (3 × 50 mL), followed by drying over MgSO_4_ and filtration. The solvent was then evaporated under reduced pressure, and column chromatography (CC) was carried out (SiO_2_; 9:1 hexanes/ethyl acetate). Yield: 110 mg; brown oil; 59%. *R*_*f*_ = 0.52 (SiO_2_; hexanes:EtOAc 9:1); ^1^H NMR (400 MHz, CDCl_3_, 298 K); δ = 7.62–7.55 (m, 5H), 7.51 (quasi d, *J* = 8.6 Hz, 2H), 7.48 (br. s, 1H), 7.36–7.30 (m, 5H), 6.87 ppm (quasi d, *J* = 8.6 Hz, 2H); ^13^C{^1^H} NMR (100 MHz, CDCl_3_, 298 K); δ = 145.2, 144.4, 138.2, 137.9, 132.6, 129.9, 129.5, 129.2, 128.42, 128.35, 126.7, 115.2, 81.4 ppm. HRMS (ESI-TOF) *m*/*z*: [M + H]^+^ calcd for C_19_H_16_N_2_I^+^ 399.0358; found: 399.0356.

#### Synthesis of Compound **6**

The aryl iodide **4** (614 mg, 1.54 mmol, 1 equiv), along with bis(triphenylphosphine)palladium(II) dichloride (32.5 mg, 0.046 mmol, 0.03 equiv) and copper iodide (8.8 mg, 0.046 mmol, 0.03 equiv), were introduced into a two-necked round-bottom flask and stirred for 30 min under an inert nitrogen atmosphere. Subsequently, a solution containing triethylamine (5 mL per 1.0 mmol) and THF (4 mL per 1.0 mmol) was injected into the flask via syringe, followed by an additional 15 min of degassing with nitrogen. Trimethylsilyl acetylene (**5**) (182 mg, 1.85 mmol, 1.2 equiv) was then added to the reaction mixture. After overnight stirring at 25 °C, the reaction was terminated by quenching with water, followed by extraction with dichloromethane (3 × 50 mL), drying over MgSO_4_, and filtration. Removal of the solvent under reduced pressure yielded the coupling product **6**, which was purified by column chromatography (CC) (SiO_2_; 9:1 hexanes/ethyl acetate). Yield: 436 mg; yellow oil; 77%. *R*_*f*_ = 0.57 (SiO_2_; 9:1 hexanes/ethyl acetate); ^1^H NMR (400 MHz, CDCl_3_, 298 K); δ = 7.60–7.54 (m, 5H), 7.53 (br. s, 1H), 7.36 (quasi d, *J* = 8.6 Hz, 2H), 7.34–7.29 (m, 5H), 6.99 (quasi d, *J* = 8.6 Hz, 2H), 0.23 ppm (s, 9H); ^13^C{^1^H} NMR (100 MHz, CDCl_3_, 298 K); δ = 145.4, 144.6, 138.2, 133.4, 132.6, 129.9, 129.6, 129.2, 128.5, 128.4, 126.7, 114.1, 112.7, 106.1, 92.1, 0.28 ppm; HRMS (ESI-TOF) *m*/*z*: [M + H]^+^ calcd for C_24_H_25_N_2_Si^+^ 369.1787; found: 369.1787.

#### Synthesis of Compound **7**

Compound **6** (387 mg, 1.05 mmol, 1 equiv) was dissolved in 25 mL of methanol, followed by the addition of potassium carbonate (479 mg, 3.47 mmol, 3.30 equiv) to the solution. After filtration, evaporation, and purification by column chromatography (CC) (SiO_2_; eluent: hexanes/ethyl acetate), alkyne **7** was isolated. Yield: 261 mg; yellow oil; 84%. *R*_*f*_ = 0.43 (SiO_2_; 9:1 hexanes/ethyl acetate); ^1^H NMR (400 MHz, CDCl_3_, 298 K); δ = 7.60–7.55 (m, 5H), 7.54 (br s., 1H), 7.38 (quasi d, *J* = 8.6 Hz, 2H), 7.34–7.31 (m, 5H), 7.02 (quasi d, *J* = 8.6 Hz, 2H), 3.00 ppm (s, 1H); ^13^C{^1^H} NMR (100 MHz, CDCl_3_, 298 K); δ = 145.6, 144.9, 138.1, 133.5, 132.6, 129.9, 129.6, 129.2, 128.5, 128.4, 126.8, 112.9, 112.8, 84.5, 75.6 ppm; HRMS (ESI-TOF) *m*/*z*: [M + H]^+^ calcd for C_21_H_17_N_2_^+^ 297.1392; found 297.1392.

#### Synthesis of Compounds **12**–**15**

The aryl iodide **4** (1.00 mmol, 1 equiv), along with bis(triphenylphosphine)palladium(II) dichloride (0.030 mmol, 0.03 equiv) and copper iodide (0.030 mmol, 0.03 equiv), were introduced into a two-necked round-bottom flask and stirred for 30 min under an inert nitrogen atmosphere. Subsequently, a solution containing triethylamine (5 mL per 1.0 mmol) and THF (4 mL per 1.0 mmol) was injected into the flask via syringe, followed by an additional 15 min of degassing with nitrogen. Terminal alkynes **8**–**11** (1.50 mmol, 1.5 equiv) was then added to the reaction mixture. After overnight stirring at 25 °C, the reaction was terminated by quenching with water, followed by extraction with dichloromethane (3 × 50 mL), drying over MgSO_4_, and filtration. Removal of the solvent under reduced pressure yielded the coupling products **12**–**15**, which were purified by column chromatography (CC) (SiO_2_; 9:1 hexanes/ethyl acetate).

##### Compound **12**

Yield: 175 mg; brown oil; 47%. *R*_*f*_ = 0.40 (SiO_2_; 9:1 hexanes/ethyl acetate); ^1^H NMR (400 MHz, CDCl_3_, 298 K); δ = 7.62–7.53 (m, 6H), 7.51 (d, *J* = 6.6 Hz, 2H), 7.43 (quasi d, *J* = 8.6 Hz, 2H), 7.36–7.29 (m, 8H), 7.06 ppm (quasi d, *J* = 8.6 Hz, 2H); ^13^C{^1^H} NMR (100 MHz, CDCl_3_, 298 K); δ = 145.4, 144.5, 138.2, 133.0, 132.6, 131.5, 129.9, 129.6, 129.2, 128.5, 128.42, 128.38, 127.9, 126.7, 124.0, 114.3, 112.9, 90.3, 88.0 ppm. HRMS (ESI-TOF) *m*/*z*: [M + H]^+^ calcd for C_27_H_21_N_2_^+^ 373.1705; found 373.1705.

##### Compound **13**

Yield: 354 mg; orange solid; 85%. *R*_*f*_ = 0.65 (SiO_2_; 9:1 hexanes/ethyl acetate); m.p.= 220–222 °C; ^1^H NMR (400 MHz, CDCl_3_, 298 K); δ = 8.20 (quasi d, *J* = 8.7 Hz, 2H), 7.66–7.53 (m, 8H), 7.45 (d, *J* = 8.7 Hz, 2H), 7.39–7.29 (m, 5H), 7.08 ppm (d, *J* = 8.7 Hz, 2H); ^13^C{^1^H} NMR (100 MHz, CDCl_3_, 298 K); δ = 146.6, 146.1, 145.3, 138.0, 133.4, 132.5, 131.9, 131.2, 129.9, 129.7, 129.2, 128.7, 128.4, 126.8, 123.8, 113.0, 112.8, 96.4, 86.8 ppm; HRMS (ESI-TOF) *m*/*z*: [M – H]^−^ calcd for C_27_H_18_N_3_O_2_^–^ 416.1399; found 416.1399.

##### Compound **14**

Yield: 177 mg; yellow-orange oil; 44%. R_*f*_ = 0.40 (SiO_2_; 9:1 hexanes/ethyl acetate); ^1^H NMR (400 MHz, CDCl_3_, 298 K); δ = 7.62–7.56 (m, 5H), 7.54 (br. s, 1H), 7.44 (d, *J* = 8.7 Hz, 2H), 7.41 (d, *J* = 8.7 Hz, 2H), 7.33–7.28 (m, 5H), 7.05 (d, *J* = 8.7 Hz, 2H), 6.86 (d, *J* = 8.7 Hz, 2H), 3.82 ppm (s, 3H); ^13^C{^1^H} NMR (100 MHz, CDCl_3_, 298 K); δ = 159.4, 145.3, 144.3, 138.2, 132.9, 132.8, 132.7, 129.9, 129.5, 129.2, 128.40, 128.37, 126.7, 116.1, 114.6, 114.1, 112.9, 88.8, 87.9, 55.4 ppm; HRMS (ESI-TOF) *m*/*z*: [M + H]^+^ calcd for C_28_H_23_N_2_O^+^ 403.1810; found 403.1811.

##### Compound **15**

Yield: 236 mg; yellow oil; 53%. *R*_*f*_ = 0.28 (SiO_2_; 9:1 hexanes/ethyl acetate); ^1^H NMR (400 MHz, CDCl_3_, 298 K); δ = 7.63–7.51 (m, 6H), 7.39 (d, *J* = 8.6 Hz, 2H), 7.37–7.28 (m, 7H), 7.03 (d, *J* = 8.6 Hz, 2H), 6.60 (d, *J* = 8.9 Hz, 2H), 3.37 (q, *J* = 7.0 Hz, 4H), 1.17 ppm (t, *J* = 7.0 Hz, 6H); ^13^C{^1^H} NMR (100 MHz, CDCl_3_, 298 K); δ = 147.4, 145.0, 143.9, 138.3, 132.9, 132.8, 132.6, 129.9, 129.5, 129.3, 128.4, 128.3, 126.7, 115.5, 112.9, 111.4, 109.7, 89.2, 87.6, 44.5, 12.7 ppm; HRMS (ESI-TOF) *m*/*z*: [M + H]^+^ calcd for C_31_H_30_N_3_^+^ 444.2440; found 444.2440.

#### Synthesis of Compounds **17**–**21**

A mixture containing of hydrazone-substituted alkynes **7**, **12**–**15** (0.20 mmol, 1 equiv) and TCNE (0.20 mmol, 1 equiv) in 5 mL of 1,2-dichloroethane was stirred at 25 °C until all starting materials were fully consumed according to TLC analysis (approximately 24 h). After evaporation and column chromatography CC (SiO_2_; CH_2_Cl_2_), the desired products **17**–**21** were obtained.

##### Compound **17**

Yield: 27 mg; dark-purple solid; 32%. *R*_*f*_ = 0.68 (SiO_2_; CH_2_Cl_2_); m.p.= 139–141 °C; ^1^H NMR (400 MHz, CDCl_3_, 298 K); δ = 8.03 (s, 1H), 8.00 (br. s, 1H), 7.66–7.57 (m, 5H), 7.46 (quasi d, *J* = 9.0 Hz, 2H), 7.39–7.31 (m, 5H), 7.25–7.17 ppm (m, 2H); ^13^C{^1^H} NMR (100 MHz, CDCl_3_, 298 K); δ = 160.2, 155.3, 149.9, 149.7, 137.3, 132.1, 131.7, 130.2, 130.1, 129.6, 129.0, 128.6, 127.3, 121.5, 113.9, 112.9, 112.4, 111.7, 109.0, 97.9, 83.6 ppm; UV/vis (CH_2_Cl_2_): λ_max_ (ε) = 359 (21,000), 584 nm (6100 M^–1^ cm^–1^); IR (ATR): ν̃ = 3289 (w), 2218 (m), 1598 (s) cm^–1^; HRMS (ESI-TOF) *m*/*z*: [M + H]^+^ calcd for C_27_H_17_N_6_^+^ 425.1515; found: 425.1515.

##### Compound **18**

Yield: 76 mg; dark-red solid; 75%. *R*_*f*_ = 0.68 (SiO_2_; CH_2_Cl_2_); m.p.= 203–205 °C; ^1^H NMR (400 MHz, CDCl_3_, 298 K); δ = 8.13 (br. s, 1H), 7.78 (d, *J* = 9.2 Hz, 2H), 7.74–7.70 (m, 2H), 7.66–7.53 (m, 8H), 7.39–7.31 (m, 5H), 7.20 ppm (m, 2H); ^13^C{^1^H} NMR (100 MHz, CDCl_3_, 298 K); δ = 168.9, 164.2, 150.7, 150.0, 137.1, 134.6, 132.5, 131.9, 131.6, 130.3, 130.1, 130.0, 129.7, 129.6, 128.9, 128.6, 127.4, 121.8, 113.9, 113.6, 112.8, 112.0, 111.3, 87.6, 78.1 ppm; UV/vis (CH_2_Cl_2_): λ_max_ (ε) = 287 (29,800), 309 (29,700), 488 nm (48,700 M^–1^ cm^–1^); IR (ATR): ν̃ = 3283 (w), 2219 (m), 1599 (s) cm^–1^; HRMS (ESI-TOF) *m*/*z*: [M + H]^+^ calcd for C_33_H_21_N_6_^+^ 501.1828; found: 501.1828.

##### Compound **19**

Yield: 88 mg; dark-red solid; 80%. *R*_*f*_ = 0.48 (SiO_2_; CH_2_Cl_2_); m.p.= 164–166 °C; ^1^H NMR (400 MHz, CDCl_3_, 298 K); δ = 8.38 (d, *J* = 8.9 Hz, 2H), 8.14 (br. s, 1H), 7.84 (d, *J* = 8.9 Hz, 2H), 7.78 (d, *J* = 9.2 Hz, 2H), 7.68–7.55 (m, 6H), 7.41–7.31 ppm (m, 6H); ^13^C{^1^H} NMR (100 MHz, CDCl_3_, 298 K); δ = 166.6, 162.5, 151.4, 150.4, 150.3, 137.2, 137.0, 132.5, 131.5, 130.6, 130.4, 130.1, 129.9, 128.9, 128.6, 127.5, 124.9, 121.0, 114.2, 113.3, 112.9, 111.1, 110.5, 91.5, 77.8 ppm; UV/vis (CH_2_Cl_2_): λ_max_ (ε) = 310 (19,300), 473 nm (22,200 M^–1^ cm^–1^); IR (ATR): ν̃ = 3311 (w), 2219 (m), 1598 (s) cm^–1^; HRMS (ESI-TOF) *m*/*z*: [M + H]^+^ calcd for C_33_H_20_N_7_O_2_^+^ 546.1678; found: 546.1677.

##### Compound **20**

Yield: 82 mg; dark-red solid; 77%. *R*_*f*_ = 0.69 (SiO_2_; CH_2_Cl_2_); m.p.= 129–131 °C; ^1^H NMR (400 MHz, CDCl_3_, 298 K); δ = 8.05 (br. s, 1H), 7.81–7.74 (m, 4H), 7.64–7.57 (m, 5H), 7.37–7.30 (m, 5H), 7.23–7.13 (m, 2H), 7.02 (d, *J* = 8.9 Hz, 2H), 3.91 ppm (s, 3H); ^13^C{^1^H} NMR (100 MHz, CDCl_3_, 298 K); δ = 167.4, 165.0, 150.5, 149.8, 137.2, 132.6, 132.3, 131.7, 130.3, 130.1, 129.7, 129.0, 128.6, 127.4, 124.3, 122.3, 115.6, 113.9, 113.7, 112.9, 112.8, 112.0, 83.1, 78.3, 56.0 ppm (25 out of 26 expected signals observed); UV/vis (CH_2_Cl_2_): λ_max_ (ε) = 334 (22,800), 386 (32,400), 487 nm (47,500 M^–1^ cm^–1^); IR (ATR): ν̃ = 2988 (w), 2220 (m), 1616 (m) cm^–1^; HRMS (ESI-TOF) *m*/*z*: [M + H]^+^ calcd for C_34_H_23_N_6_O^+^ 531.1933; found: 531.1933.

##### Compound **21**

Yield: 85 mg; dark-red solid; 80%. *R*_*f*_ = 0.28 (SiO_2_; CH_2_Cl_2_); m.p.= 201–203 °C; ^1^H NMR (400 MHz, CDCl_3_, 298 K); δ = 8.02 (br. s, 1H), 7.78 (quasi d, *J* = 9.3 Hz, 2H), 7.77 (quasi d, *J* = 9.3 Hz, 2H), 7.65–7.56 (m, 5H), 7.38–7.30 (m, 5H), 7.21–7.07 (m, 2H), 6.67 (quasi d, *J* = 9.3 Hz, 2H), 3.47 (q, *J* = 7.1 Hz, 4H), 1.24 ppm (t, *J* = 7.1 Hz, 6H); ^13^C{^1^H} NMR (100 MHz, CDCl_3_, 298 K); δ = 166.7, 164.5, 152.6, 149.9, 149.5, 137.3, 133.1, 132.6, 131.7, 130.2, 130.1, 129.6, 129.0, 128.6, 127.3, 123.0, 118.3, 114.9, 114.1, 113.8, 113.6, 113.0, 111.9, 78.5, 73.8, 45.2, 12.7 ppm; UV/vis (CH_2_Cl_2_): λ_max_ (ε) = 478 nm (82,500 M^–1^ cm^–1^); IR (ATR): ν̃ = 2987 (w), 2215 (m), 1601 (m) cm^–1^; HRMS (ESI-TOF) *m*/*z*: [M + H]^+^ calcd for C_37_H_30_N_7_^+^ 572.2563; found: 572.2569.

#### Synthesis of Compounds **23**–**27**

A mixture containing of hydrazone-substituted alkynes **7**, **12**–**15** (0.20 mmol, 1 equiv) and TCNQ (0.30 mmol, 1.5 equiv) in 5 mL of 1,2-dichloroethane was stirred at 25 °C (60 °C in an oil bath for substrates **12**–**14**) until all starting materials were fully consumed according to TLC analysis (approximately 24 h). After evaporation and column chromatography CC (SiO_2_; CH_2_Cl_2_), the desired products **23**–**27** were obtained.

##### Compound **23**

Yield: 72 mg; dark-green-black solid; 72%. *R*_*f*_ = 0.36 (SiO_2_; CH_2_Cl_2_); m.p.= 249–251 °C; ^1^H NMR (400 MHz, CDCl_3_, 298 K); δ = 8.19 (s, 1H), 7.88 (br. s, 1H), 7.65–7.55 (m, 5H), 7.50–7.28 (m, 9H), 7.24 (quasi d, *J* = 8.8 Hz, 2H), 7.15 ppm (quasi d, *J* = 8.8 Hz, 2H); ^13^C{^1^H} NMR (100 MHz, CDCl_3_, 298 K); δ = 155.1, 152.9, 148.1, 147.8, 146.3, 137.6, 137.3, 135.6, 133.7, 132.1, 131.8, 131.4, 130.01, 129.95, 129.2, 129.1, 128.5, 127.3, 127.1, 125.8, 114.0, 113.9, 113.7, 110.5, 92.3, 79.1 ppm (26 out of 27 expected signals observed); UV/vis (CH_2_Cl_2_): λ_max_ (ε) = 366 (30,200), 422 (39,000), 725 nm (20,400 M^–1^ cm^–1^); IR (ATR): ν̃ = 3266 (m), 2204 (m), 1562 (s) cm^–1^; HRMS (ESI-TOF) *m*/*z*: [M + H]^+^ calcd for C_33_H_21_N_6_^+^ 501.1828; found: 501.1832.

##### Compound **24**

Yield: 68 mg; dark-green amorphous solid; 59%. *R*_*f*_ = 0.68 (SiO_2_; CH_2_Cl_2_); m.p.= 249–251 °C; ^1^H NMR (400 MHz, CDCl_3_, 298 K); δ = 7.89 (br. s, 1H), 7.64–7.52 (m, 8H), 7.50–7.44 (m, 3H), 7.36–7.30 (m, 5H), 7.27–7.25 (m, 3H), 7.21 (d, *J* = 9.6 Hz, 1H), 7.14 (d, *J* = 8.1 Hz, 2H), 7.03 ppm (d, *J* = 9.6 Hz, 1H); ^13^C{^1^H} NMR (100 MHz, CDCl_3_, 298 K); δ = 172.3, 154.1, 151.1, 149.0, 147.7, 137.4, 135.6, 134.7, 134.3, 134.1, 133.7, 133.3, 131.9, 130.1, 129.8, 129.7, 129.4, 129.0, 128.5, 127.2, 127.1, 126.1, 125.9, 114.24, 114.17, 113.9, 113.0, 112.3, 87.7, 74.7 ppm (30 out of 31 expected signals observed); UV/vis (CH_2_Cl_2_): λ_max_ (ε) = 307 (21,700), 344 (22,600), 439 (14,400), 641 nm (22,800 M^–1^ cm^–1^); IR (ATR): ν̃ = 2988 (w), 2219 (m) cm^–1^; HRMS (ESI-TOF) *m*/*z*: [M + H]^+^ calcd for C_39_H_25_N_6_^+^ 577.2141; found: 577.2141.

##### Compound **25**

Yield: 86 mg; dark-green solid; 69%. *R*_*f*_ = 0.32 (SiO_2_; CH_2_Cl_2_); m.p.= 229–231 °C; ^1^H NMR (400 MHz, CDCl_3_, 298 K); δ = 8.28 (quasi d, *J* = 8.7 Hz, 2H), 7.93 (br. s, 1H), 7.75 (d, *J* = 8.7 Hz, 2H), 7.63–7.56 (m, 5H), 7.46 (d, *J* = 9.9 Hz, 1H), 7.38–7.28 (m, 7H), 7.21 (d, *J* = 8.6 Hz, 2H), 7.14 (d, *J* = 8.1 Hz, 2H), 7.08 ppm (d, *J* = 9.6 Hz, 1H); ^13^C{^1^H} NMR (100 MHz, CDCl_3_, 298 K); δ = 169.6, 153.7, 149.8, 149.4, 149.2, 147.9, 140.2, 137.3, 135.4, 134.1, 134.0, 133.7, 131.8, 130.7, 130.1, 130.0, 129.5, 128.9, 128.5, 127.2, 126.7, 126.6, 126.2, 124.7, 114.04, 113.97, 113.94, 112.2, 111.7, 90.8, 75.9 ppm; UV/vis (CH_2_Cl_2_): λ_max_ (ε) = 310 (31,000), 343 (31,400), 468 (19,600), 657 nm (25,800 M^–1^ cm^–1^); IR (ATR): ν̃ = 3315 (w), 2206 (m), 1578 (s) cm^–1^; HRMS (ESI-TOF) *m*/*z*: [M - H]^−^ calcd for C_39_H_22_N_7_O_2_^–^ 620.1835; found: 620.1832.

##### Compound **26**

Yield: 67 mg; dark- turquoise solid; 55%. *R*_*f*_ = 0.45 (SiO_2_; CH_2_Cl_2_); m.p.= 136–138 °C; ^1^H NMR (400 MHz, CDCl_3_, 298 K); δ = 7.90 (s, 1H), 7.68 (d, *J* = 8.7 Hz, 2H), 7.64–7.56 (m, 5H), 7.50 (d, *J* = 9.5 Hz, 1H), 7.37–7.31 (m, 5H), 7.30–7.23 (m, 3H), 7.17–7.12 (m, 3H), 6.99 (d, *J* = 9.8 Hz, 1H), 6.95 (d, *J* = 8.7 Hz, 2H), 3.86 ppm (s, 3H); ^13^C{^1^H} NMR (100 MHz, CDCl_3_, 298 K); δ = 171.0, 164.3, 154.2, 151.8, 149.0, 147.7, 137.5, 135.6, 134.5, 134.1, 132.9, 132.4, 131.9, 130.1, 129.3, 129.0, 128.5, 127.3, 127.2, 126.9, 125.9, 125.6, 115.3, 114.33, 114.26, 113.9, 113.7, 112.9, 83.9, 74.2, 55.9 ppm (31 out of 32 expected signals observed); UV/vis (CH_2_Cl_2_): λ_max_ (ε) = 349 (43,000), 427 (28,500), 635 nm (48,900 M^–1^ cm^–1^); IR (ATR): ν̃ = 2988 (w), 2204 (m), 1576 (s) cm^–1^; HRMS (ESI-TOF) *m*/*z*: [M + H]^+^ calcd for C_40_H_27_N_6_O^+^: 607.2246; found: 607.2247.

##### Compound **27**

Yield: 114 mg; dark-green solid; 88%. *R*_*f*_ = 0.28 (SiO_2_; CH_2_Cl_2_); m.p.= 214–216 °C; ^1^H NMR (400 MHz, CDCl_3_, 298 K); δ = 7.95 (s, 1H), 7.71 (quasi d, *J* = 9.1 Hz, 2H), 7.64–7.53 (m, 6H), 7.38–7.29 (m, 7H), 7.26–7.22 (m, 1H), 7.10 (dd, *J* = 9.4, 1.8 Hz, 3H), 6.95 (dd, *J* = 9.5, 1.8 Hz, 1H), 6.70 (quasi d, *J* = 9.1 Hz, 2H), 3.47 (q, *J* = 7.1 Hz, 4H), 1.25 ppm (t, *J* = 7.1 Hz, 6H); ^13^C{^1^H} NMR (100 MHz, CDCl_3_, 298 K); δ = 170.4, 154.6, 153.9, 151.3, 149.6, 149.0, 137.3, 136.0, 135.2, 134.9, 132.7, 131.8, 130.8, 130.13, 130.06, 129.5, 128.9, 128.5, 127.2, 125.6, 124.8, 124.3, 123.7, 115.4, 115.3, 114.7, 113.7, 113.5, 112.4, 79.9, 69.5, 45.1, 12.8 ppm; UV/vis (CH_2_Cl_2_): λ_max_ (ε) = 333 (18,500), 427 (41,700), 683 nm (42,200 M^–1^ cm^–1^); IR (ATR): ν̃ = 2928 (w), 2202 (m), 1580 (s) cm^–1^; HRMS (ESI-TOF) *m*/*z*: [M + H]^+^ calcd for C_43_H_34_N_7_^+^ 648.2876; found: 648.2876.

## Data Availability

The data underlying this study are available in the published article and its Supporting Information.

## Supporting Information Available

Theoretical calculations, HR-MS data, copies of UV/vis, ^1^H NMR and ^13^C{^1^H} NMR spectra, cyclic voltammograms, X-ray data (PDF)
