# Supplementary material for: Synthesis and Optical Characterization of Hydrazone-Substituted Push–Pull-Type NLOphores
Source: J Org Chem. 2024 Sep 10;89(18):13192–207. doi: 10.1021/acs.joc.4c01328 (PMC11421011; doi:10.1021/acs.joc.4c01328)
Supplement: Supplementary file 1 — jo4c01328_si_001.pdf [file jo4c01328_si_001.pdf]

Supporting Information

The Journal of Organic Chemistry

Synthesis and Optical Characterization of Hydrazone-Substituted Push-Pull-Type NLOphores

Kübra Erden,[a] Dilek Soyler,[b,c] Alberto Barsella,[d] Onur Şahin,[e] Saniye Soylemez,[b,c] Cagatay Dengiz[a,*]

[a] *Department of Chemistry, Middle East Technical University, 06800 Ankara, Turkey*

[b] *Department of Biomedical Engineering, Necmettin Erbakan University, 42090 Konya, Turkey*

[c] *Science and Technology Research and Application Center (BİTAM), Necmettin Erbakan University, 42090 Konya, Turkey*

[d] *Département d'Optique Ultra-Rapide et Nanophotonique, IPCMS-CNRS, 23 Rue du Loess, BP 43, 67034, Strasbourg, Cedex 2, France*

[e] *Department of Occupational Health & Safety, Faculty of Health Sciences, Sinop University, Sinop 57000, Turkey*

Table of Contents

1. *^{1}H and ^{13}C NMR spectra* S3
2. *High-Resolution Mass Spectrometry (HR-MS) Data* S22
3. *Images* S31
4. *Theoretical Calculations* S32
5. *Electrochemistry* S54
6. *X-ray Diffraction Analysis* S59
7. *UV/Vis Studies* S60
8. *References* S62

1. ^{1}H and ^{13}C NMR spectra

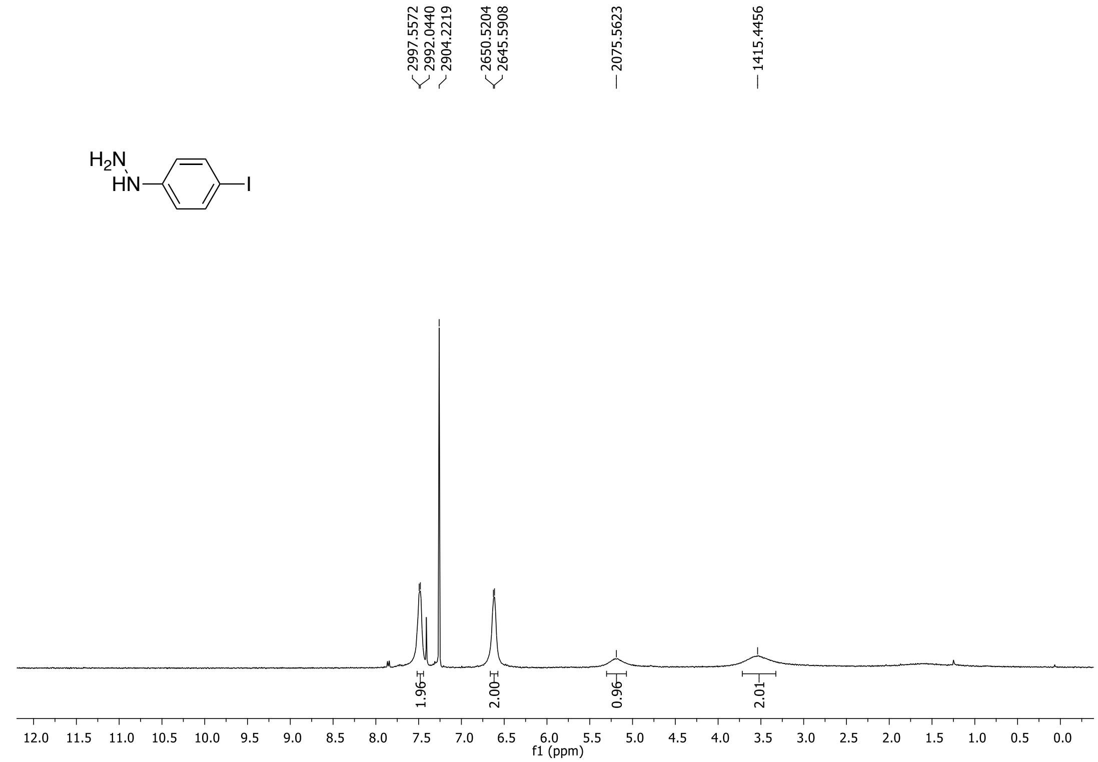

Figure S1. ^{1}H NMR spectrum of **2** in $CDCl_3$ solution (400 MHz).

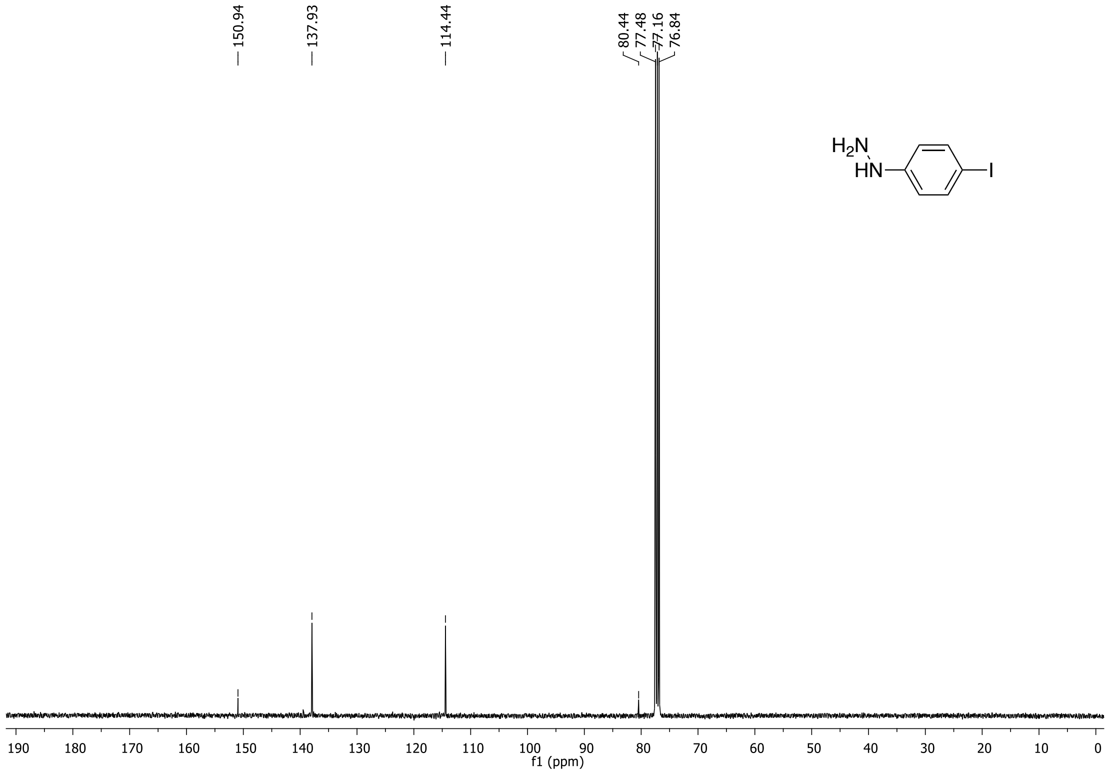

Figure S2. ^{13}C{^{1}H} NMR spectrum of **2** in $CDCl_3$ solution (100 MHz).

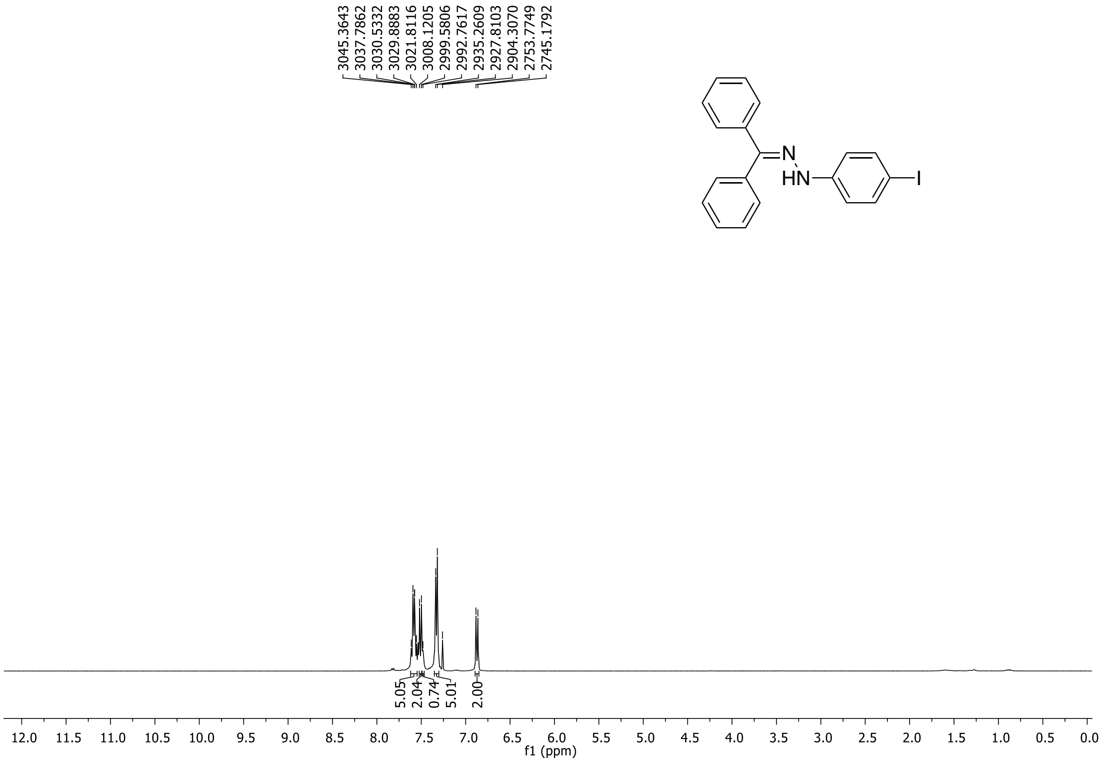

Figure S3.^{1}H NMR spectrum of **4** in $CDCl_3$ solution (400 MHz).

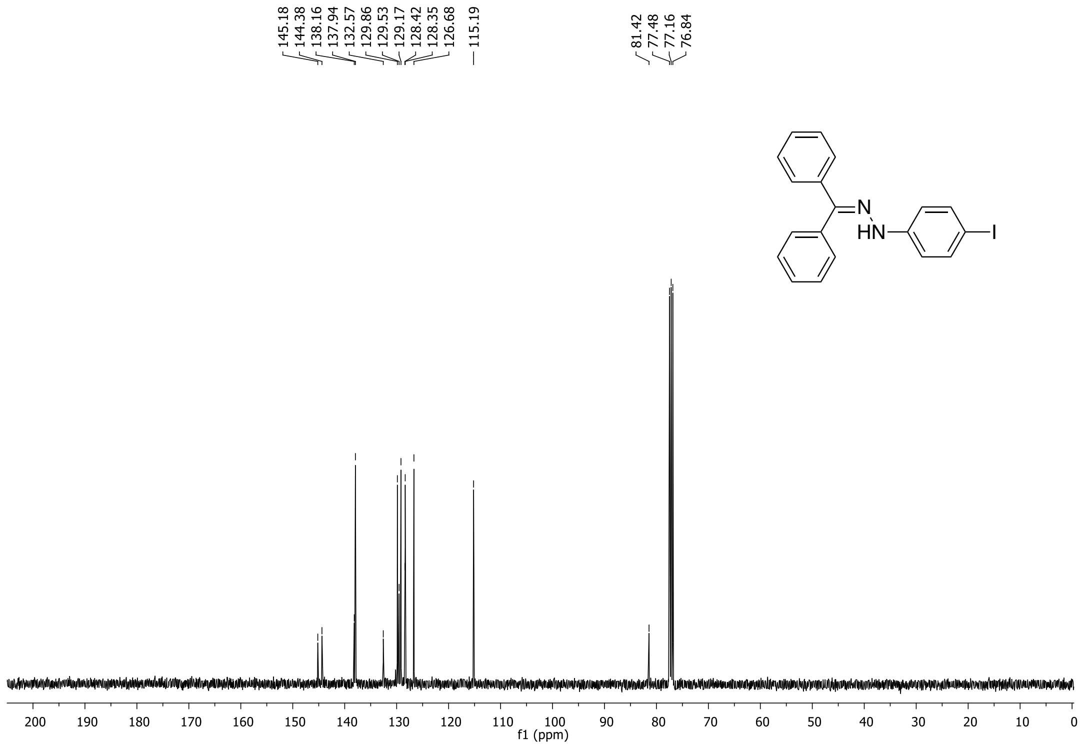

Figure S4. $^{13}C\{^{1}H\}$ NMR spectrum of **4** in $CDCl_3$ solution (100 MHz).

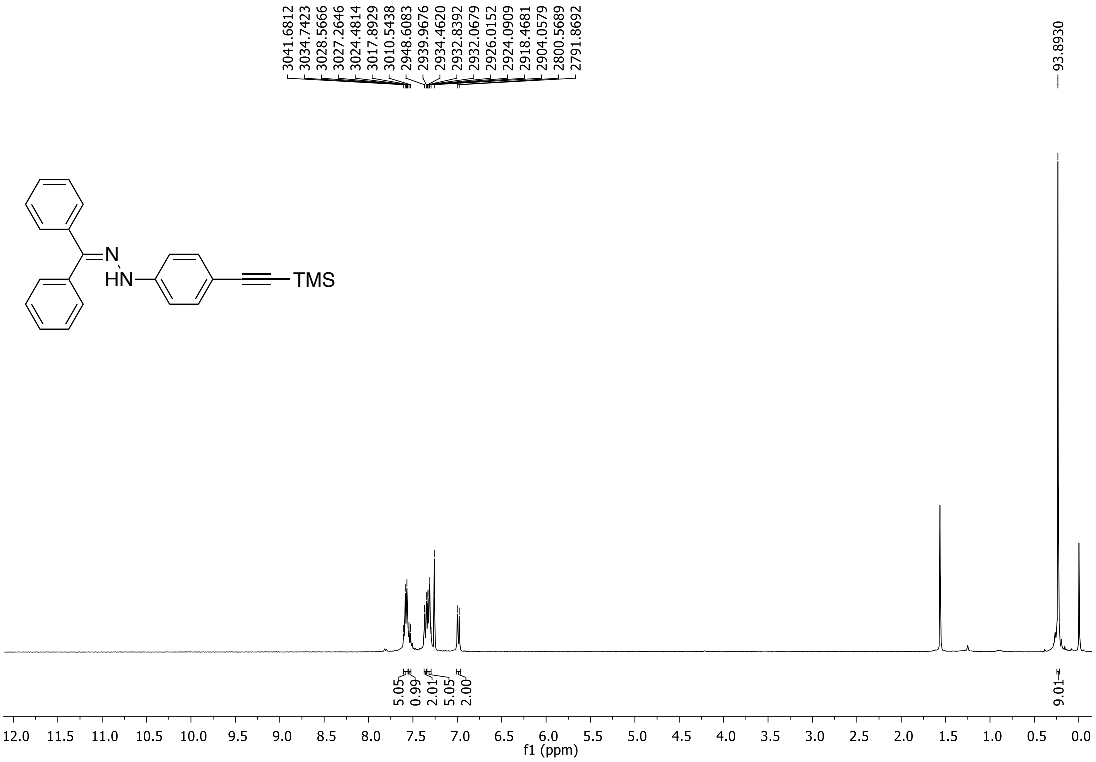

Figure S5.^{1}H NMR spectrum of **6** in $CDCl_3$ solution (400 MHz).

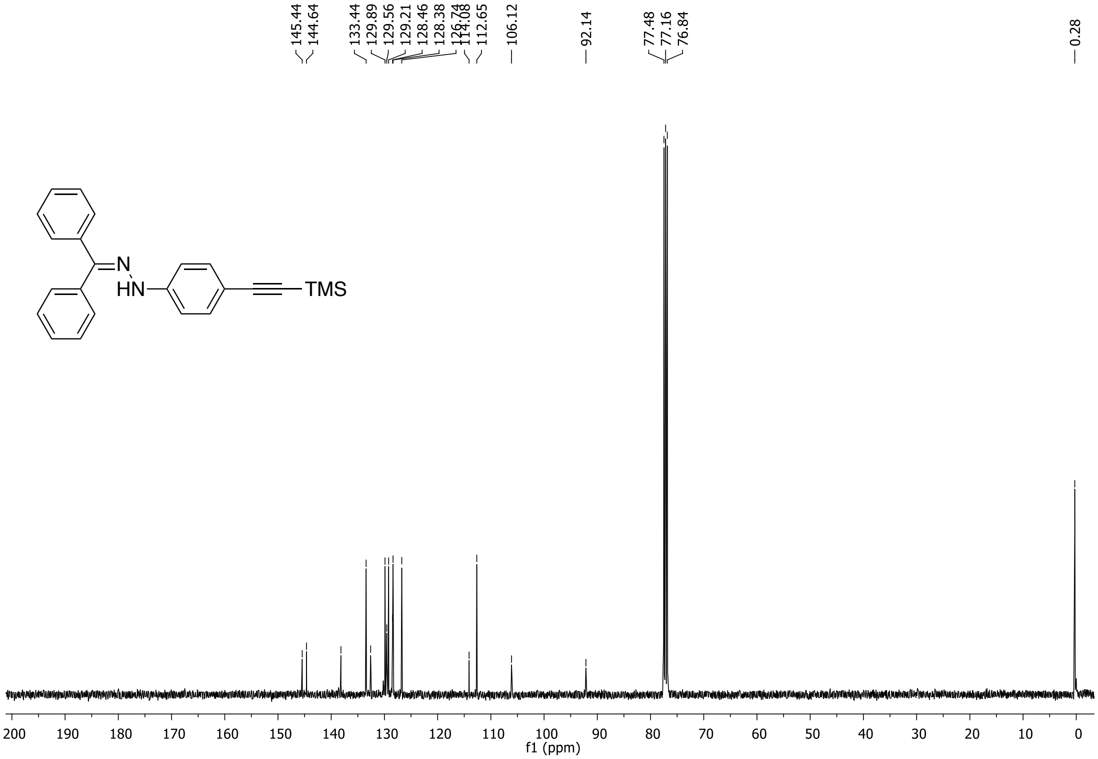

Figure S6. $^{13}C\{^1H\}$ NMR spectrum of **6** in $CDCl_3$ solution (100 MHz).

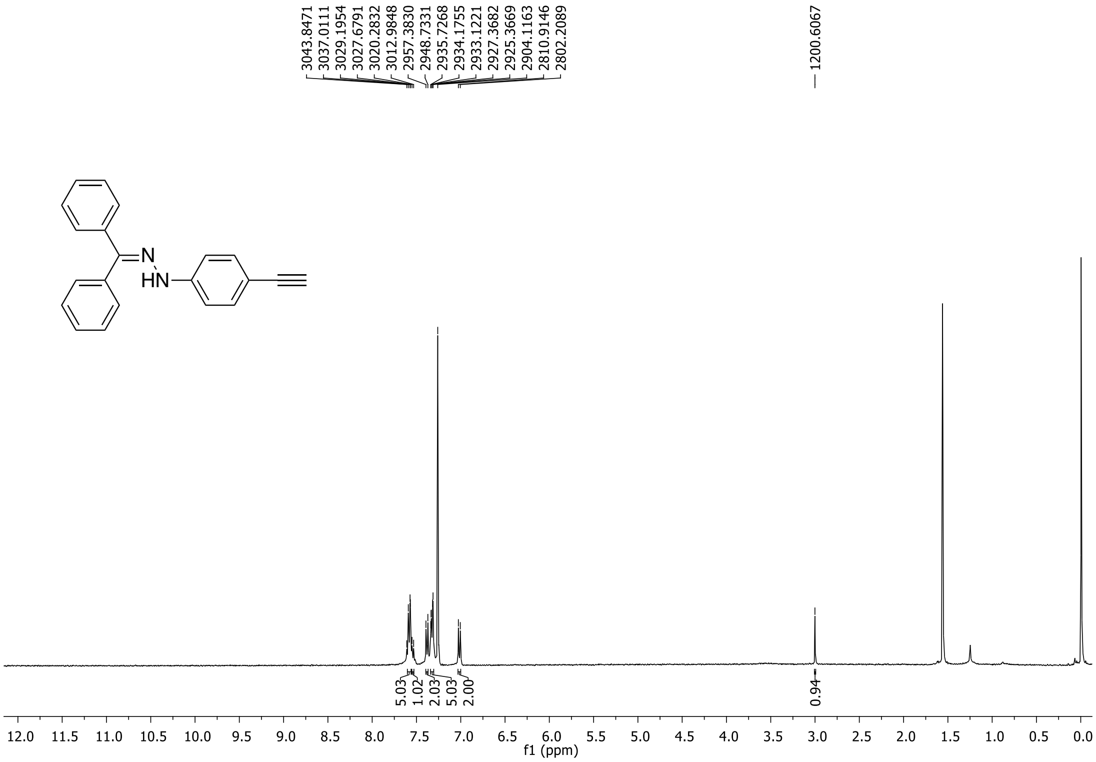

Figure S7.^{1}H NMR spectrum of **7** in $CDCl_3$ solution (400 MHz).

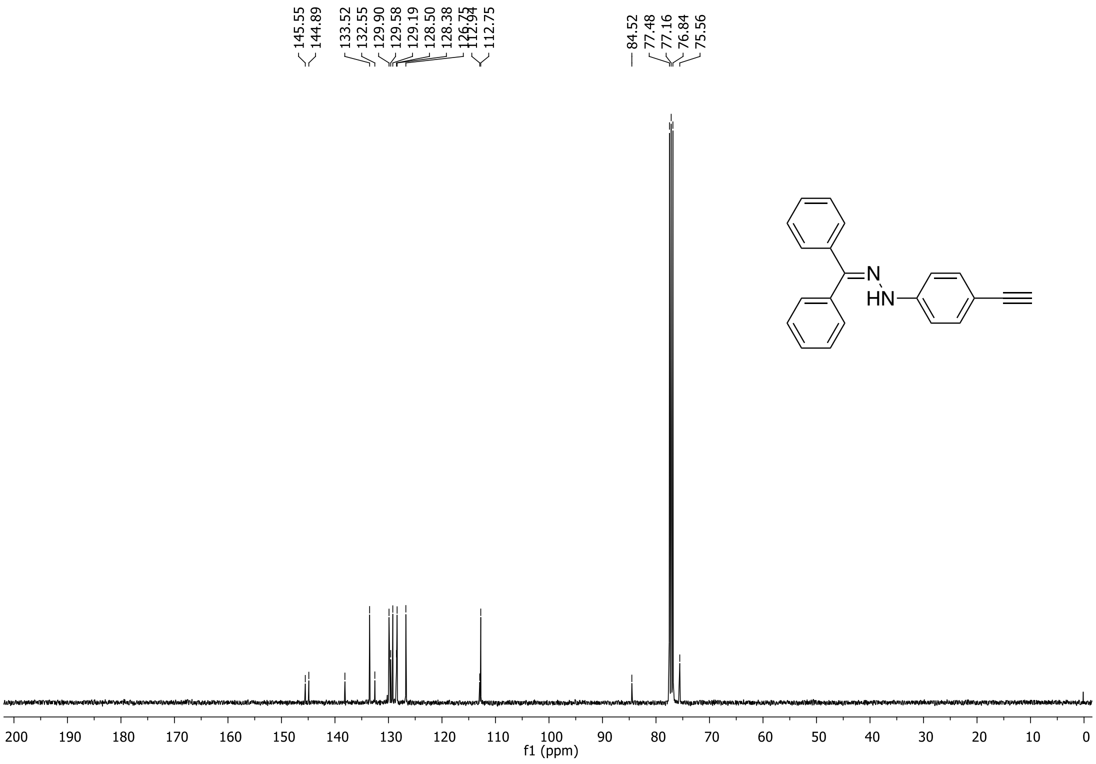

Figure S8. $^{13}C\{^1H\}$ NMR spectrum of **7** in $CDCl_3$ solution (100 MHz).

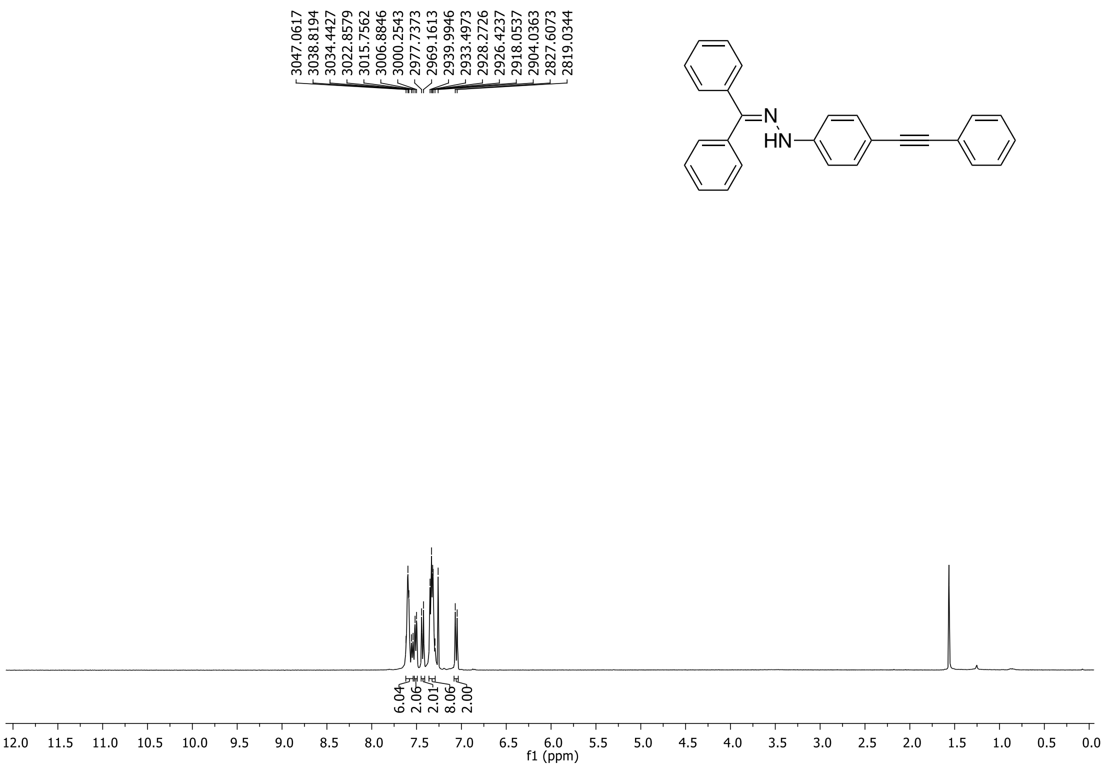

Figure S9.^{1}H NMR spectrum of **12** in $CDCl_3$ solution (400 MHz).

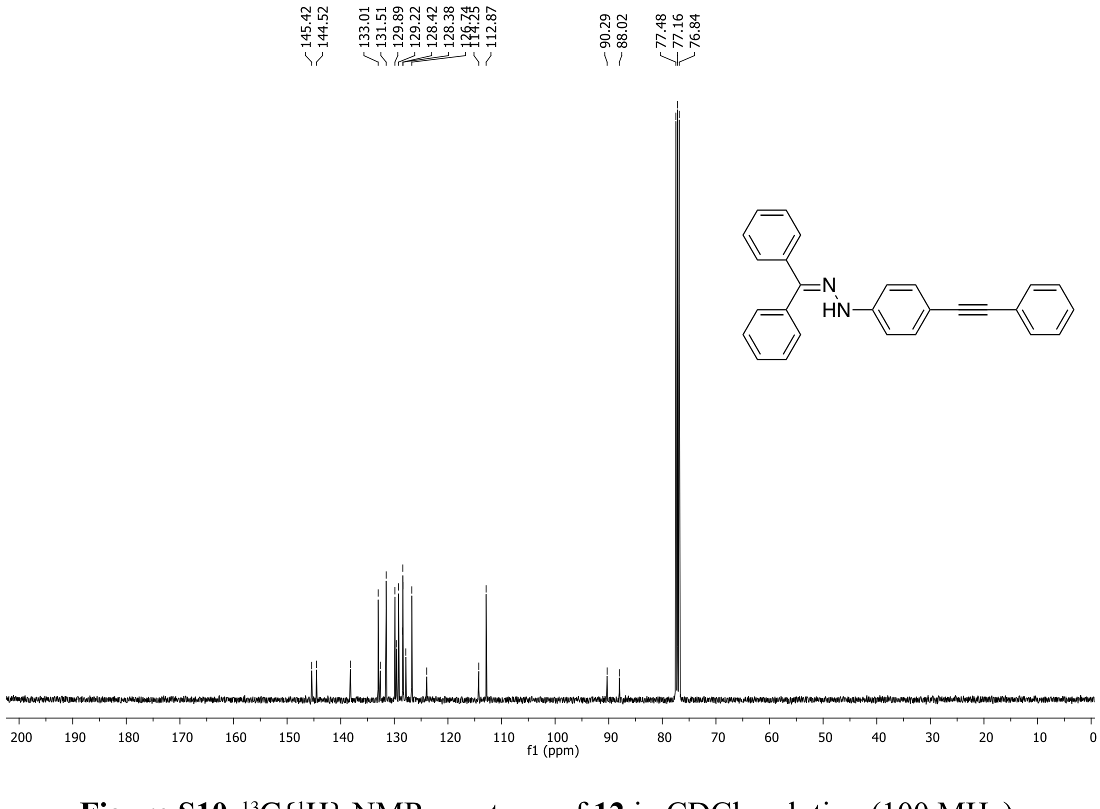

Figure S10. $^{13}C\{^1H\}$ NMR spectrum of **12** in $CDCl_3$ solution (100 MHz).

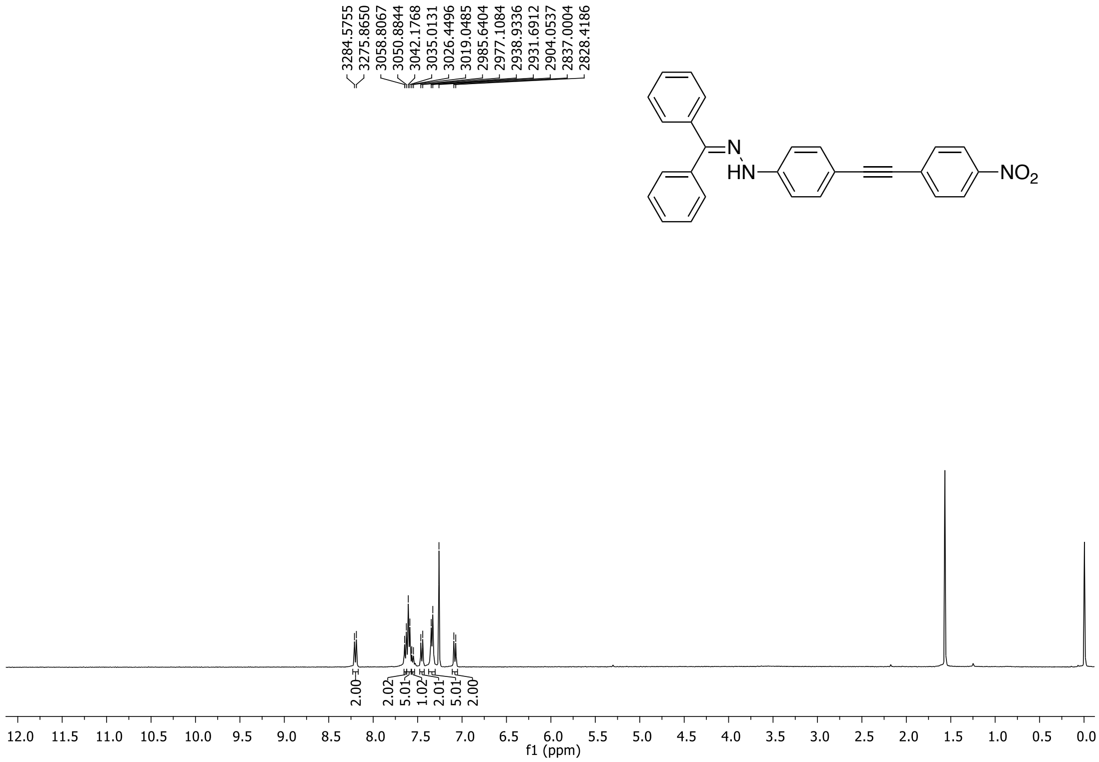

Figure S11.^{1}H NMR spectrum of **13** in $CDCl_3$ solution (400 MHz).

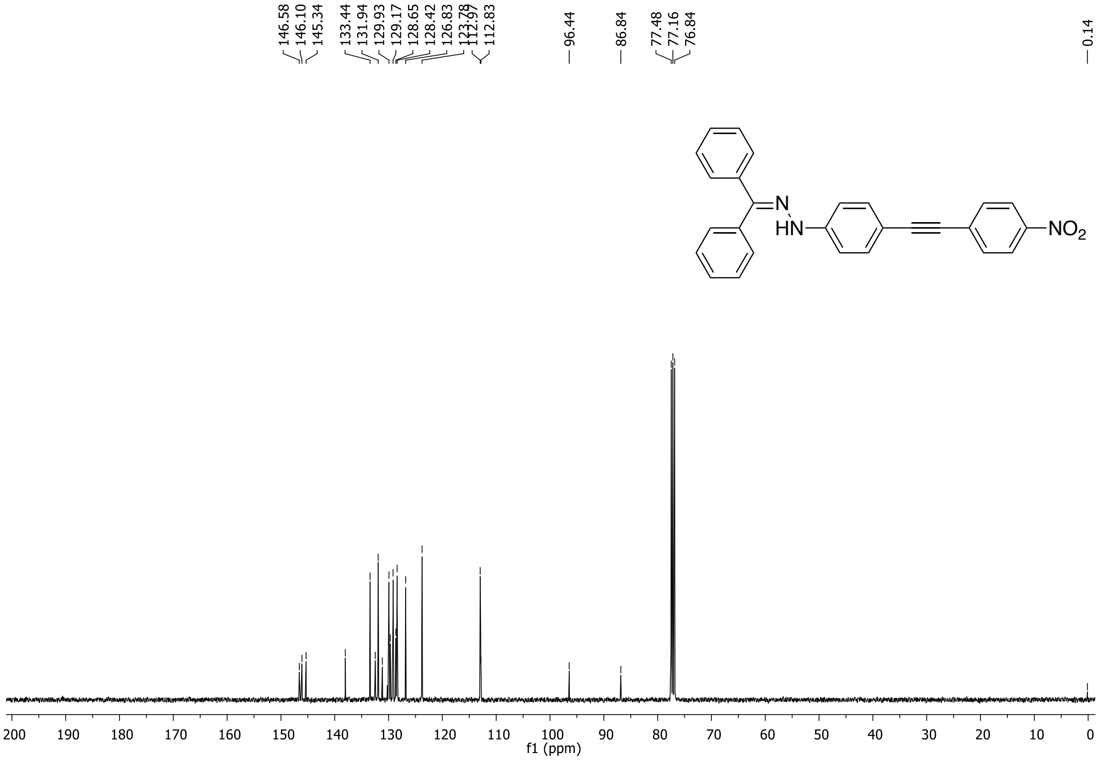

Figure S12. $^{13}C\{^{1}H\}$ NMR spectrum of **13** in $CDCl_3$ solution (100 MHz).

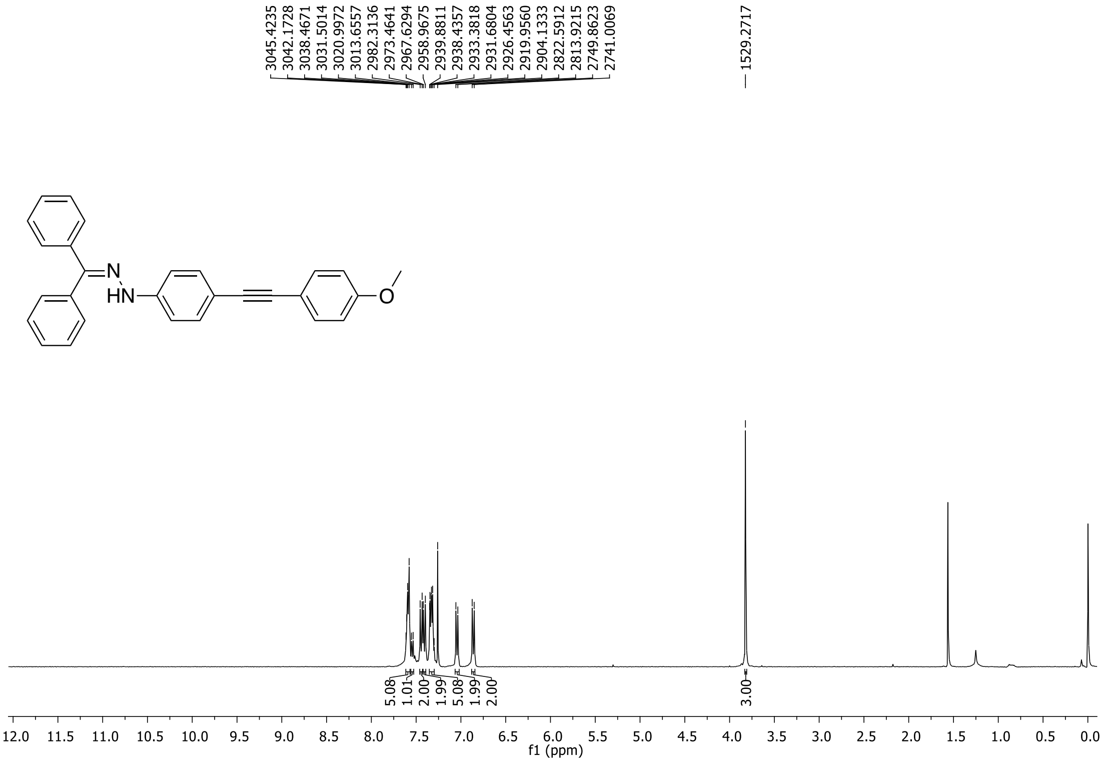

Figure S13.^{1}H NMR spectrum of **14** in $CDCl_3$ solution (400 MHz).

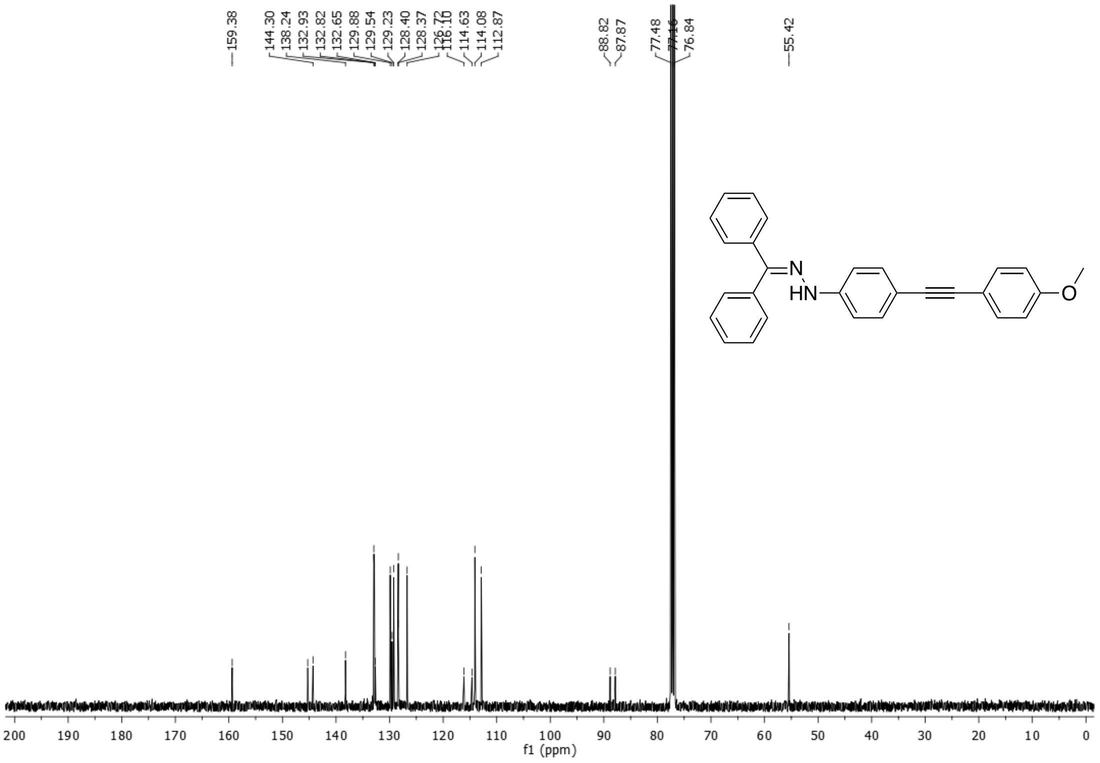

Figure S14. $^{13}C\{^1H\}$ NMR spectrum of **14** in $CDCl_3$ solution (100 MHz).

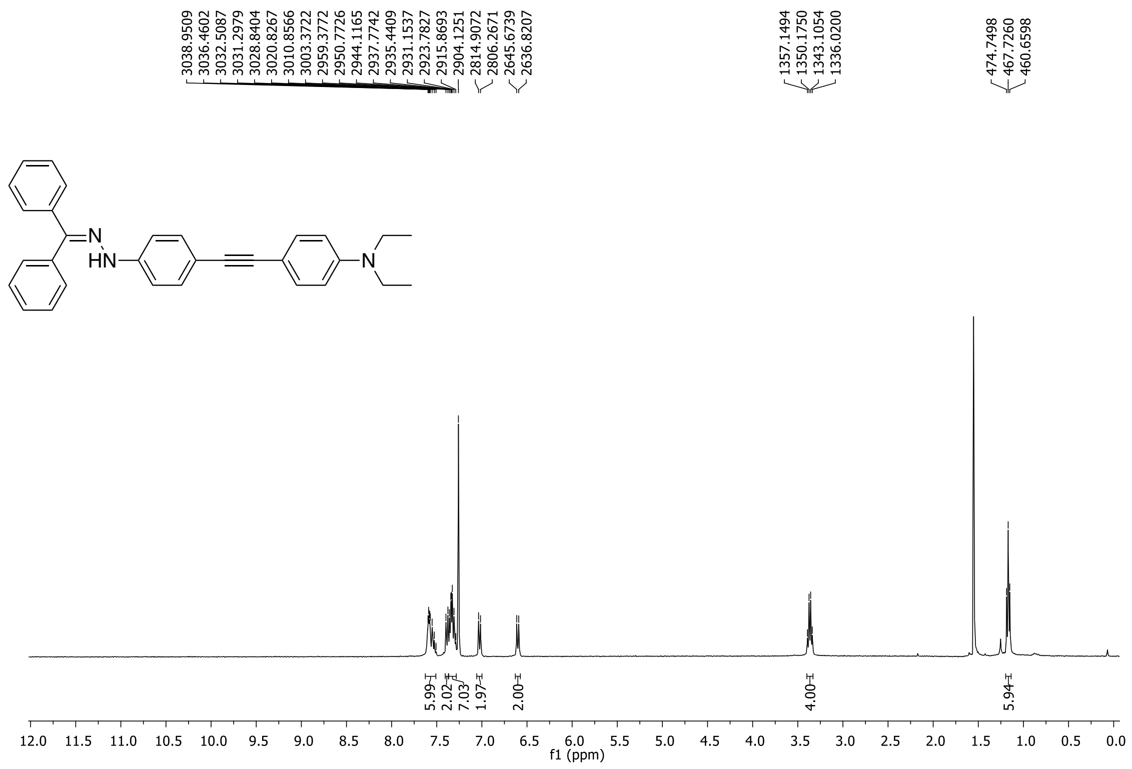

Figure S15.^{1}H NMR spectrum of **15** in $CDCl_3$ solution (400 MHz).

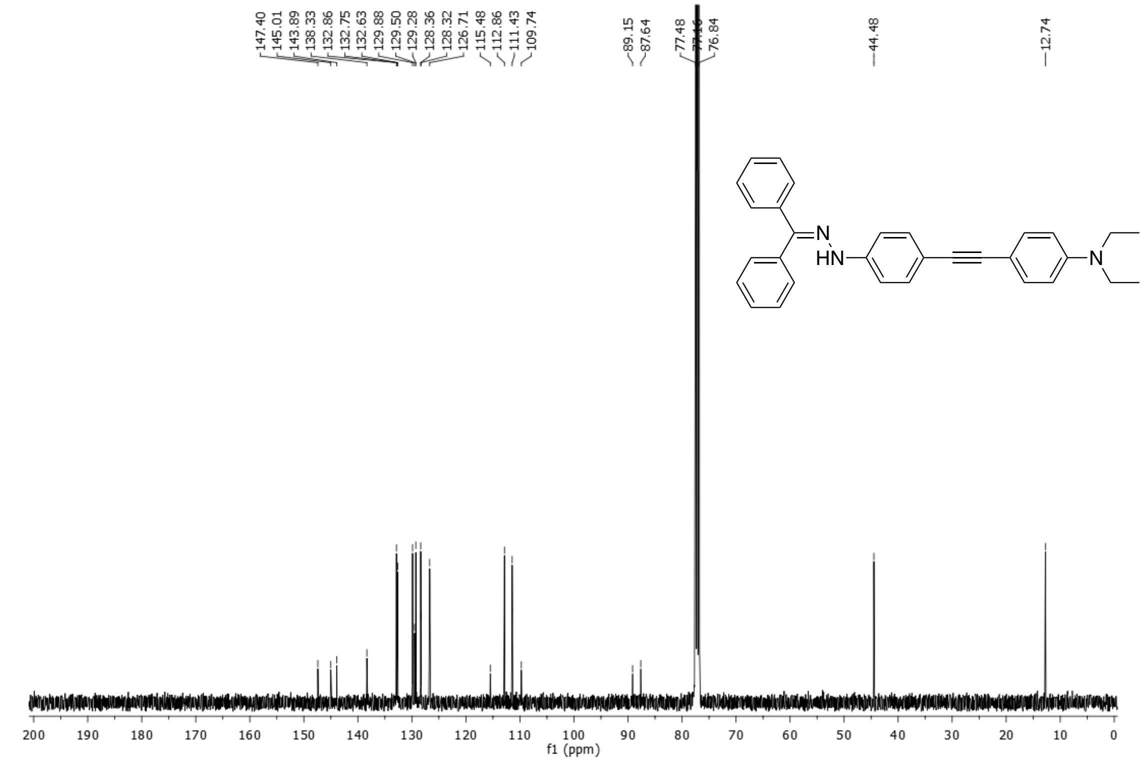

Figure S16. $^{13}C\{^1H\}$ NMR spectrum of **15** in $CDCl_3$ solution (100 MHz).

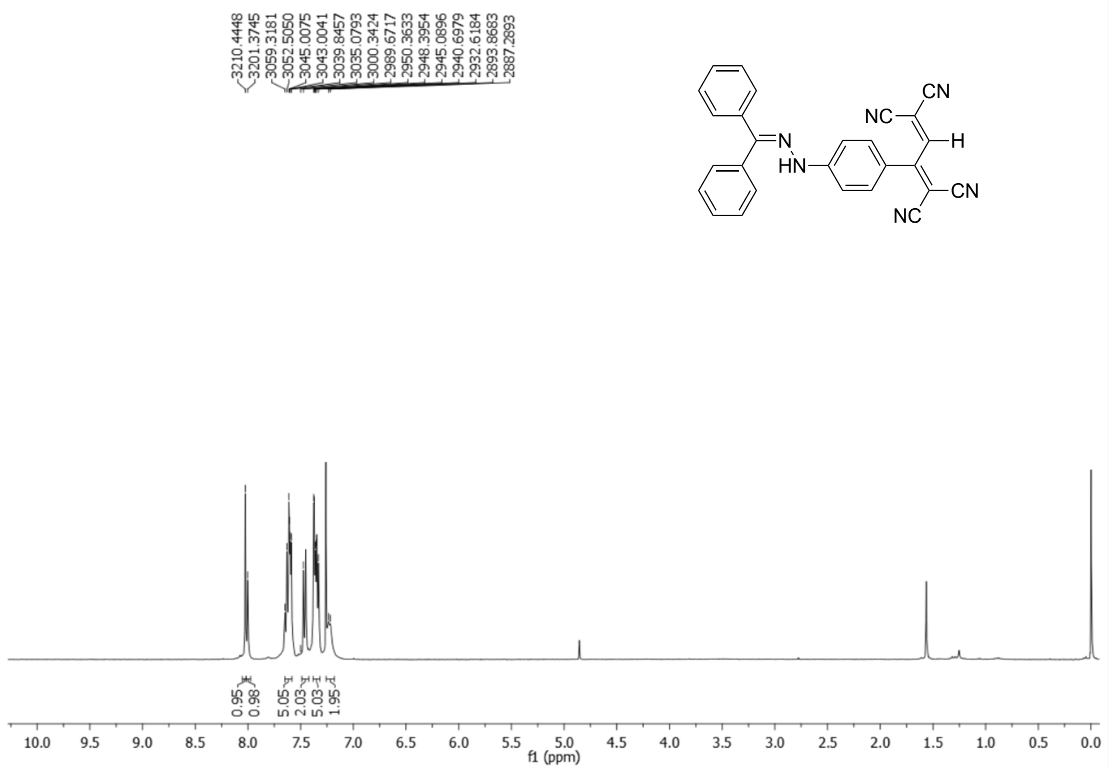

Figure S17.^{1}H NMR spectrum of **17** in $CDCl_3$ solution (400 MHz).

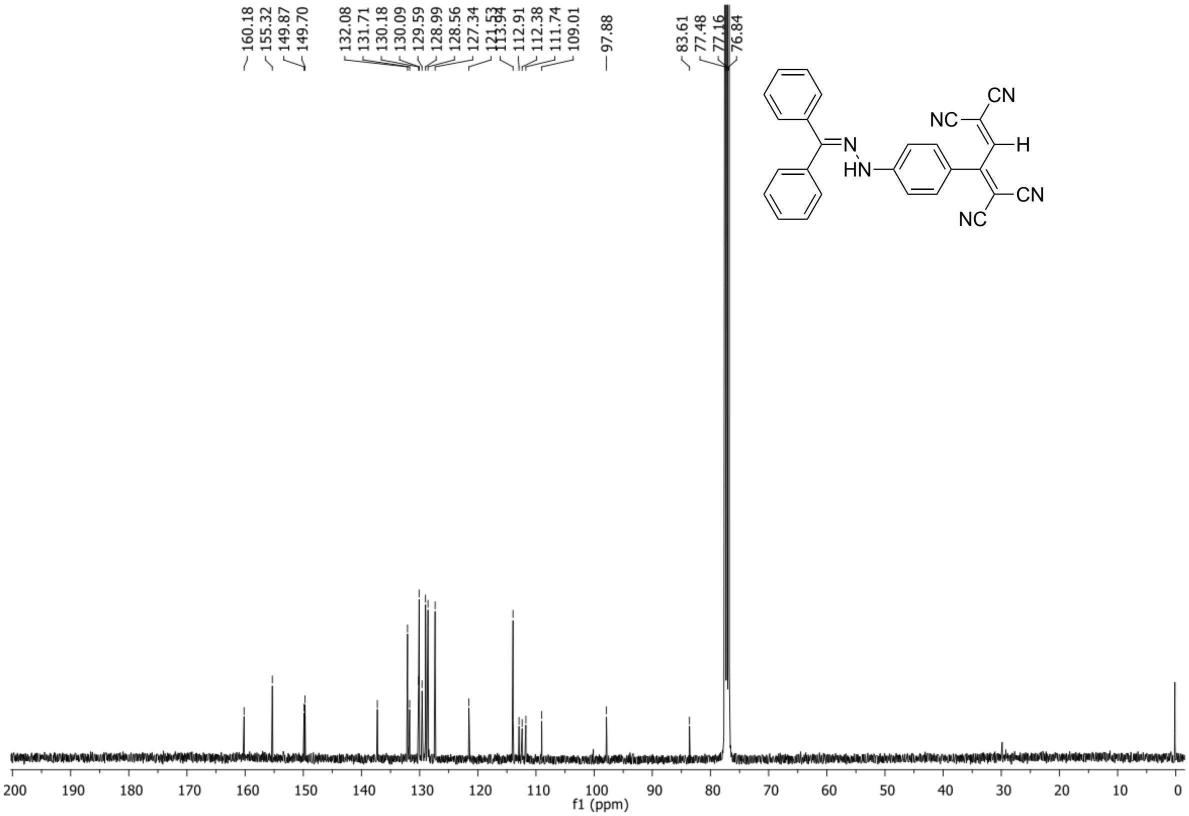

Figure S18. $^{13}C\{^{1}H\}$ NMR spectrum of **17** in $CDCl_3$ solution (100 MHz).

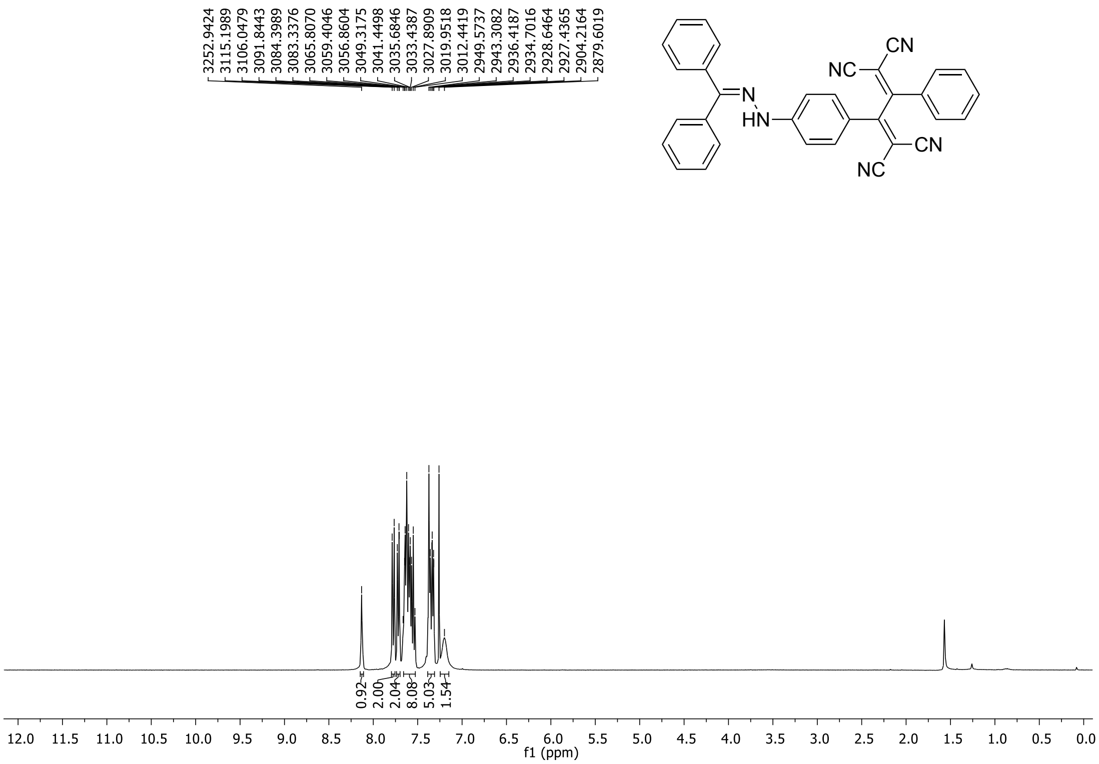

Figure S19.^{1}H NMR spectrum of **18** in $CDCl_3$ solution (400 MHz).

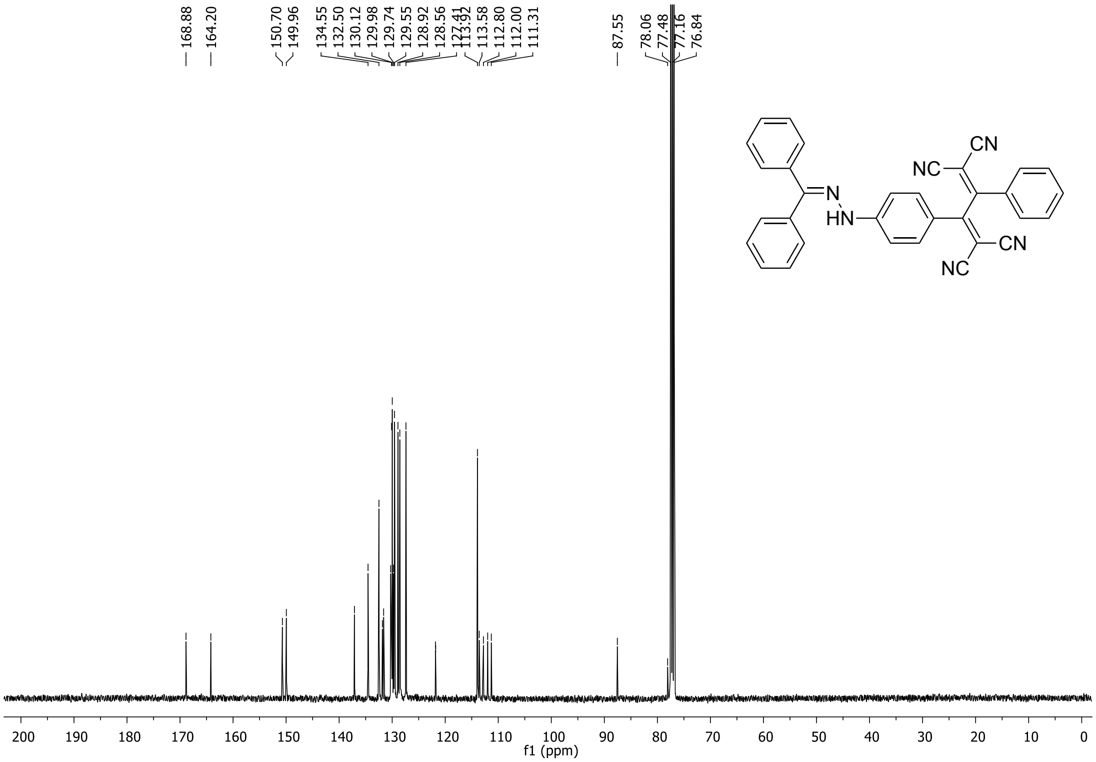

Figure S20. $^{13}C\{^1H\}$ NMR spectrum of **18** in $CDCl_3$ solution (100 MHz).

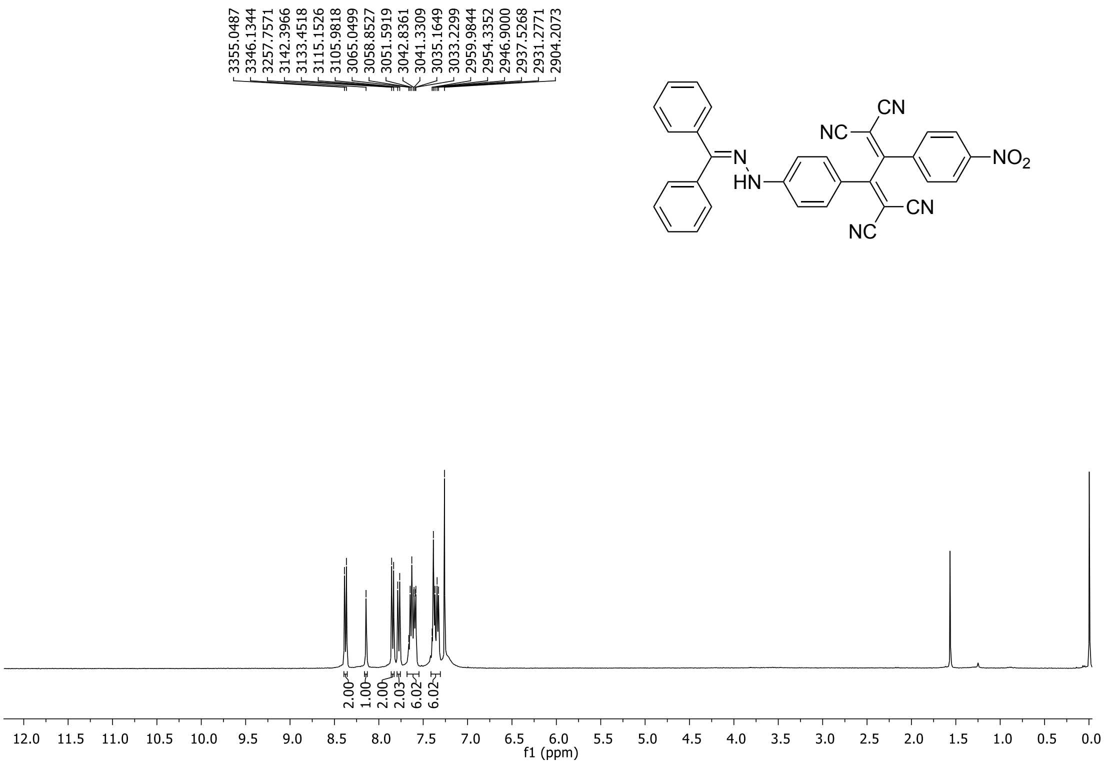

Figure S21.^{1}H NMR spectrum of **19** in $CDCl_3$ solution (400 MHz).

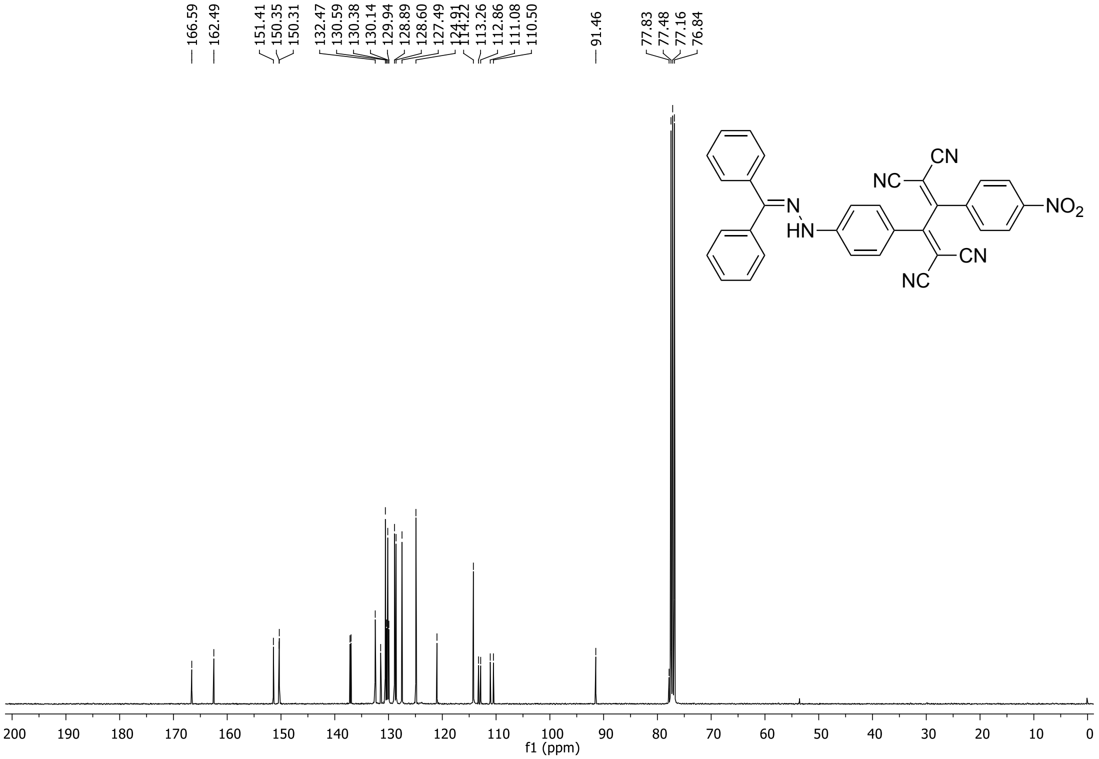

Figure S22. $^{13}C\{^{1}H\}$ NMR spectrum of **19** in $CDCl_3$ solution (100 MHz).

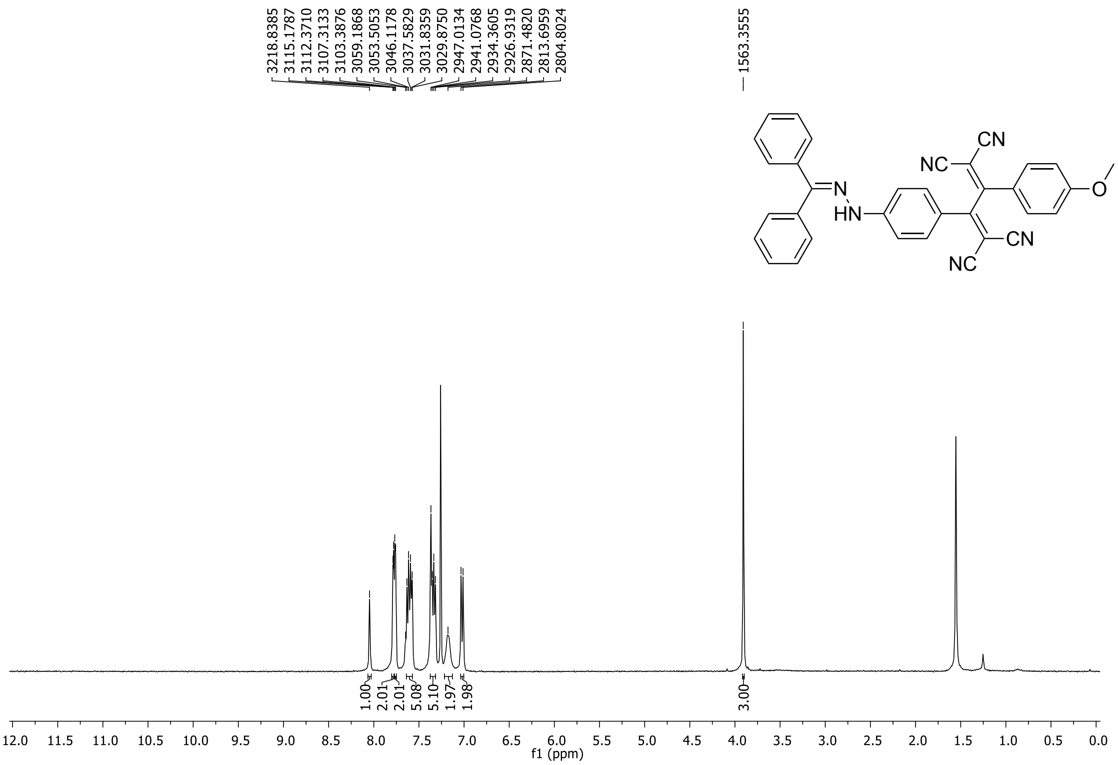

Figure S23. ^{1}H NMR spectrum of **20** in $CDCl_3$ solution (400 MHz).

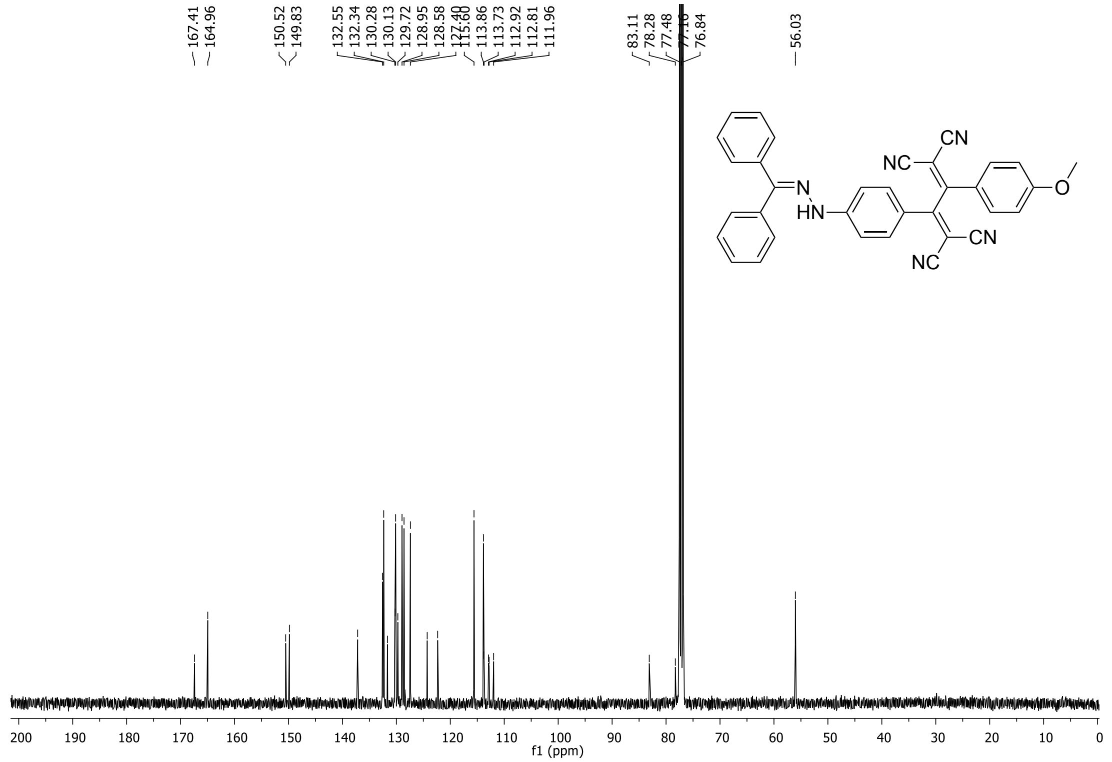

Figure S24. $^{13}C\{^{1}H\}$ NMR spectrum of **20** in $CDCl_3$ solution (100 MHz).

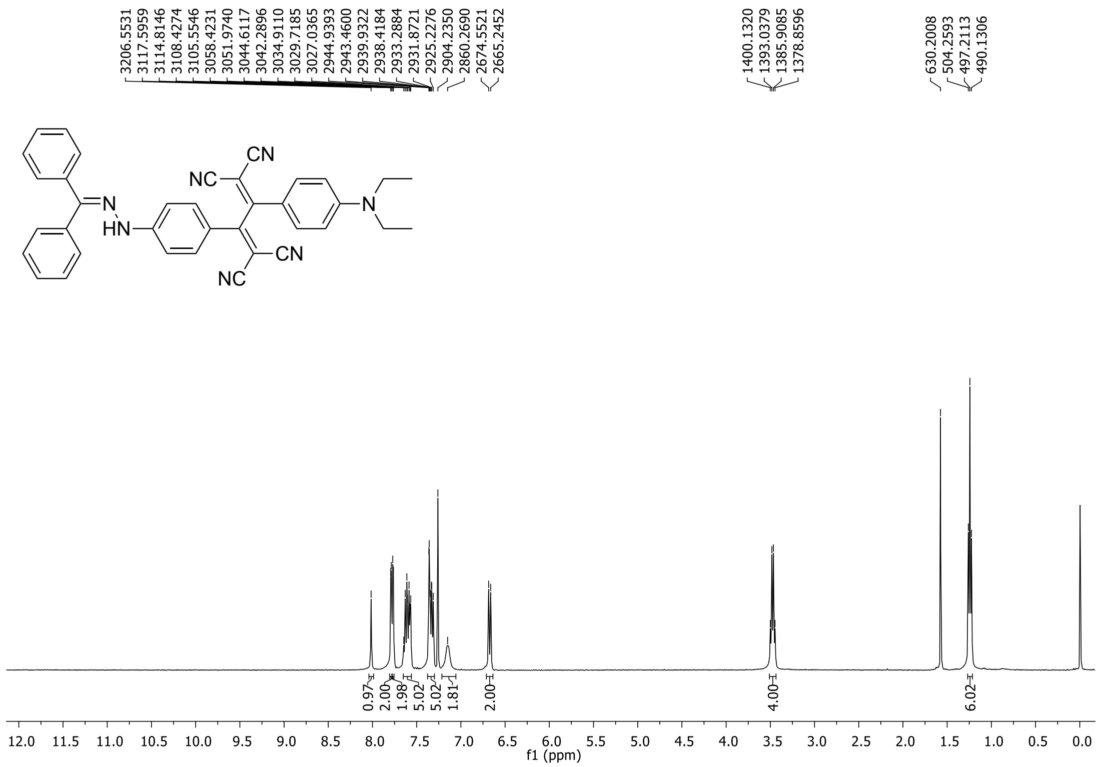

Figure S25.^{1}H NMR spectrum of **21** in $CDCl_3$ solution (400 MHz).

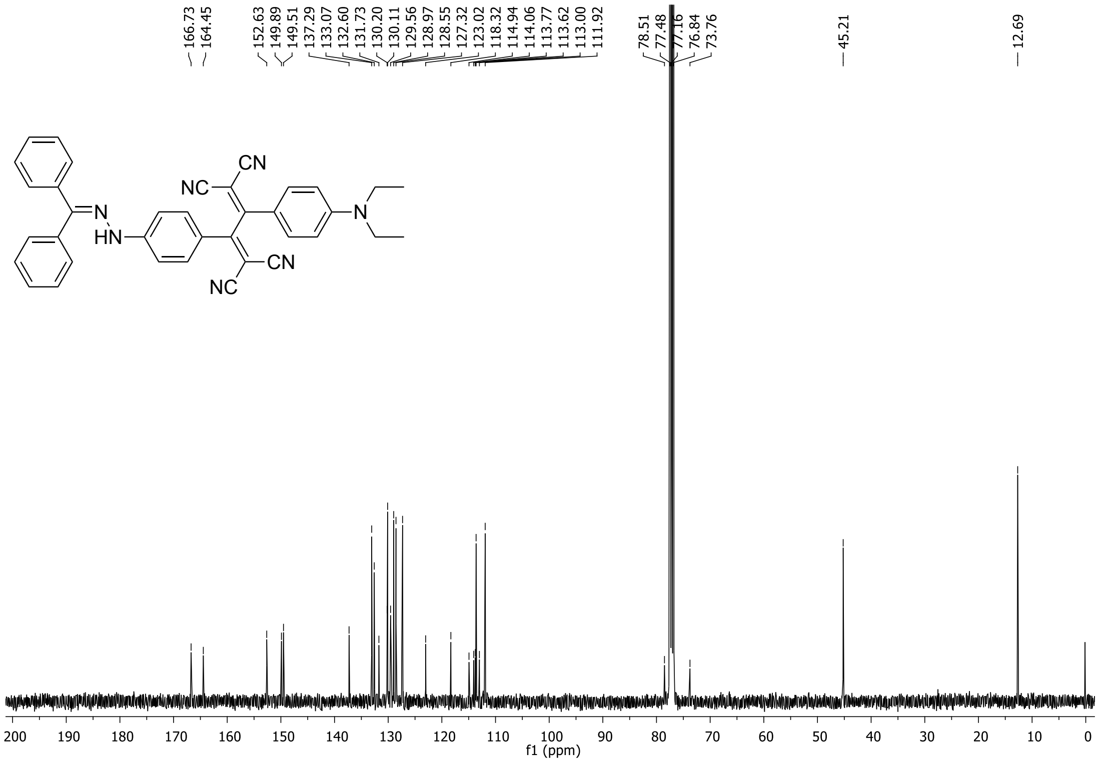

Figure S26. $^{13}C\{^{1}H\}$ NMR spectrum of **21** in $CDCl_3$ solution (100 MHz).

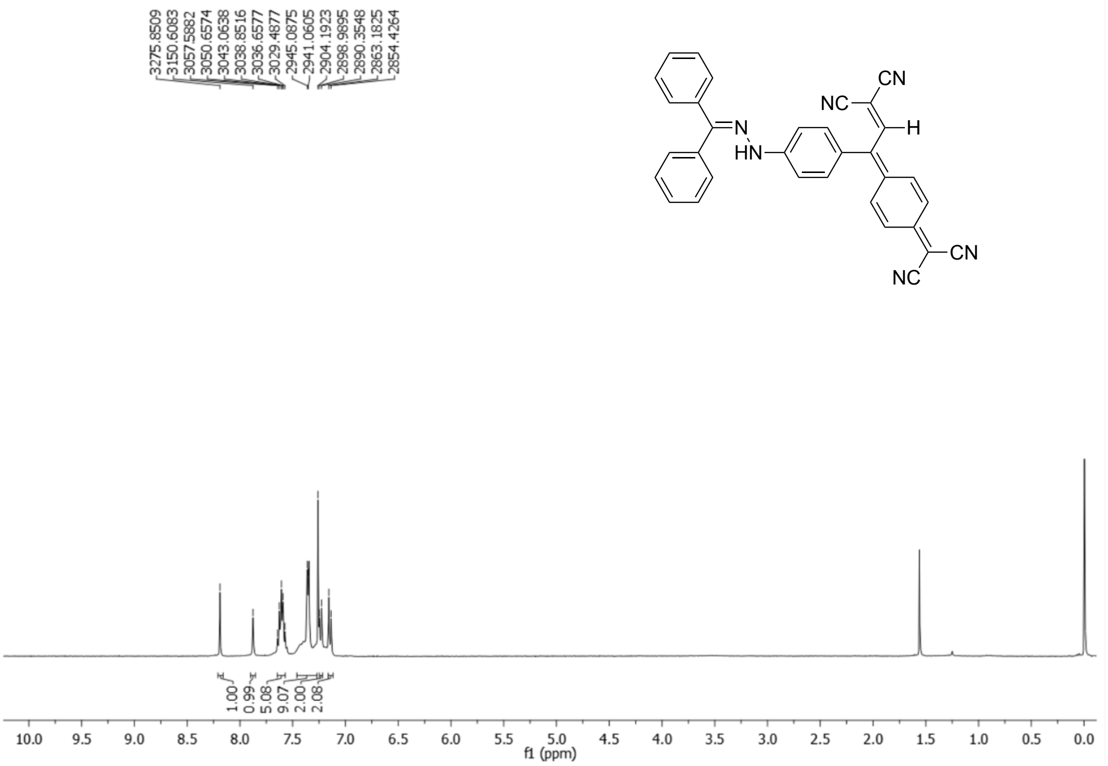

Figure S27.^{1}H NMR spectrum of **23** in $CDCl_3$ solution (400 MHz).

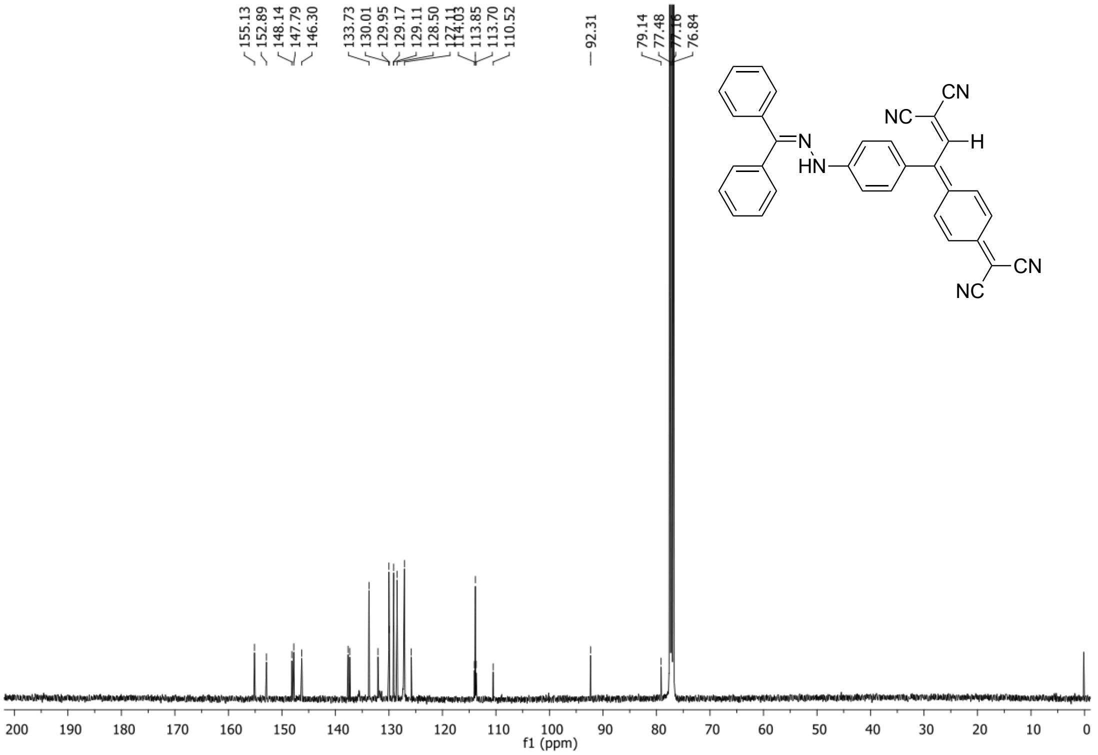

Figure S28. $^{13}C\{^1H\}$ NMR spectrum of **23** in $CDCl_3$ solution (100 MHz).

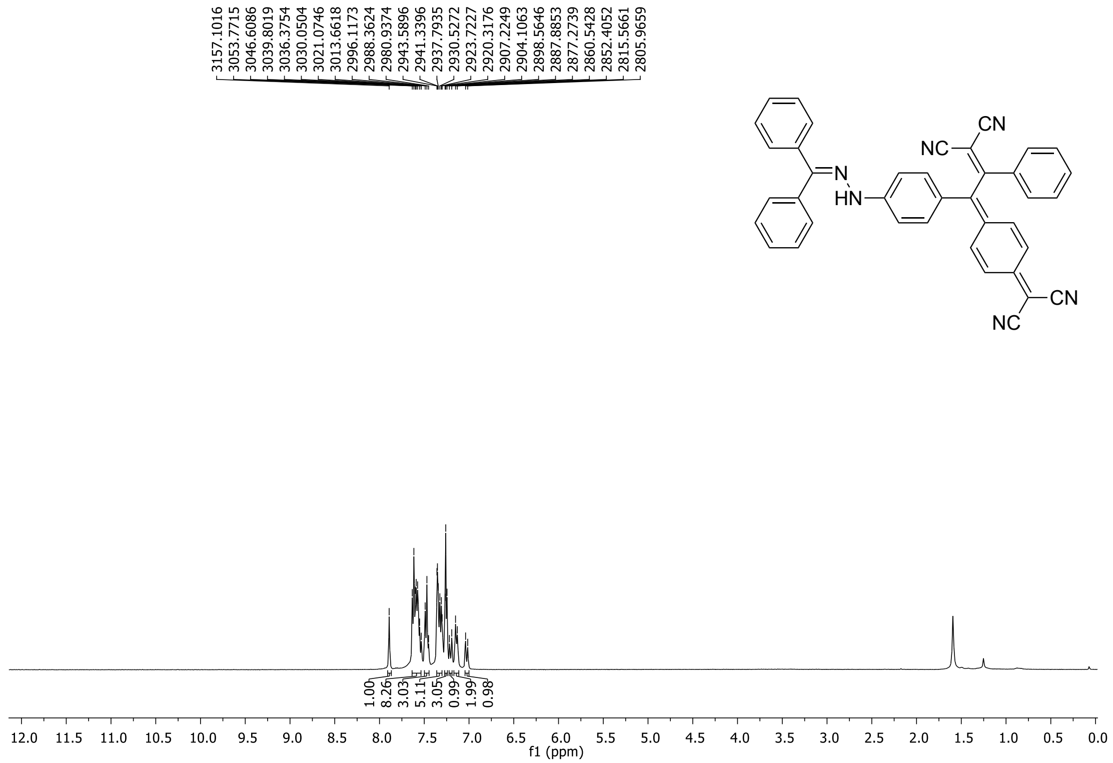

Figure S29.^{1}H NMR spectrum of **24** in $CDCl_3$ solution (400 MHz).

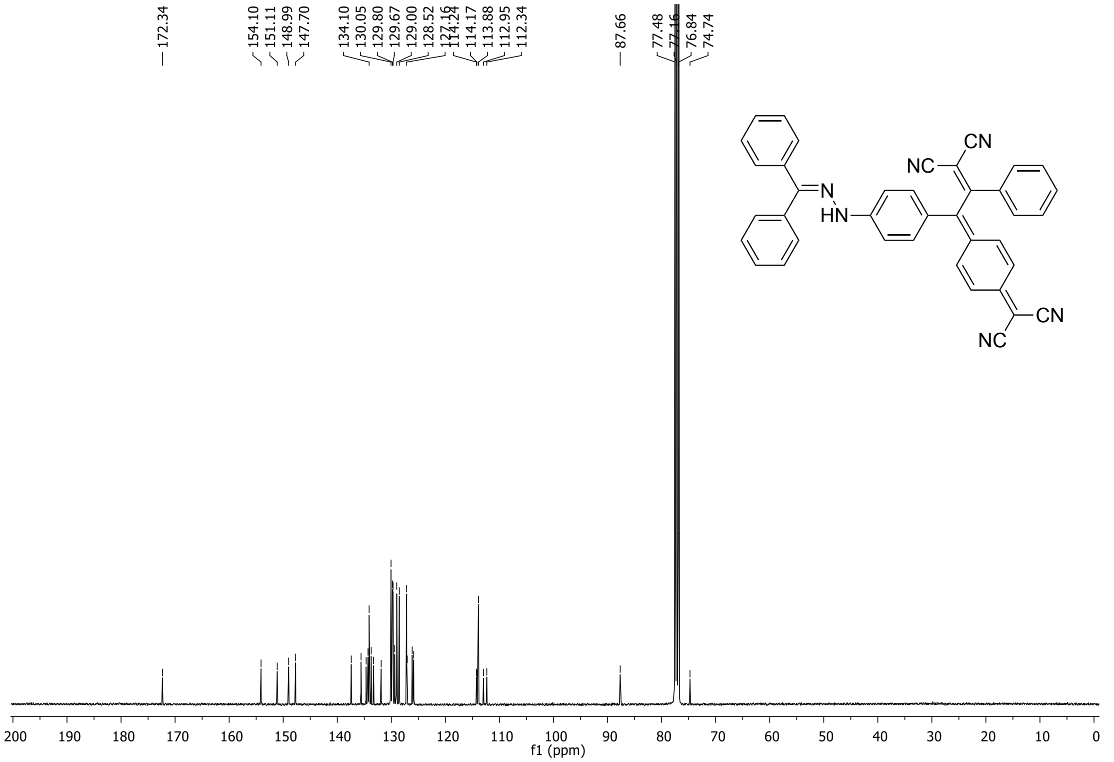

Figure S30. $^{13}C\{^{1}H\}$ NMR spectrum of **24** in $CDCl_3$ solution (100 MHz).

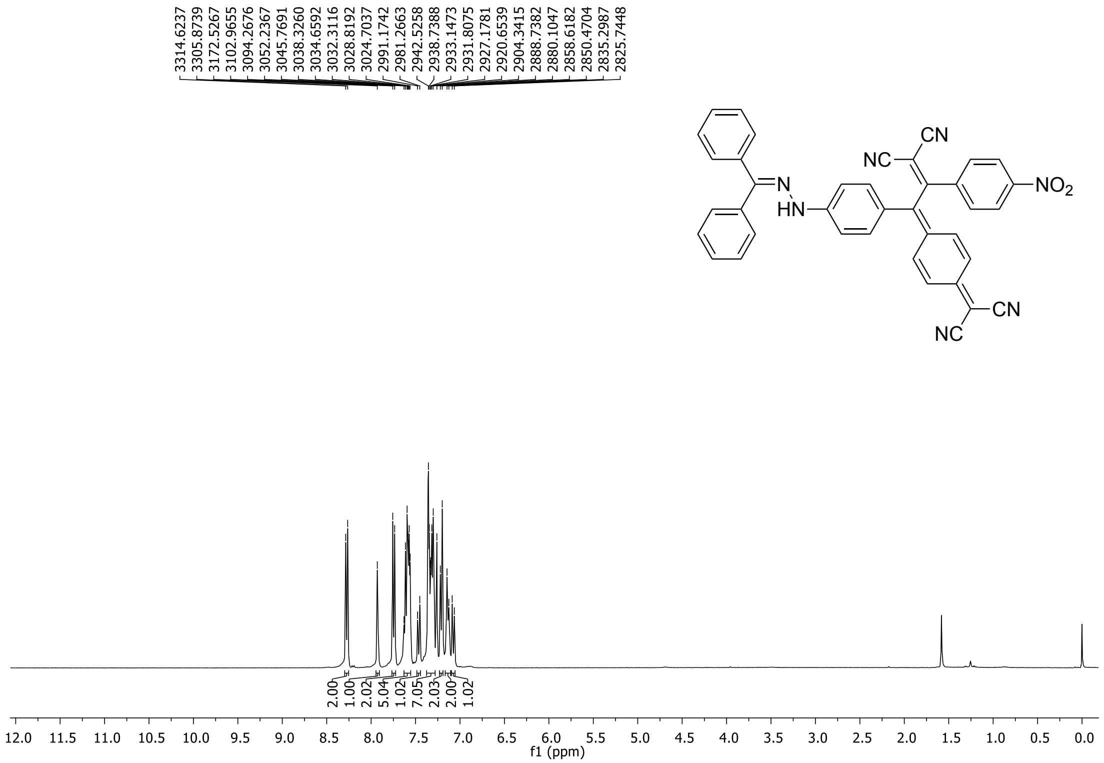

Figure S31.^{1}H NMR spectrum of **25** in $CDCl_3$ solution (400 MHz).

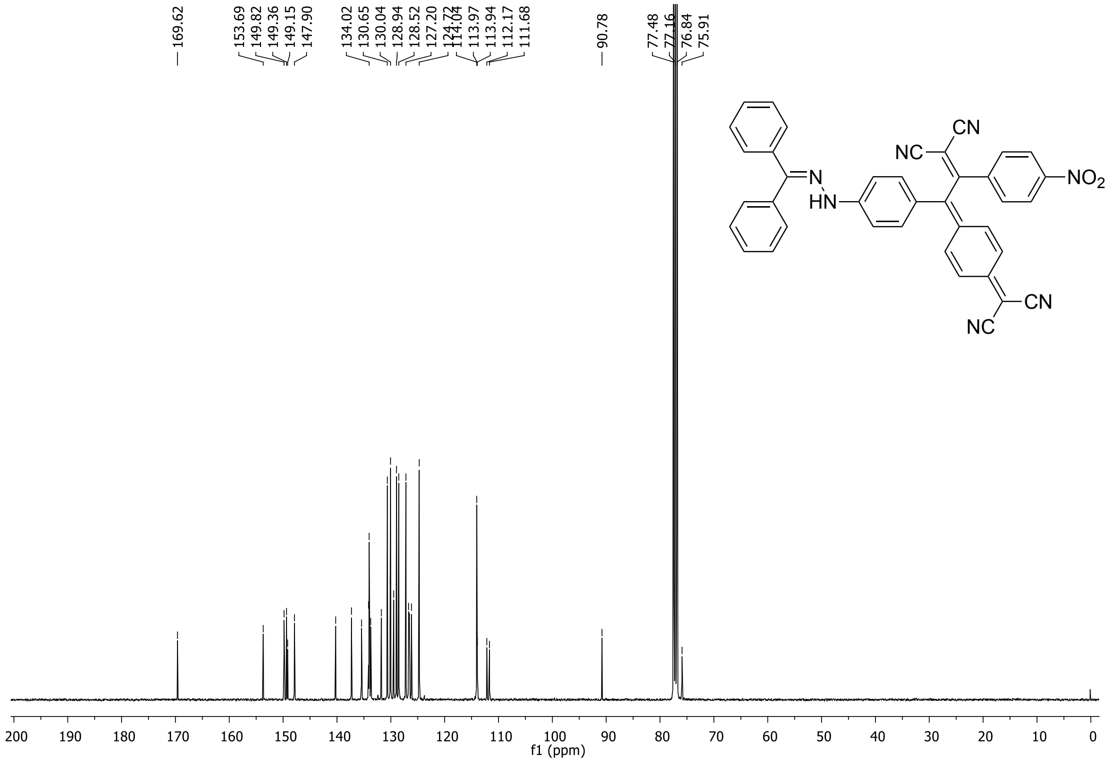

Figure S32. $^{13}C\{^1H\}$ NMR spectrum of **25** in $CDCl_3$ solution (100 MHz).

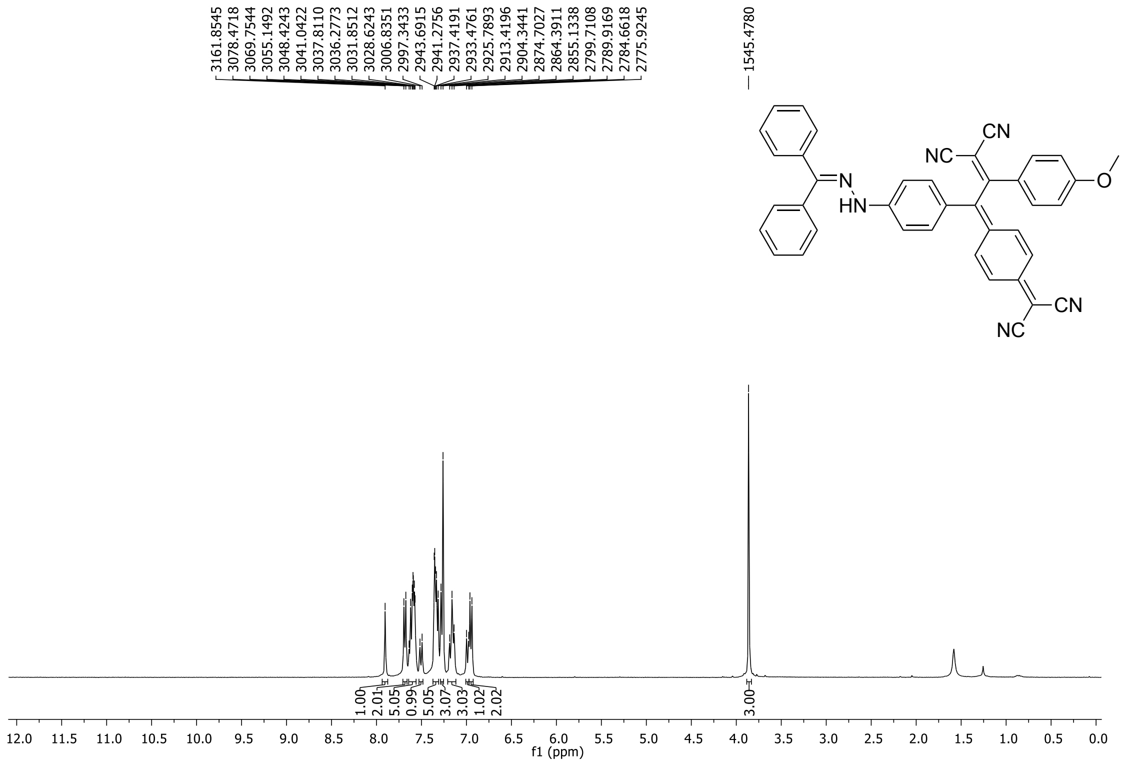

Figure S33.^{1}H NMR spectrum of **26** in $CDCl_3$ solution (400 MHz).

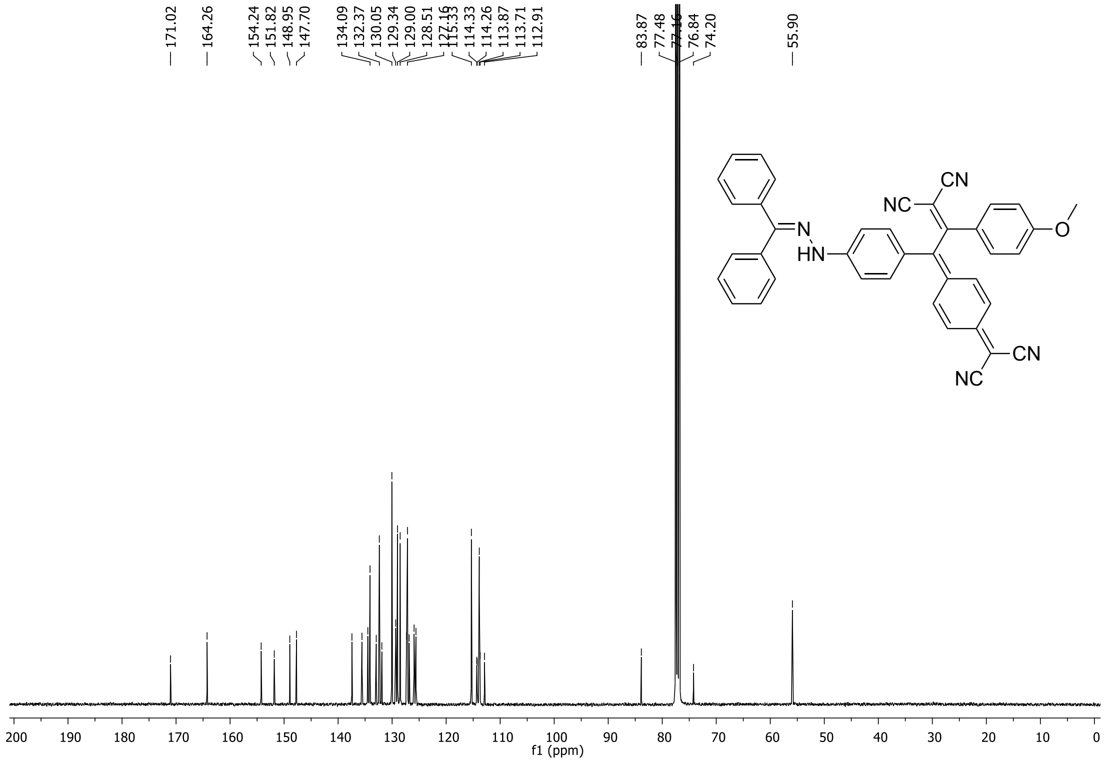

Figure S34. $^{13}C\{^1H\}$ NMR spectrum of **26** in $CDCl_3$ solution (100 MHz).

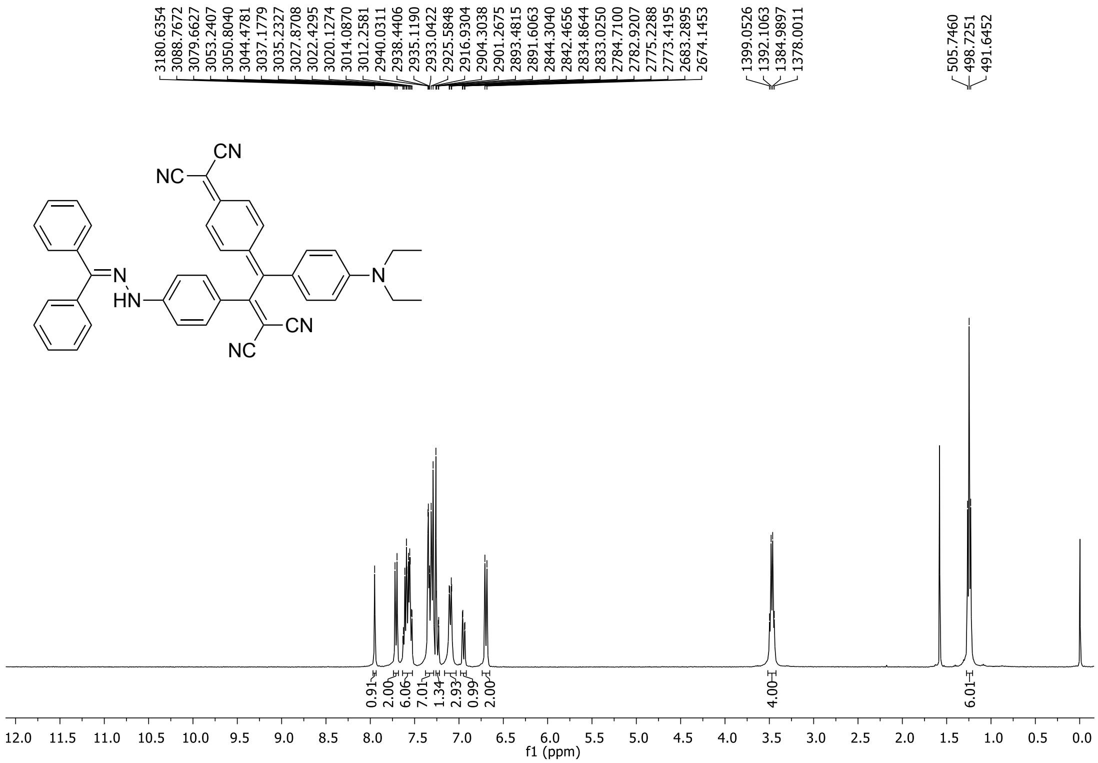

Figure S35.^{1}H NMR spectrum of **27** in $CDCl_3$ solution (400 MHz).

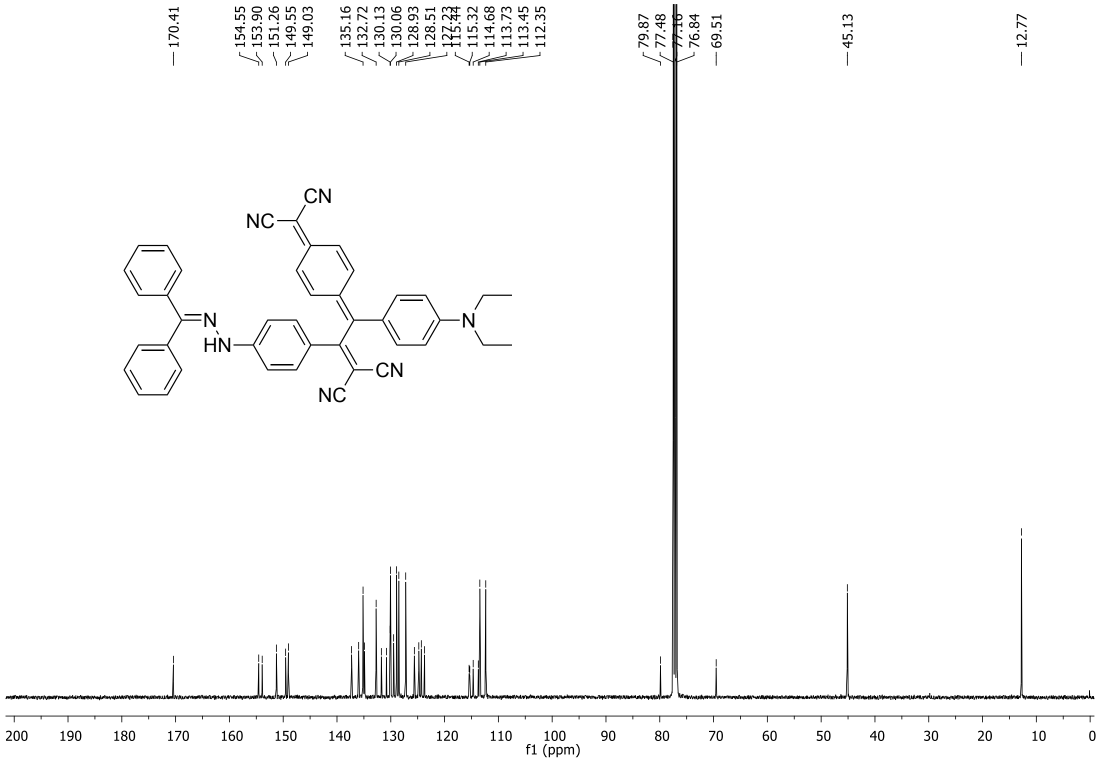

Figure S36. $^{13}C\{^1H\}$ NMR spectrum of **27** in $CDCl_3$ solution (100 MHz).

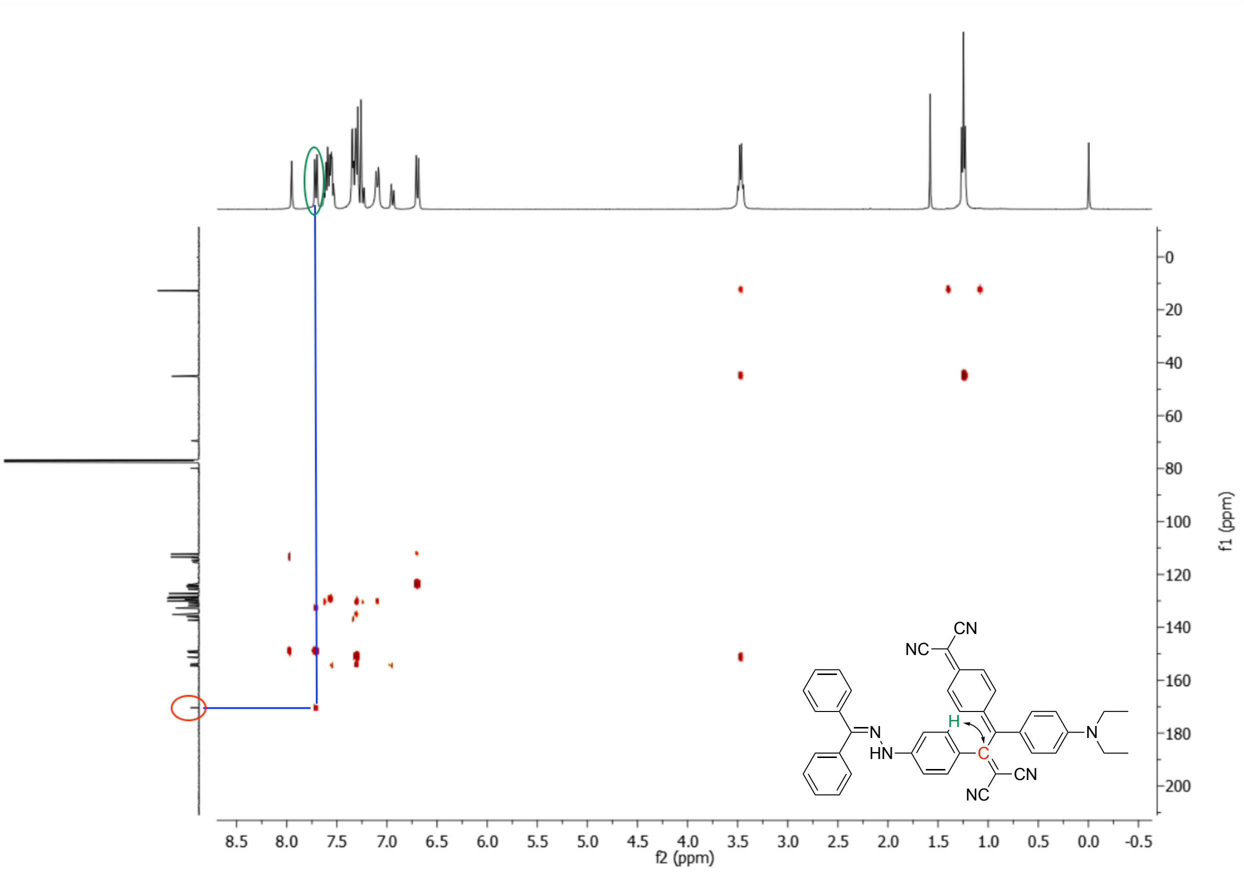

Figure S37. HMBC spectrum of **27** in $CDCl_3$ solution.

2. High-Resolution Mass Spectrometry (HR-MS) Data

Elemental Composition Report Page 1

Single Mass Analysis
Tolerance = 10000.0 PPM / DBE: min = -5.5, max = 1000.0
Element prediction: Off
Number of isotope peaks used for i-FIT = 9

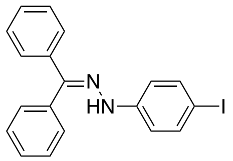

Monoisotopic Mass, Even Electron Ions
2 formula(e) evaluated with 1 results within limits (all results (up to 1000) for each mass)
Elements Used:
C: 19-19 H: 15-16 N: 2-2 I: 1-3

Kubra Erden
42213_20240109_04-04 22 (0.863) Cm (1:25)

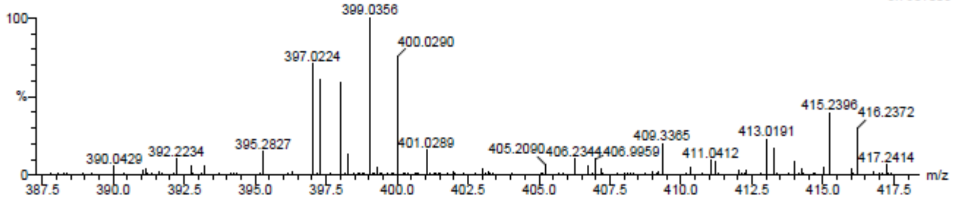

Minimum:				-5.5			
Maximum:		1000.0	10000.0	1000.0			
Mass	Calc. Mass	mDa	PPM	DBE	i-FIT	i-FIT (Norm)	Formula
399.0356	399.0358	-0.2	-0.5	12.5	935.3	0.0	C19 H16 N2 I

Figure S38. HR-MS spectrum of **4**.

Elemental Composition Report Page 1

Single Mass Analysis
Tolerance = 1000.0 PPM / DBE: min = -5.5, max = 1000.0
Element prediction: Off
Number of isotope peaks used for i-FIT = 9

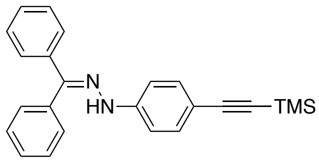

Monoisotopic Mass, Even Electron Ions
7 formula(e) evaluated with 1 results within limits (all results (up to 1000) for each mass)
Elements Used:
C: 24-24 H: 24-26 N: 2-2 Si: 1-8

Kubra Erden
42871_20240312_01-11 17 (0.673) Cm (14:17)

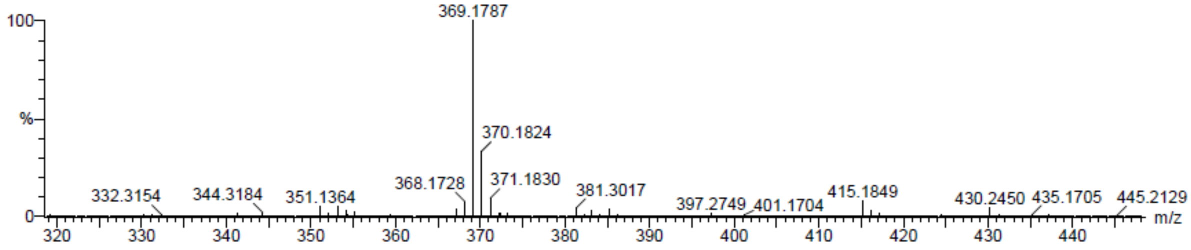

Minimum:				-5.5			
Maximum:		1000.0	1000.0	1000.0			
Mass	Calc. Mass	mDa	PPM	DBE	i-FIT	i-FIT (Norm)	Formula
369.1787	369.1787	0.0	0.0	14.5	771.6	0.0	C24 H25 N2 Si

Figure S39. HR-MS spectrum of **6**.

Elemental Composition Report | **Page 1**

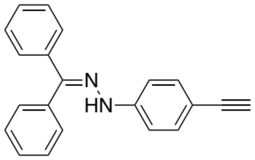

Single Mass Analysis
Tolerance = 1000.0 PPM / DBE: min = -5.5, max = 1000.0
Element prediction: Off
Number of isotope peaks used for i-FIT = 9

Monoisotopic Mass, Even Electron Ions
1 formula(e) evaluated with 1 results within limits (all results (up to 1000) for each mass)
Elements Used:
C: 21-21 H: 16-17 N: 1-3
Kubra Erden
42392_20240202_01-04 10 (0.396) Cm (1:12)

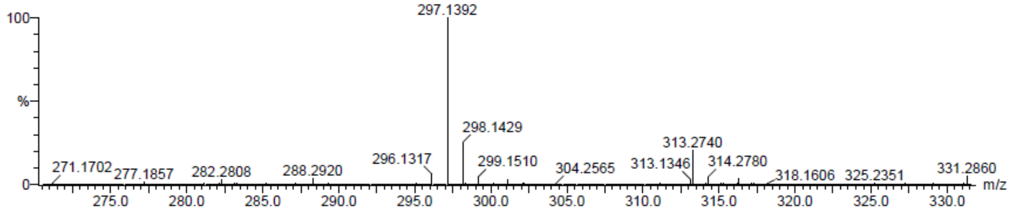

Minimum:				-5.5			
Maximum:		1000.0	1000.0	1000.0			
Mass	Calc. Mass	mDa	PPM	DBE	i-FIT	i-FIT (Norm)	Formula
297.1392	297.1392	0.0	0.0	14.5	793.0	0.0	C21 H17 N2

Figure S40. HR-MS spectrum of **7**.

Elemental Composition Report | **Page 1**

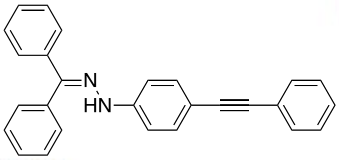

Single Mass Analysis
Tolerance = 10000.0 PPM / DBE: min = -5.5, max = 1000.0
Element prediction: Off
Number of isotope peaks used for i-FIT = 9

Monoisotopic Mass, Even Electron Ions
1 formula(e) evaluated with 1 results within limits (all results (up to 1000) for each mass)
Elements Used:
C: 27-27 H: 20-21 N: 2-2
Kübra Erden
40008_20230518_01-02 12 (0.484) Cm (11:22)

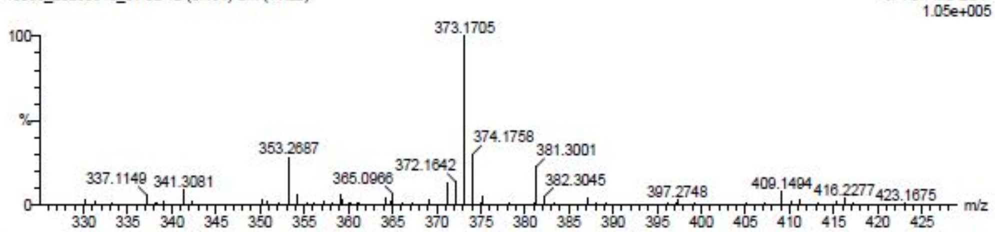

Minimum:				-5.5			
Maximum:		1000.0	10000.0	1000.0			
Mass	Calc. Mass	mDa	PPM	DBE	i-FIT	i-FIT (Norm)	Formula
373.1705	373.1705	0.0	0.0	18.5	1119.2	0.0	C27 H21 N2

Figure S41. HR-MS spectrum of **12**.

Elemental Composition Report Page 1

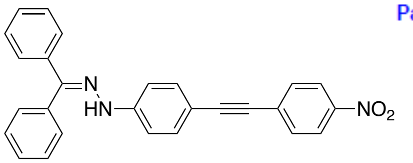

Single Mass Analysis
Tolerance = 10000.0 PPM / DBE: min = -5.5, max = 1000.0
Element prediction: Off
Number of isotope peaks used for i-FIT = 9

Monoisotopic Mass, Odd and Even Electron Ions
1 formula(e) evaluated with 1 results within limits (all results (up to 1000) for each mass)
Elements Used:
C: 27-27 H: 18-19 N: 3-3 O: 2-3
Kubra Erden
42213_20240109_01-N04 5 (0.206) Cm (1:10)

1: TOF MS ES-
1.88e+003

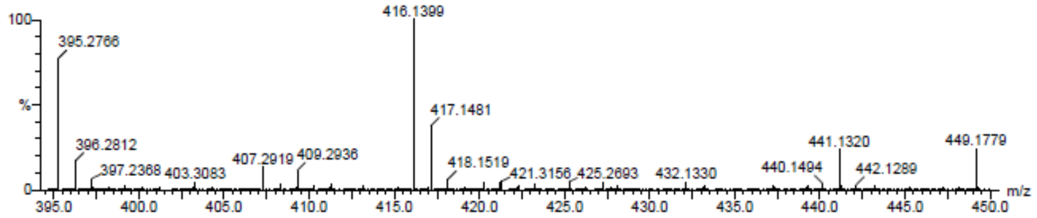

Minimum:				-5.5			
Maximum:		1000.0	10000.0	1000.0			
Mass	Calc. Mass	mDa	PPM	DBE	i-FIT	i-FIT (Norm)	Formula
416.1399	416.1399	0.0	0.0	20.5	421.0	0.0	C27 H18 N3 O2

Figure S42. HR-MS spectrum of **13**.

Elemental Composition Report Page 1

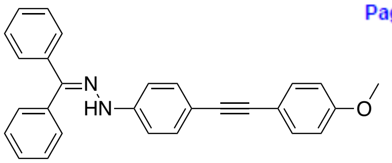

Single Mass Analysis
Tolerance = 10000.0 PPM / DBE: min = -5.5, max = 1000.0
Element prediction: Off
Number of isotope peaks used for i-FIT = 9

Monoisotopic Mass, Even Electron Ions
10 formula(e) evaluated with 1 results within limits (all results (up to 1000) for each mass)
Elements Used:
C: 28-28 H: 22-23 N: 1-3 O: 1-5
Kübra Erden
40401_20230623_01-04 8 (0.328) Cm (7:19)

1: TOF MS ES+
1.29e+004

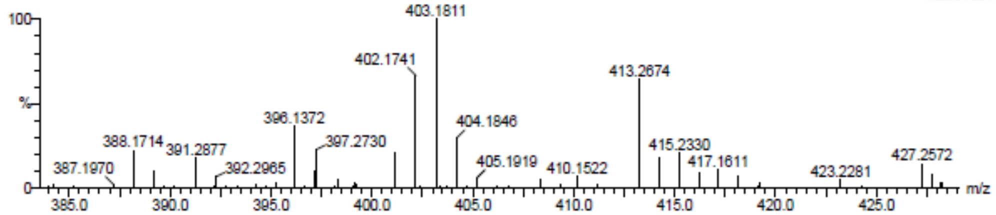

Minimum:				-5.5			
Maximum:		1000.0	10000.0	1000.0			
Mass	Calc. Mass	mDa	PPM	DBE	i-FIT	i-FIT (Norm)	Formula
403.1811	403.1810	0.1	0.2	18.5	968.5	0.0	C28 H23 N2 O

Figure S43. HR-MS spectrum of **14**.

Elemental Composition Report **Page 1**

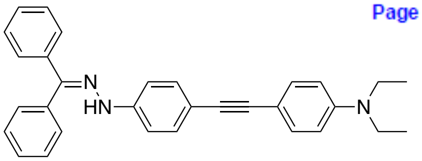

Single Mass Analysis
Tolerance = 10000.0 PPM / DBE: min = -5.5, max = 1000.0
Element prediction: Off
Number of isotope peaks used for i-FIT = 9

Monoisotopic Mass, Odd and Even Electron Ions
1 formula(e) evaluated with 1 results within limits (all results (up to 1000) for each mass)
Elements Used:
C: 31-31 H: 29-30 N: 3-3
Kübra Erden
41012_20230905_01-02 5 (0.206) Cm (1:6) 1: TOF MS ES+ 2.45e+004

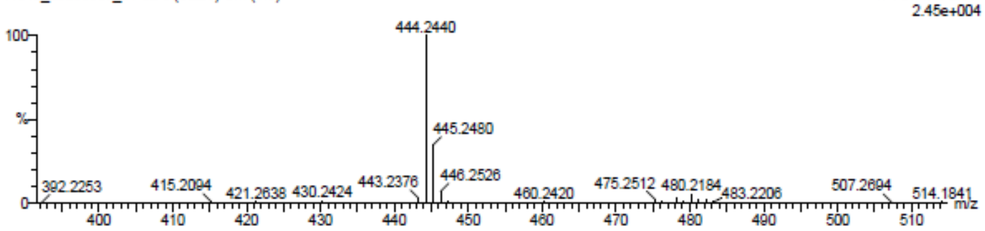

Minimum:				-5.5			
Maximum:		1000.0	10000.0	1000.0			
Mass	Calc. Mass	mDa	PPM	DBE	i-FIT	i-FIT (Norm)	Formula
444.2440	444.2440	0.0	0.0	18.5	799.4	0.0	C31 H30 N3

Figure S44. HR-MS spectrum of **15**.

Elemental Composition Report **Page 1**

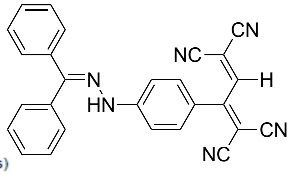

Single Mass Analysis
Tolerance = 1000.0 PPM / DBE: min = -5.5, max = 1000.0
Element prediction: Off
Number of isotope peaks used for i-FIT = 9

Monoisotopic Mass, Odd and Even Electron Ions
2 formula(e) evaluated with 1 results within limits (all results (up to 1000) for each mass)
Elements Used:
C: 27-27 H: 16-17 N: 1-6
Kubra Erden
42392_20240202_02-02 5 (0.206) Cm (5:25) 1: TOF MS ES+ 2.24e+003

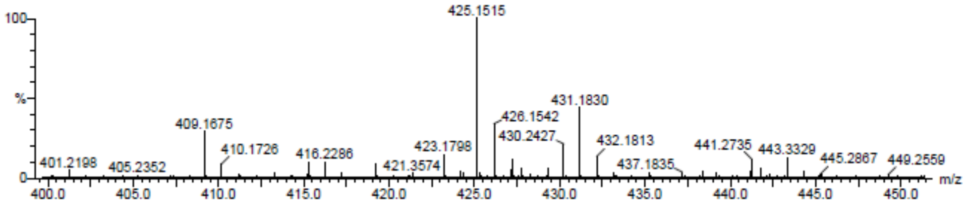

Minimum:				-5.5			
Maximum:		1000.0	1000.0	1000.0			
Mass	Calc. Mass	mDa	PPM	DBE	i-FIT	i-FIT (Norm)	Formula
425.1515	425.1515	0.0	0.0	22.5	875.8	0.0	C27 H17 N6

Figure S45. HR-MS spectrum of **17**.

Elemental Composition Report Page 1

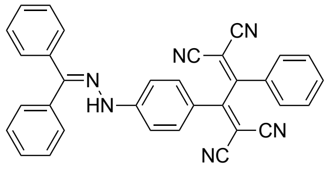

Single Mass Analysis
Tolerance = 10000.0 PPM / DBE: min = -5.5, max = 1000.0
Element prediction: Off
Number of isotope peaks used for i-FIT = 9

Monoisotopic Mass, Even Electron Ions
1 formula(e) evaluated with 1 results within limits (all results (up to 1000) for each mass)
Elements Used:
C: 33-33 H: 20-21 N: 6-6
Kübra Erden
40008_20230518_02-03 5 (0.206) Cm (2:6)

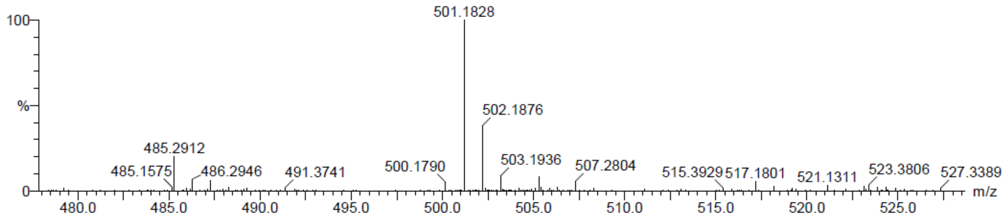

Minimum:				-5.5			
Maximum:		1000.0	10000.0	1000.0			
Mass	Calc. Mass	mDa	PPM	DBE	i-FIT	i-FIT (Norm)	Formula
501.1828	501.1828	0.0	0.0	26.5	782.1	0.0	C33 H21 N6

Figure S46. HR-MS spectrum of **18**.

Elemental Composition Report Page 1

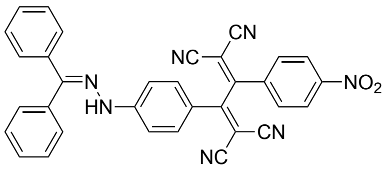

Single Mass Analysis
Tolerance = 10000.0 PPM / DBE: min = -5.5, max = 1000.0
Element prediction: Off
Number of isotope peaks used for i-FIT = 9

Monoisotopic Mass, Odd and Even Electron Ions
1 formula(e) evaluated with 1 results within limits (all results (up to 1000) for each mass)
Elements Used:
C: 33-33 H: 18-20 N: 7-7 O: 2-3
Kubra Erden
42213_20240109_02-01 23 (0.897) Cm (9:25)

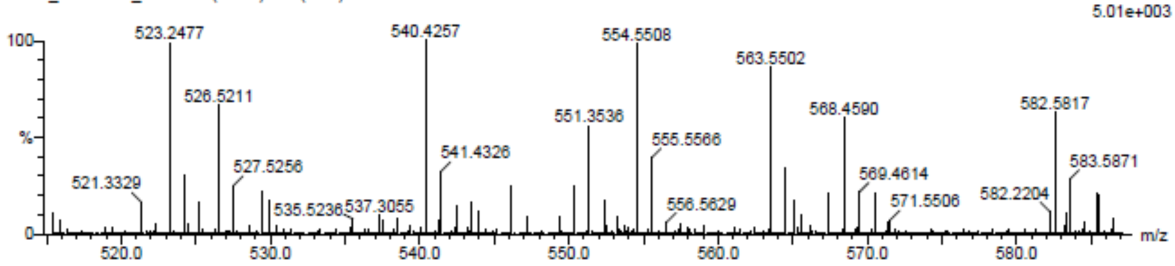

Minimum:				-5.5			
Maximum:		1000.0	10000.0	1000.0			
Mass	Calc. Mass	mDa	PPM	DBE	i-FIT	i-FIT (Norm)	Formula
546.1677	546.1678	-0.1	-0.2	27.5	716.1	0.0	C33 H20 N7 O2

Figure S47. HR-MS spectrum of **19**.

Elemental Composition Report

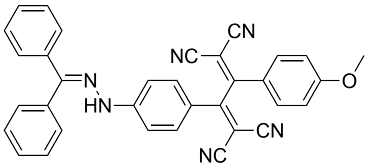

Page 1

Single Mass Analysis
Tolerance = 10000.0 PPM / DBE: min = -5.5, max = 1000.0
Element prediction: Off
Number of isotope peaks used for i-FIT = 9

Monoisotopic Mass, Even Electron Ions
7 formula(e) evaluated with 1 results within limits (all results (up to 1000) for each mass)
Elements Used:
C: 34-34 H: 22-23 N: 1-6 O: 1-5
Kübra Erden
40401_20230623_02-01 10 (0.396) Cm (4:25)
1: TOF MS ES+
2.12e+005

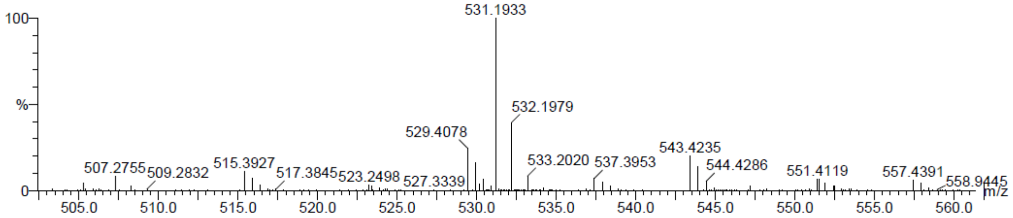

Minimum:				-5.5			
Maximum:		1000.0	10000.0	1000.0			
Mass	Calc. Mass	mDa	PPM	DBE	i-FIT	i-FIT (Norm)	Formula
531.1933	531.1933	0.0	0.0	26.5	1120.4	0.0	C34 H23 N6 O

Figure S48. HR-MS spectrum of **20**.

Elemental Composition Report

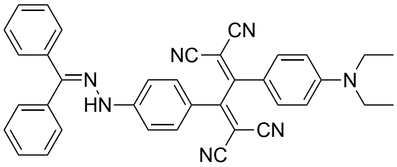

Page 1

Single Mass Analysis
Tolerance = 10000.0 PPM / DBE: min = -5.5, max = 1000.0
Element prediction: Off
Number of isotope peaks used for i-FIT = 9

Monoisotopic Mass, Odd and Even Electron Ions
1 formula(e) evaluated with 1 results within limits (all results (up to 1000) for each mass)
Elements Used:
C: 37-37 H: 29-30 N: 7-7
Kübra Erden
41859_20231218_01-02 11 (0.450) Cm (1:12)
1: TOF MS ES+
1.23e+004

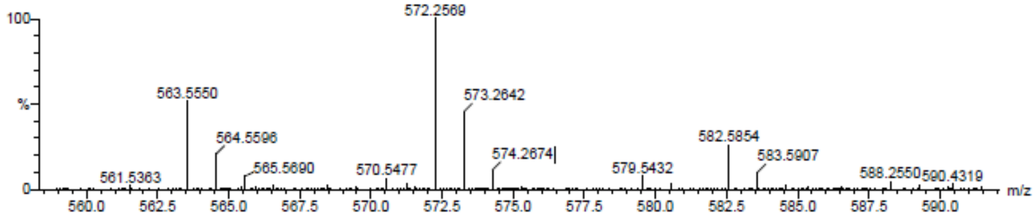

Minimum:				-5.5			
Maximum:		1000.0	10000.0	1000.0			
Mass	Calc. Mass	mDa	PPM	DBE	i-FIT	i-FIT (Norm)	Formula
572.2569	572.2563	0.6	1.0	26.5	675.8	0.0	C37 H30 N7

Figure S49. HR-MS spectrum of **21**.

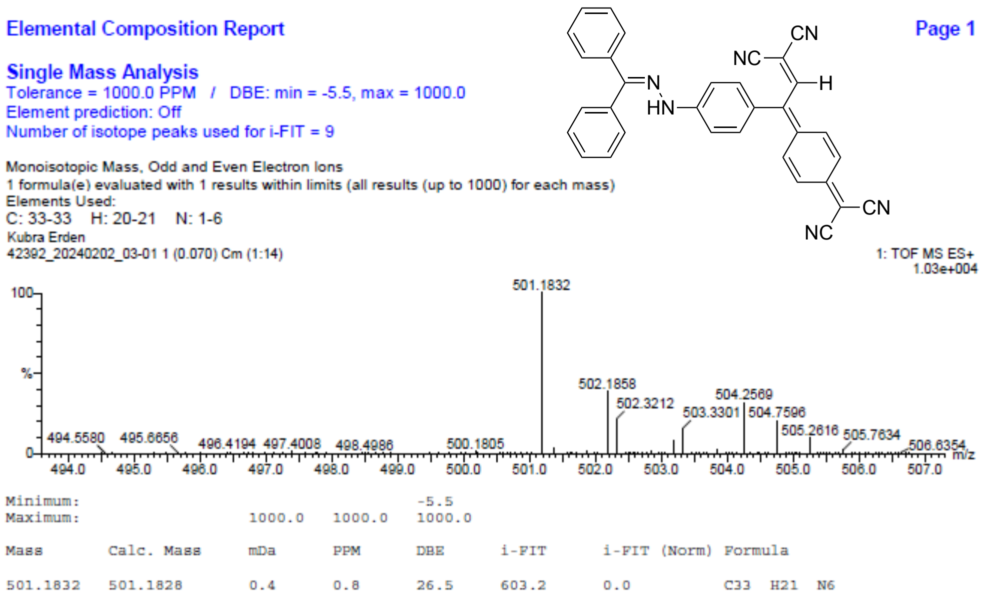

Figure S50. HR-MS spectrum of **23**.

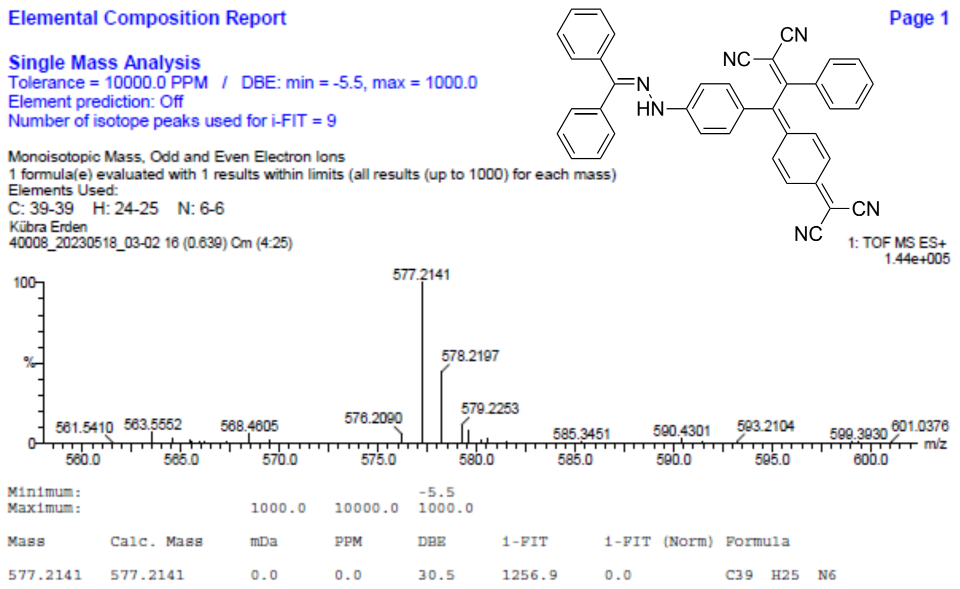

Figure S51. HR-MS spectrum of **24**.

Elemental Composition Report

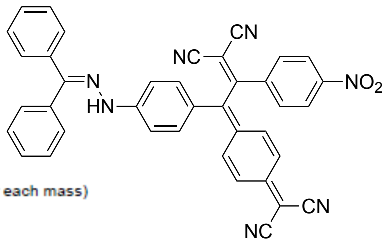

Page 1

Single Mass Analysis
Tolerance = 10000.0 PPM / DBE: min = -5.5, max = 1000.0
Element prediction: Off
Number of isotope peaks used for i-FIT = 9

Monoisotopic Mass, Odd and Even Electron Ions
1 formula(e) evaluated with 1 results within limits (all results (up to 1000) for each mass)
Elements Used:
C: 39-39 H: 22-23 N: 7-7 O: 2-3
Kubra Erden
42213_20240109_03-N03 2 (0.104) Cm (1:17)

1: TOF MS ES-
2.54e+003

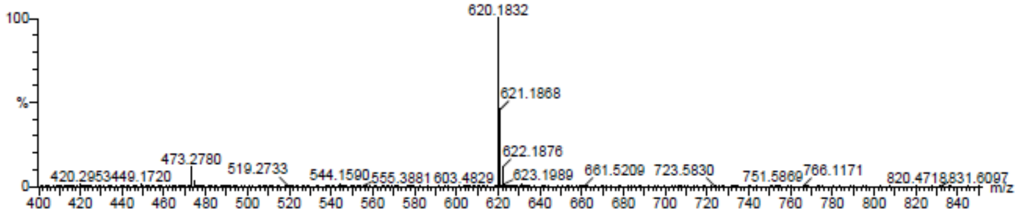

Minimum:				-5.5			
Maximum:		1000.0	10000.0	1000.0			
Mass	Calc. Mass	mDa	PPM	DBE	i-FIT	i-FIT (Norm)	Formula
620.1832	620.1835	-0.3	-0.5	32.5	229.8	0.0	C39 H22 N7 O2

Figure S52. HR-MS spectrum of **25**.

Elemental Composition Report

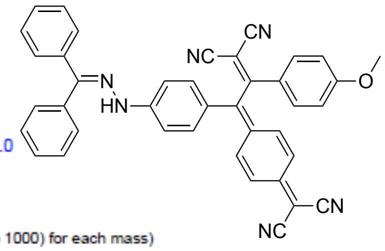

Page 1

Single Mass Analysis
Tolerance = 10000.0 PPM / DBE: min = -5.5, max = 1000.0
Element prediction: Off
Number of isotope peaks used for i-FIT = 9

Monoisotopic Mass, Odd and Even Electron Ions
6 formula(e) evaluated with 1 results within limits (all results (up to 1000) for each mass)
Elements Used:
C: 40-40 H: 26-27 N: 1-6 O: 1-5
Kübra Erden
40401_20230623_03-02 24 (0.931) Cm (11:25)

1: TOF MS ES+
2.18e+004

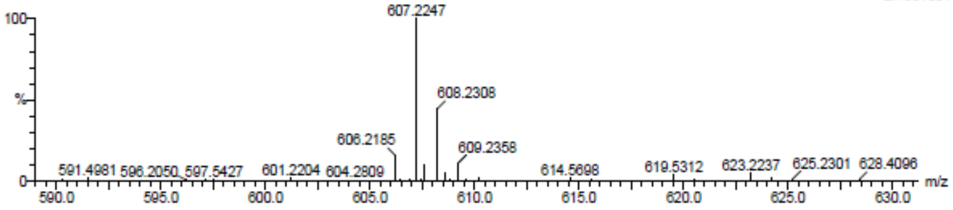

Minimum:				-5.5			
Maximum:		1000.0	10000.0	1000.0			
Mass	Calc. Mass	mDa	PPM	DBE	i-FIT	i-FIT (Norm)	Formula
607.2247	607.2246	0.1	0.2	30.5	962.3	0.0	C40 H27 N6 O

Figure S53. HR-MS spectrum of **26**.

Elemental Composition Report **Page 1**

Single Mass Analysis
Tolerance = 10000.0 PPM / DBE: min = -5.5, max = 1000.0
Element prediction: Off
Number of isotope peaks used for i-FIT = 9

Monoisotopic Mass, Odd and Even Electron Ions
1 formula(e) evaluated with 1 results within limits (all results (up to 1000) for each mass)
Elements Used:
C: 43-43 H: 33-34 N: 7-7
Kübra Erden
41859_20231218_02-01 21 (0.829) Cm (16:22)

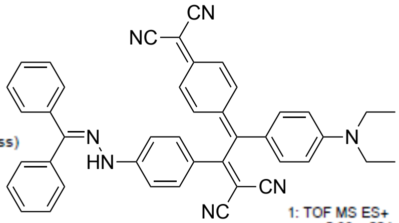

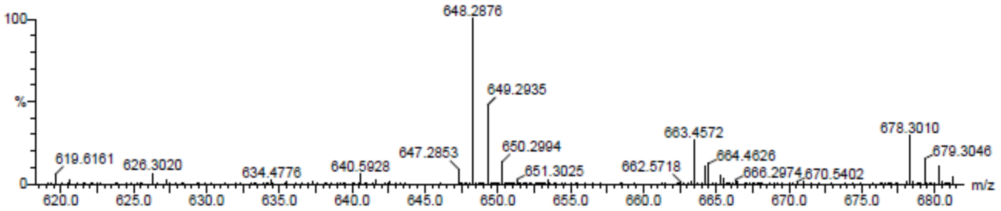

Minimum:				-5.5			
Maximum:		1000.0	10000.0	1000.0			
Mass	Calc. Mass	mDa	PPM	DBE	i-FIT	i-FIT (Norm)	Formula
648.2876	648.2876	0.0	0.0	30.5	813.2	0.0	C43 H34 N7

Figure S54. HR-MS spectrum of **27**.

3. Images

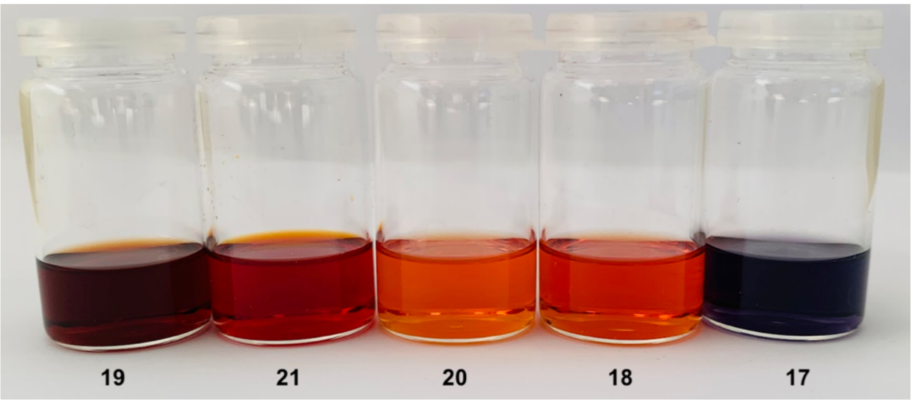

Figure S55. Photograph of chromophore **17**–**21** solutions in CH_2Cl_2.

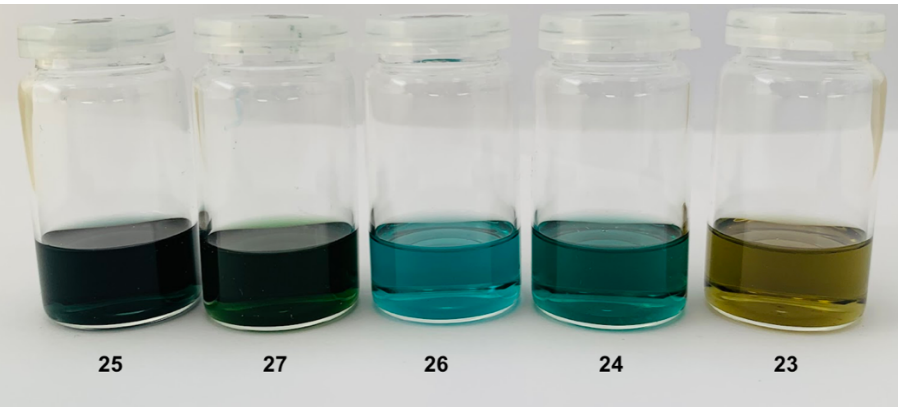

Figure S56. Photograph of chromophore **23**–**27** solutions in CH_2Cl_2.

4. Theoretical Calculations

General Computational Methods

Conformational searches were performed manually and automatically (using a Monte Carlo Multiple Minimum algorithm, employing the OPLS-2005 force field as implemented in MacroModel 9.7. In all cases, only those minima found within an energy threshold of 3 kcal mol^{-1} above the global minimum were considered for further optimization at the DFT level. The Gaussian 09 program package was employed exclusively for conducting all calculations. Geometry optimizations in CH_2Cl_2 were conducted using DFT with the CAM-B3LYP/6-31G++(d,p) basis set using the conductor-like solvation model of the polarizable continuum model (CPCM). On these minima, the vertical transition energies were calculated by time-dependent density functional theory (TD-DFT) at the CAM-B3LYP/6-31G++(d,p) level of theory, again with the PCM solvation model in CH_2Cl_2. The isosurfaces of the frontier orbitals (shown at 0.02 a.u.) were computed at the CAM-B3LYP/6-31G++(d,p) level of theory.

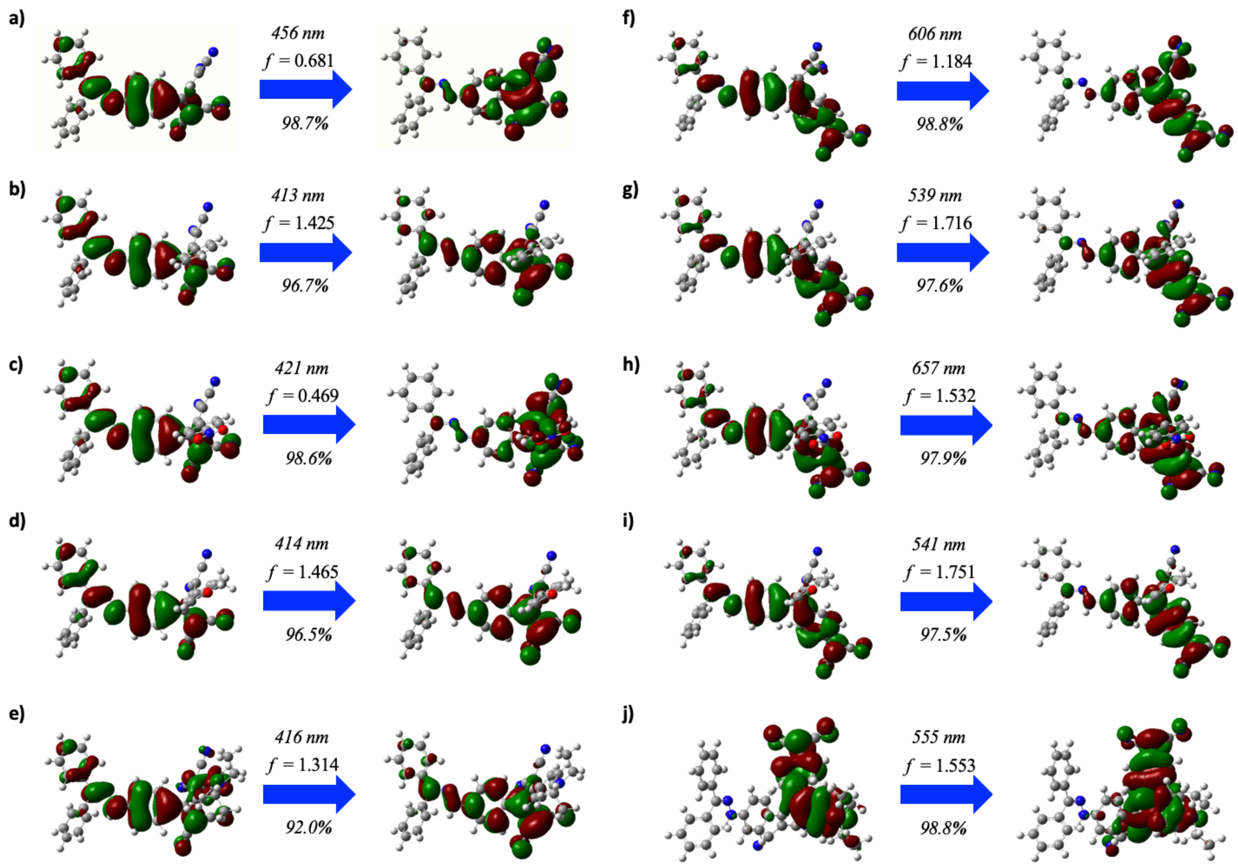

Figure S57. Natural transition orbitals for the calculated lowest energy UV/Vis absorption bands of compounds (a) **17**, (b) **18**, (c) **19**, (d) **20**, (e) **21**, (f) **23**, (g) **24**, (h) **25**, (i) **26**, (j) **27**.

Table S1. Depiction of calculated HOMOs and LUMOs over optimized ground-state geometries, transition energies (E), and oscillator strengths (f) for **17**.

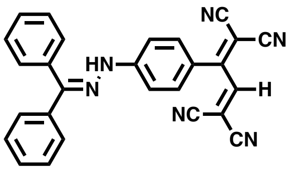

Exptl.: λ = 584 nm (in CH_2Cl_2)

**Excited state**	**ΔE (eV)**	**λ (nm)**	**f**	**assignments**
1	2.72	456	0.6811	H➔L
2	3.62	342	0.8671	H➔L+1
3	4.27	291	0.0243	H–5➔L
4	4.31	288	0.0988	H–5➔L, H–➔L

**Orbital**		**E (eV)**
HOMO–1	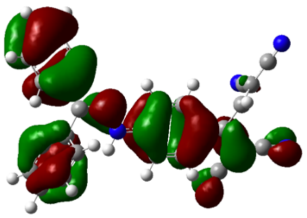	–8.58
HOMO	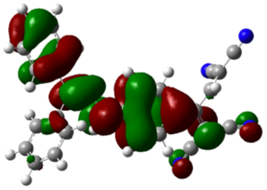	–7.11
LUMO	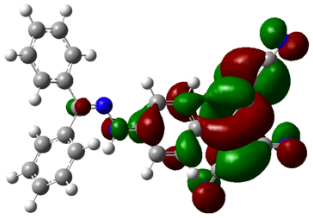	–2.48
LUMO+1	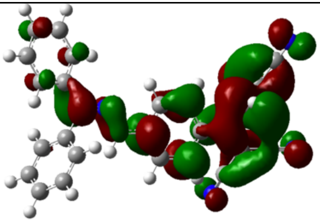	–1.35

17

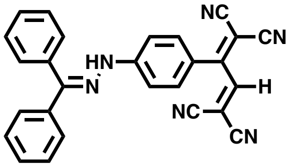

CAM-B3LYP/6-31G++(d,p) (CPCM solvation in DCM)

Sum of electronic and zero-point Energies= -1365.865209
Sum of electronic and thermal Energies= -1365.836724
Sum of electronic and thermal Enthalpies= -1365.835780
Sum of electronic and thermal Enthalpies= -1365.929977
Imaginary Freq = 0

Atom type, (x,y,z) coordinates

C	-0.54361700	-1.83000900	-0.29000600
C	0.20784500	-0.64252000	-0.19229200
C	-0.46533200	0.59165800	-0.16170200
C	-1.84291800	0.62089100	-0.21213700
C	-2.61094600	-0.55942400	-0.27119100
C	-1.91870500	-1.78678800	-0.32299000
C	-4.06115100	-0.47038800	-0.30660500
C	-4.61486800	0.75364100	-0.93127300
C	-4.93777400	-1.42551500	0.14016100
C	-6.35066200	-1.32715800	-0.07613000
C	-5.37542700	1.67103400	-0.30878000
N	-7.49562300	-1.28022300	-0.24067600
N	-4.28135000	-3.53818100	1.48335300
N	-6.22357700	3.76568000	-1.58200200
C	3.61625900	0.26381100	-0.00726400
C	4.41395700	1.50358000	0.15653700
C	4.31645700	-1.05309400	-0.11618200
C	5.78225400	1.51185500	-0.13427200
C	6.52935100	2.67917700	0.00081600
C	5.92178900	3.85309600	0.43381900
C	4.55895000	3.85286800	0.73277400
C	3.81237800	2.69055900	0.59801900
C	4.93145000	-1.61789400	1.00486000
C	5.57391800	-2.84818300	0.90793500
C	5.61312200	-3.52241500	-0.31057400
C	5.00468700	-2.96458600	-1.43182400
C	4.35563800	-1.73647200	-1.33527500
C	-4.54316200	-2.59298000	0.86843800
C	-5.84551900	2.83196000	-1.01592200
C	-5.73820400	1.58963000	1.08093500
N	-6.03219600	1.54893800	2.19765500
H	-0.03300300	-2.78567100	-0.35093900
H	0.10381400	1.50892200	-0.09779300
H	-2.33617900	1.58641700	-0.17796600
H	-2.45798400	-2.71900400	-0.43070800
H	6.26770700	0.60402900	-0.47476300
H	7.58870400	2.66772400	-0.23440300
H	6.50435500	4.76215800	0.54282400
H	4.07859600	4.76228500	1.07950800
H	2.75562400	2.68903100	0.83864500
H	4.90841200	-1.09048600	1.95352200
H	6.04662900	-3.27908700	1.78446600
H	6.11802900	-4.47994700	-0.38582000
H	5.03529500	-3.48352900	-2.38425300
H	3.88564100	-1.30083100	-2.21197300
N	2.33538600	0.38304600	-0.04289200
N	1.57271400	-0.72880400	-0.14588100
H	2.01225000	-1.64356300	-0.16796200
H	-4.34397400	0.93037500	-1.96854200

Table S2. Depiction of calculated HOMOs and LUMOs over optimized ground-state geometries, transition energies (*E*), and oscillator strengths (*f*) for **23**.

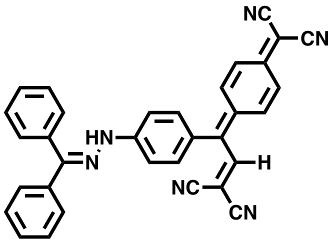

Exptl.: λ = 725 nm (in CH_2Cl_2)

**Excited state**	**Δ*E* (eV)**	***λ* (nm)**	***f***	**assignments**
1	2.05	606	1.1838	H➔L
2	3.02	411	0.3544	H–1➔L
3	3.48	356	0.8417	H➔L+1
4	3.80	327	0.0075	H–7➔L

**Orbital**		***E* (eV)**
HOMO–1	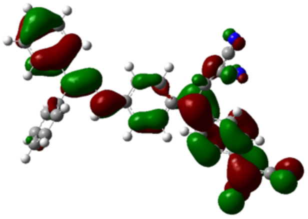	–7.76
HOMO	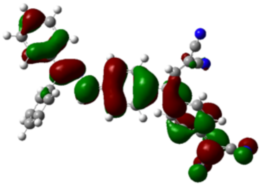	–6.85
LUMO	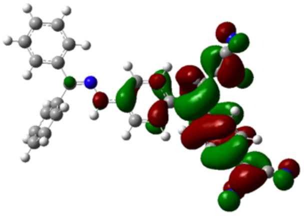	–3.05
LUMO+1	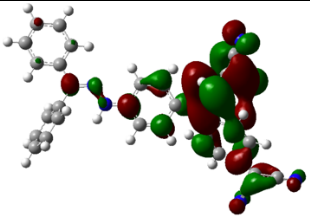	–1.34

23

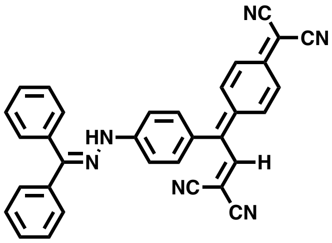

CAM-B3LYP/6-31G++(d,p) (CPCM solvation in DCM)

Sum of electronic and zero-point Energies= -1596.701949
Sum of electronic and thermal Energies= -1596.668863
Sum of electronic and thermal Enthalpies= -1596.667919
Sum of electronic and thermal Enthalpies= -1596.772900
Imaginary Freq = 0

Atom type, (x,y,z) coordinates

C	0.46679200	-1.05652800	-0.78137400
C	1.45672700	-0.13876700	-0.38418400
C	1.08159600	1.18120900	-0.07809500
C	-0.24522400	1.55334600	-0.15738900
C	-1.25480900	0.63868100	-0.51532900
C	-0.85598700	-0.67392200	-0.84055000
C	-2.64631000	1.06772600	-0.59880900
C	-2.83305100	2.44238800	-1.08478000
C	-3.58286400	3.39767100	-0.49253500
N	-3.71764200	5.77816000	-1.51279200
C	4.96136200	-0.13963400	0.08252700
C	6.00419900	0.80248700	0.55672200
C	5.35104500	-1.52331600	-0.32942000
C	7.35666300	0.55419600	0.29919400
C	8.33634200	1.44607400	0.72794200
C	7.97999300	2.59594300	1.42480100
C	6.63413100	2.84911600	1.69179400
C	5.65611000	1.96138900	1.26502800
C	5.75368600	-2.45710800	0.62965500
C	6.10336900	-3.74890800	0.24893400
C	6.05997500	-4.11815400	-1.09369500
C	5.66291500	-3.19244300	-2.05497300
C	5.30658600	-1.90115400	-1.67495200
C	-3.65762300	4.71678100	-1.05824900
C	-4.29586500	3.20842000	0.74059400
N	-4.86703300	3.09770000	1.73971200
H	0.74914400	-2.06765300	-1.05743100
H	1.83818000	1.89482400	0.21891400
H	-0.51036200	2.57500000	0.09622300
H	-1.59169300	-1.38985200	-1.18882000
H	7.64825300	-0.33717800	-0.24511400
H	9.38004900	1.23890400	0.51461600
H	8.74339200	3.28930400	1.76262900
H	6.34757100	3.73945900	2.24245000
H	4.61152100	2.15402600	1.48052400
H	5.79508300	-2.16937000	1.67569000
H	6.41253200	-4.46684400	1.00156800
H	6.33665500	-5.12477500	-1.38976600
H	5.63148000	-3.47297500	-3.10269400
H	5.00176300	-1.17931300	-2.42687700
N	3.74639400	0.28487500	0.05371700
N	2.76145700	-0.55797800	-0.32595400
H	2.98231200	-1.52059900	-0.55960800
C	-3.73331800	0.26126000	-0.29574500
C	-5.06689200	0.55739300	-0.76913700
C	-3.59204900	-0.93537000	0.50300500
C	-6.11768800	-0.25325200	-0.50784900
H	-5.22373000	1.42330600	-1.40089500
C	-4.64674700	-1.73071400	0.79587600
H	-2.62736700	-1.15973400	0.93976300
H	-7.09425400	-0.01564600	-0.91367900
H	-4.50900400	-2.59017300	1.44193900
C	-5.96387100	-1.44196200	0.29046700
C	-7.04423700	-2.26884000	0.57204000
C	-8.35339100	-1.98266200	0.09327400
C	-6.90150500	-3.44933800	1.35375600
N	-9.41918900	-1.74701500	-0.29992600
N	-6.78107800	-4.41134200	1.99106700
H	-2.26224300	2.73643800	-1.96145600

Table S3. Depiction of calculated HOMOs and LUMOs over optimized ground-state geometries, transition energies (*E*), and oscillator strengths (*f*) for **18**.

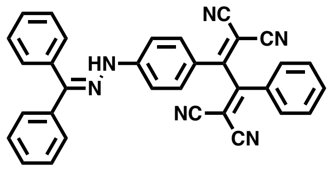

Exptl.: λ = 488 nm (in CH_2Cl_2)

**Excited state**	**ΔE (eV)**	**λ (nm)**	***f***	**assignments**
1	3.00	413	1.4254	H➔L, H➔L+1
2	3.25	381	0.0253	H➔L, H➔L+1
3	4.09	303	0.4716	H–3➔L
4	4.29	289	0.1254	H➔L+2

**Orbital**		***E* (eV)**
HOMO–1	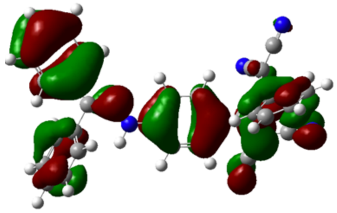	-8.54
HOMO	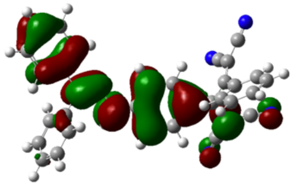	–7.12
LUMO	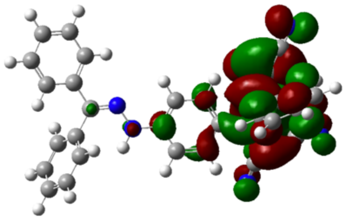	–2.04
LUMO+1	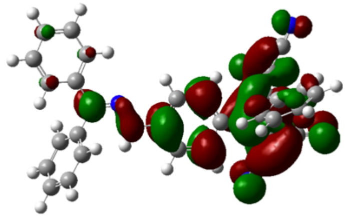	–2.01

18

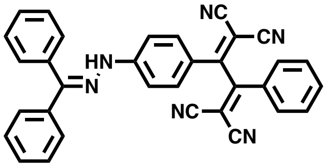

CAM-B3LYP/6-31G++(d,p) (CPCM solvation in DCM)

Sum of electronic and zero-point Energies= -1596.718771
Sum of electronic and thermal Energies= -1596.685587
Sum of electronic and thermal Enthalpies= -1596.684642
Sum of electronic and thermal Enthalpies= -1596.789686
Imaginary Freq = 0

Atom type, (x,y,z) coordinates

C	-0.27832700	-1.98704800	-0.02581200
C	-0.99116800	-0.77726900	-0.14805600
C	-0.27745600	0.41352600	-0.37533300
C	1.09493800	0.37985800	-0.48796700
C	1.82348300	-0.82691500	-0.40785000
C	1.08993400	-2.00811500	-0.15633900
C	3.26831900	-0.82306300	-0.53809800
C	3.98967200	0.45892000	-0.23146800
C	4.06409500	-1.88217500	-0.89469700
C	5.49162000	-1.76532700	-0.89186900
C	4.40289900	0.68652000	1.16442400
C	4.23646800	1.32050900	-1.25298500
N	6.64712600	-1.68916400	-0.89224000
N	3.24970100	-4.18605300	-1.74353900
C	3.56923000	0.29709400	2.22402100
C	3.96281300	0.51097100	3.53770700
C	5.19991000	1.08852900	3.81359100
C	6.04630100	1.45014400	2.76881700
C	5.65306000	1.25309000	1.45200400
N	5.23291200	3.69773900	-0.99059900
C	-4.35601900	0.27629300	0.00051000
C	-5.12326100	1.53363100	-0.17274600
C	-5.08242400	-0.99387600	0.30976800
C	-6.45251600	1.61919000	0.25451100
C	-7.16993900	2.80361800	0.10784100
C	-6.57153600	3.91728300	-0.47191300
C	-5.24788400	3.83960000	-0.90652200
C	-4.53094800	2.66027100	-0.76083600
C	-5.83585000	-1.63255700	-0.67920300
C	-6.50041600	-2.82188700	-0.39662600
C	-6.42274100	-3.38043800	0.87725900
C	-5.67593700	-2.74814400	1.86771100
C	-5.00516900	-1.56106600	1.58533300
C	3.58368000	-3.15054500	-1.34739400
C	4.80435800	2.62716300	-1.07838400
C	3.88330200	0.99834700	-2.60748800
N	3.60176200	0.75720500	-3.70309400
H	-0.81297100	-2.90744900	0.18529200
H	-0.81286700	1.34940800	-0.45724500
H	1.60999400	1.31766900	-0.65754000
H	1.59767600	-2.95332800	-0.02050200
H	2.60389700	-0.15181300	2.02404800
H	3.30254700	0.22137700	4.34782900
H	5.50843300	1.24629000	4.84163300
H	7.02078500	1.87786100	2.97730800
H	6.33763000	1.50425800	0.65080700
H	-6.93025200	0.75901000	0.71005700
H	-8.19883500	2.85277300	0.44931600
H	-7.13131700	4.83948100	-0.58961600
H	-4.77579400	4.70140400	-1.36736400
H	-3.50550000	2.59819400	-1.10598700
H	-5.90268400	-1.19516100	-1.67059600
H	-7.08135700	-3.31104000	-1.17162600
H	-6.94452400	-4.30601300	1.09752300
H	-5.61495100	-3.17695100	2.86257000
H	-4.42654900	-1.06733700	2.36038100
N	-3.07736800	0.33758400	-0.13184100
N	-2.35099900	-0.79886200	-0.02376300
H	-2.82023600	-1.68457800	0.13777400

Table S4. Depiction of calculated HOMOs and LUMOs over optimized ground-state geometries, transition energies (*E*), and oscillator strengths (*f*) for **24**.

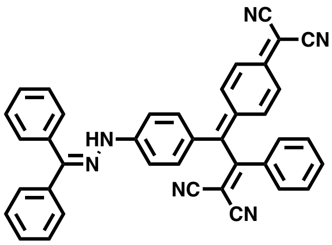

Exptl.: λ = 641 nm (in CH_2Cl_2)

**Excited state**	**Δ*E* (eV)**	***λ* (nm)**	***f***	**assignments**
1	2.30	539	1.7155	H➔L
2	3.03	409	0.1277	H➔L+1
3	3.45	359	0.3014	H–1➔L
4	3.97	312	0.2283	H–1➔L+1, H➔L+2

**Orbital**		***E* (eV)**
HOMO–1	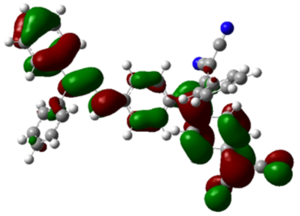	-7.77
HOMO	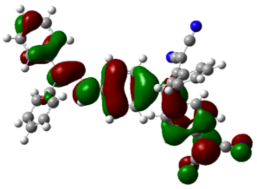	–6.83
LUMO	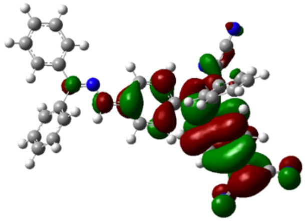	–2.67
LUMO+1	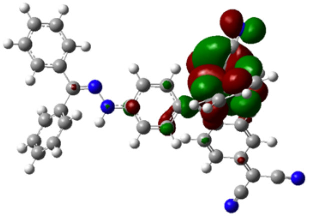	–1.92

24

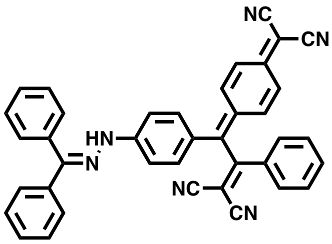

CAM-B3LYP/6-31G++(d,p) (CPCM solvation in DCM)

Sum of electronic and zero-point Energies= -1827.553355
Sum of electronic and thermal Energies= -1827.515484
Sum of electronic and thermal Enthalpies= -1827.514539
Sum of electronic and thermal Enthalpies= -1827.630722
Imaginary Freq = 0

Atom type, (x,y,z) coordinates

C	0.83507000	1.26831800	0.73690300
C	1.80192400	0.43610100	0.14135300
C	1.38024300	-0.72464300	-0.53197000
C	0.03657300	-1.02366600	-0.61308800
C	-0.95053800	-0.17722900	-0.06657300
C	-0.50448800	0.96783100	0.62978000
C	-2.36592700	-0.50623300	-0.15725000
C	-2.70383700	-1.96667000	-0.10613600
C	-2.81079300	-2.61759500	1.21432700
C	-2.89841700	-2.62056200	-1.28341900
C	-1.94126000	-2.27146100	2.25981200
C	-2.05964300	-2.88260000	3.50072500
C	-3.06080900	-3.82392500	3.72770400
C	-3.94685400	-4.15273000	2.70562100
C	-3.82338300	-3.55761100	1.45708600
N	-3.19070300	-5.18217000	-1.54614600
C	5.32110500	0.34190500	-0.15461400
C	6.34375900	-0.49942300	-0.82241100
C	5.75173700	1.53489800	0.63756700
C	7.68951000	-0.41227100	-0.45041700
C	8.64877800	-1.21373400	-1.06398300
C	8.27858300	-2.10943800	-2.06168400
C	6.93973800	-2.19882500	-2.44400900
C	5.98198800	-1.40116600	-1.83329700
C	6.24730800	2.66963900	-0.01111100
C	6.63741100	3.78463800	0.72408500
C	6.54157400	3.77425900	2.11387400
C	6.05140500	2.64646600	2.76687200
C	5.65467100	1.53201600	2.03244400
C	-3.07306800	-4.04119500	-1.39666800
C	-2.87301100	-1.92490400	-2.54033000
N	-2.85141500	-1.39334100	-3.56760300
H	1.14846900	2.14321100	1.29775400
H	2.11677400	-1.37743500	-0.98040300
H	-0.25267200	-1.92700700	-1.13750800
H	-1.22142100	1.60014500	1.13993600
H	-1.15714200	-1.54254100	2.09895200
H	-1.36911800	-2.61854500	4.29429500
H	-3.15587000	-4.29274000	4.70141400
H	-4.74235000	-4.86892400	2.88005900
H	-4.54065100	-3.80350600	0.68287700
H	7.99138300	0.28023200	0.32743200
H	9.68722000	-1.13570000	-0.75862800
H	9.02633600	-2.73146500	-2.54294400
H	6.64330400	-2.88871400	-3.22769700
H	4.94358100	-1.46471500	-2.13710300
H	6.32948900	2.67701300	-1.09363400
H	7.01894700	4.66127300	0.21088700
H	6.84974300	4.64299200	2.68642700
H	5.97847800	2.63120300	3.84937700
H	5.27711300	0.65173100	2.54418400
N	4.08778700	0.00181600	-0.29665000
N	3.12437300	0.77160900	0.25678100
H	3.37875200	1.61820500	0.75565600
C	-3.39473500	0.41011800	-0.26424900
C	-4.77427900	0.02086200	-0.07414700
C	-3.16891800	1.79373800	-0.61716600
C	-5.79017600	0.91092800	-0.14962400
H	-5.00585400	-1.00747300	0.17659500
C	-4.18436200	2.68292700	-0.71696300
H	-2.16712800	2.11419700	-0.87189200
H	-6.80757800	0.58327100	0.03032900
H	-3.97752100	3.70254700	-1.02140900
C	-5.54692600	2.29520200	-0.46211700
C	-6.58898400	3.21317700	-0.53952100
C	-7.94119300	2.83285400	-0.31577800
C	-6.35912800	4.58302100	-0.84558700
N	-9.04303600	2.51854100	-0.13151500
N	-6.16645900	5.69992400	-1.09503700

Table S5. Depiction of calculated HOMOs and LUMOs over optimized ground-state geometries, transition energies (*E*), and oscillator strengths (*f*) for **19**.

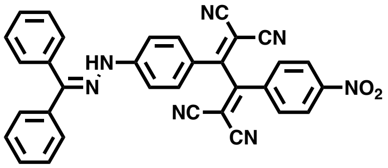

Exptl.: λ = 473 nm (in CH_2Cl_2)

**Excited state**	**Δ*E* (eV)**	**λ (nm)**	***f***	**assignments**
1	2.95	421	0.4691	H➔L, H➔L+1
2	3.00	414	0.9749	H➔L, H➔L+1
3	3.92	317	0.0010	H–10➔L
4	4.19	296	0.5682	H–6➔L

**Orbital**		***E* (eV)**
HOMO–1	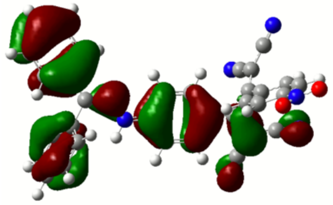	–8.59
HOMO	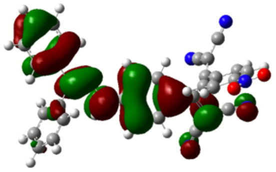	–7.17
LUMO	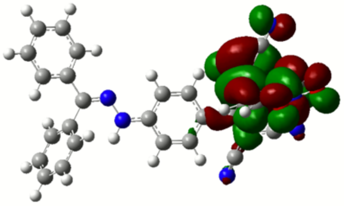	–2.55
LUMO+1	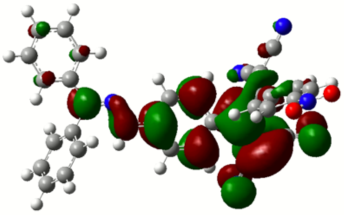	–2.09

19

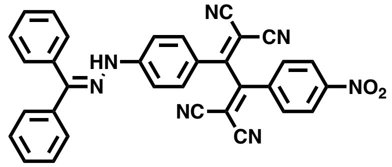

CAM-B3LYP/6-31G++(d,p) (CPCM solvation in DCM)

**Sum of electronic and zero-point Energies=**	**-1801.161277**
**Sum of electronic and thermal Energies=**	**-1801.125466**
**Sum of electronic and thermal Enthalpies=**	**-1801.124522**
**Sum of electronic and thermal Enthalpies=**	**-1801.236770**
**Imaginary Freq = 0**	

Atom type, (x,y,z) coordinates

C	-0.84632000	1.67057900	-1.24262400
C	-1.53227600	0.84379600	-0.32937900
C	-0.81763100	0.26608600	0.73650000
C	0.52673400	0.52678800	0.88180700
C	1.22184000	1.38552300	0.00201900
C	0.49189900	1.93489800	-1.07636000
C	2.63365100	1.65247800	0.19583000
C	3.43688300	0.62755300	0.94367200
C	3.33477000	2.74151300	-0.25643800
C	4.75306100	2.83044700	-0.07783300
C	4.03898200	-0.47083400	0.15446100
C	3.59541800	0.75840800	2.28270200
N	5.90028200	2.91094700	0.05761700
N	2.32343500	4.84806500	-1.36610900
C	3.29204400	-1.12588800	-0.83377800
C	3.85908300	-2.15047400	-1.57480300
C	5.18176800	-2.49191700	-1.33169100
C	5.95577000	-1.84576300	-0.37981100
C	5.37587600	-0.83031400	0.36505000
N	4.76962400	-0.98186100	3.80303200
C	-4.82051300	-0.35311300	0.12011800
C	-5.56897000	-1.16766300	1.10790000
C	-5.53878000	0.21141400	-1.06373700
C	-6.82003300	-1.70295500	0.78372600
C	-7.51712700	-2.48300700	1.70259000
C	-6.97722800	-2.73374600	2.95975400
C	-5.73310700	-2.19817900	3.29447400
C	-5.03603000	-1.42133300	2.37959600
C	-6.43810800	1.26912600	-0.90180200
C	-7.09740400	1.80437700	-2.00386300
C	-6.86908600	1.28395100	-3.27583700
C	-5.97681900	0.22840000	-3.44372600
C	-5.31082600	-0.30435000	-2.34308100
C	2.74871600	3.88721500	-0.87997300
C	4.26094900	-0.21558000	3.10283600
C	3.05277400	1.88173800	2.99672400
N	2.61910200	2.77614600	3.58698700
H	-1.37715300	2.09279800	-2.08957600
H	-1.33080300	-0.38114900	1.43428500
H	1.04284400	0.06147900	1.71293200
H	0.98528200	2.54712000	-1.81904800
H	2.26054500	-0.85250800	-1.01613400
H	3.28928500	-2.67486700	-2.33016200
H	6.98997000	-2.12647100	-0.23148300
H	5.98096000	-0.29854200	1.08871600
H	-7.25067500	-1.51587100	-0.19362800
H	-8.48411300	-2.89453000	1.43207900
H	-7.52191500	-3.33830700	3.67771900
H	-5.30882900	-2.38182800	4.27639300
H	-4.07361600	-0.99825900	2.64298100
H	-6.62229600	1.67120500	0.08967600
H	-7.79177600	2.62709800	-1.86839200
H	-7.38681600	1.69969100	-4.13405900
H	-5.79814900	-0.18334000	-4.43158700
H	-4.61877400	-1.13065000	-2.47569100
N	-3.56643400	-0.16185300	0.33697100
N	-2.86431100	0.61664900	-0.51953900
H	-3.33574500	1.05643200	-1.30394600
N	5.78832200	-3.57122600	-2.12121200
O	6.95439300	-3.86318800	-1.89388500
O	5.09584100	-4.12339100	-2.96554500

Table S6. Depiction of calculated HOMOs and LUMOs over optimized ground-state geometries, transition energies (*E*), and oscillator strengths (*f*) for **25**.

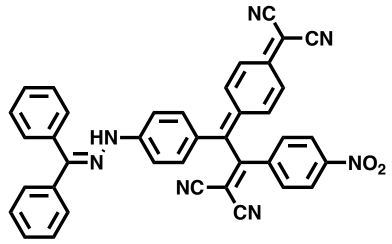

Exptl.: λ = 657 nm (in CH_2Cl_2)

Excited state	ΔE (eV)	λ (nm)	f	assignments
1	2.29	541	1.5324	H➔L
2	2.77	448	0.2767	H➔L+1
3	3.40	365	0.3241	H–1➔L
4	3.76	330	0.0980	H–1➔L+1

Orbital		*E* (eV)
HOMO–1	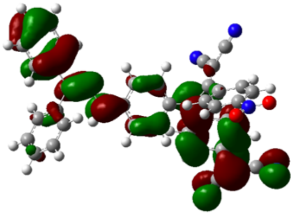	–7.83
HOMO	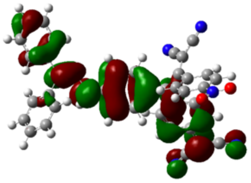	–6.88
LUMO	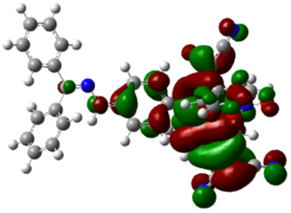	–2.78
LUMO+1	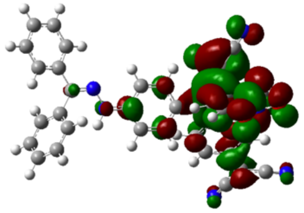	–2.41

25

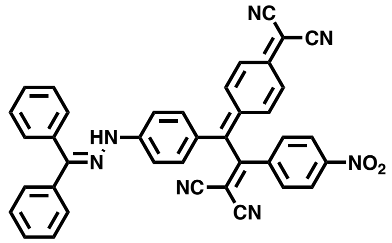

CAM-B3LYP/6-31G++(d,p) (CPCM solvation in DCM)

Sum of electronic and zero-point Energies= -2031.996291
Sum of electronic and thermal Energies= -2031.955774
Sum of electronic and thermal Enthalpies= -2031.954829
Sum of electronic and thermal Enthalpies= -2032.078191
Imaginary Freq = 0

Atom type, (x,y,z) coordinates

C	1.10006300	-1.21136200	-0.99850200
C	2.04896100	-0.63526500	-0.13278900
C	1.59971900	0.10207100	0.97769200
C	0.24722800	0.24300700	1.20901400
C	-0.71730300	-0.36283400	0.37808800
C	-0.24695200	-1.07982500	-0.74327600
C	-2.14439800	-0.20036200	0.62864200
C	-2.56308900	1.14158400	1.14821400
C	-2.73301100	2.25145100	0.17910800
C	-2.77014900	1.29516400	2.48033000
C	-1.80021200	2.45927700	-0.84601000
C	-1.97234300	3.49222400	-1.75378500
C	-3.09661800	4.29720200	-1.63994200
C	-4.05137400	4.10465800	-0.65317500
C	-3.86237500	3.07705000	0.25832500
N	-3.24750500	3.55705900	3.65385600
C	5.56819200	-0.41323800	0.08640700
C	6.57555300	0.11735900	1.03685500
C	6.02293100	-1.08416200	-1.16994100
C	7.89319500	0.34318300	0.62457200
C	8.83531300	0.85857400	1.51086300
C	8.47660700	1.14973900	2.82303600
C	7.16671700	0.92120200	3.24524400
C	6.22580300	0.40826400	2.36295300
C	6.65269700	-2.33094600	-1.11627500
C	7.06567500	-2.96009000	-2.28654400
C	6.85978800	-2.34670000	-3.52036100
C	6.23651100	-1.10312200	-3.58121300
C	5.81638800	-0.47524800	-2.41172900
C	-3.04468500	2.55911000	3.10637000
C	-2.66396400	0.18653200	3.38953400
N	-2.57759700	-0.68402500	4.14568100
H	1.43198300	-1.74984100	-1.88050000
H	2.32163600	0.55760400	1.64180900
H	-0.06421000	0.81986100	2.07252100
H	-0.95496100	-1.49509100	-1.45090000
H	-0.92473800	1.82917700	-0.92787600
H	-1.24955700	3.67100700	-2.53862300
H	-4.92392500	4.74225000	-0.60281500
H	-4.61718200	2.90875600	1.01693800
H	8.18536800	0.12258500	-0.39617900
H	9.85142900	1.03239400	1.17173600
H	9.21168500	1.54738800	3.51526800
H	6.88057300	1.13677600	4.26976800
H	5.21070700	0.22195600	2.69402000
H	6.82102500	-2.80710200	-0.15525200
H	7.55132000	-3.92902100	-2.23421500
H	7.18608600	-2.83647600	-4.43200600
H	6.07779500	-0.61845400	-4.53896300
H	5.33496900	0.49690700	-2.46120600
N	4.32741800	-0.27163200	0.39865800
N	3.37995900	-0.79173600	-0.41344500
H	3.65370200	-1.31975000	-1.23597700
C	-3.10875200	-1.16022800	0.40042500
C	-4.51967100	-0.83888100	0.41846300
C	-2.77654100	-2.54963500	0.16973400
C	-5.47156900	-1.76548400	0.16829800
H	-4.82957500	0.18336900	0.59970500
C	-3.72657800	-3.48469700	-0.05799400
H	-1.74251400	-2.86073300	0.24207500
H	-6.51627500	-1.47669400	0.16266300
H	-3.43936400	-4.52170500	-0.18893500
C	-5.12444200	-3.13884600	-0.09624700
C	-6.09965100	-4.09378200	-0.35278100
C	-7.48452100	-3.76678200	-0.36932900
C	-5.76763700	-5.45267700	-0.61387800
N	-8.61254300	-3.49574500	-0.38303600
N	-5.49225500	-6.55947200	-0.82662200
N	-3.28638600	5.38757400	-2.60377000
O	-4.28835600	6.08154500	-2.49583200
O	-2.43247500	5.54717800	-3.46575800

Table S7. Depiction of calculated HOMOs and LUMOs over optimized ground-state geometries, transition energies (*E*), and oscillator strengths (*f*) for **20**.

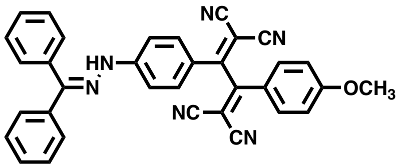

Exptl.: λ = 487 nm (in CH_2Cl_2)

**Excited state**	**Δ*E* (eV)**	***λ* (nm)**	***f***	**assignments**
1	2.99	414	1.4653	H➔L
2	3.31	375	0.0586	H➔L+1
3	3.65	340	0.5773	H–1➔L+1
4	3.93	316	0.0459	H–1➔L

**Orbital**		***E* (eV)**
HOMO–1	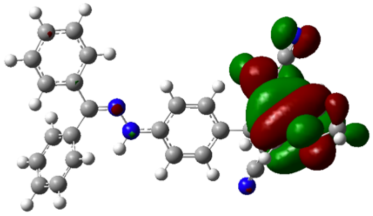	–8.02
HOMO	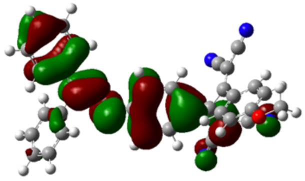	–7.11
LUMO	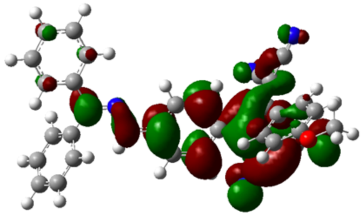	–2.01
LUMO+1	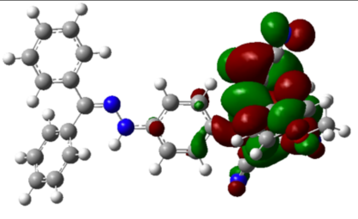	–1.95

20

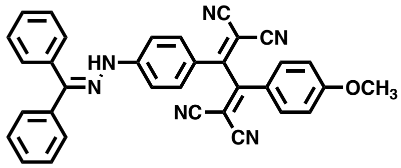

CAM-B3LYP/6-31G++(d,p) (CPCM solvation in DCM)

Sum of electronic and zero-point Energies= -1711.175061
Sum of electronic and thermal Energies= -1711.139306
Sum of electronic and thermal Enthalpies= -1711.138362
Sum of electronic and thermal Enthalpies= -1711.249699
Imaginary Freq = 0

Atom type, (x,y,z) coordinates

C	0.75888100	2.01352000	0.62773300
C	1.42231000	0.89040600	0.09352600
C	0.67578500	-0.06335200	-0.62188700
C	-0.67682800	0.12032600	-0.80410300
C	-1.35386400	1.25796400	-0.31119200
C	-0.58932800	2.19042500	0.42674400
C	-2.77809900	1.41350900	-0.53434900
C	-3.56297000	0.18224400	-0.90021100
C	-3.51255600	2.56971300	-0.44066000
C	-4.93448600	2.55215300	-0.61284800
C	-4.13625100	-0.59704100	0.19403200
C	-3.69059700	-0.10426600	-2.22902200
N	-6.08480900	2.55974600	-0.74674700
N	-2.59195900	4.95942000	-0.07809000
C	-3.50887800	-0.62420100	1.45728000
C	-4.04089200	-1.35266200	2.49801400
C	-5.23887000	-2.06054400	2.32371100
C	-5.89783800	-2.01576700	1.09086200
C	-5.34516700	-1.29307400	0.04563100
N	-4.71135300	-2.27102300	-3.21218400
C	4.70933900	-0.39098500	0.00845800
C	5.42364600	-1.53682900	-0.60560600
C	5.47404400	0.60190900	0.82411600
C	6.70115200	-1.89749700	-0.16440800
C	7.36618700	-2.98299100	-0.72884600
C	6.76711900	-3.71967100	-1.74524100
C	5.49563900	-3.36356400	-2.19605500
C	4.83075800	-2.28255600	-1.63421800
C	6.35254200	1.49380500	0.20230000
C	7.05665600	2.42655000	0.95717200
C	6.89414500	2.47279400	2.34006800
C	6.02302500	1.58542700	2.96625100
C	5.31253200	0.65531800	2.21186500
C	-2.97376200	3.87630300	-0.22704400
C	-4.27555700	-1.30635500	-2.74449400
C	-3.17096100	0.77738800	-3.23424400
N	-2.75596100	1.47362700	-4.06029700
H	1.31503400	2.74045200	1.21059200
H	1.17094600	-0.93643100	-1.02402100
H	-1.21726700	-0.63740900	-1.35806100
H	-1.05814300	3.05159900	0.88194500
H	-2.58134600	-0.08829300	1.61852200
H	-3.54773500	-1.38809400	3.46263600
H	-6.84111700	-2.52334900	0.93975100
H	-5.89769700	-1.24044100	-0.88383300
H	7.17842600	-1.33267500	0.62864500
H	8.35483500	-3.25117200	-0.37062300
H	7.28675900	-4.56313500	-2.18819200
H	5.02443900	-3.92755900	-2.99470400
H	3.84670100	-2.00122600	-1.99064100
H	6.48568100	1.45429900	-0.87440800
H	7.73472500	3.11598300	0.46494000
H	7.44666600	3.19822100	2.92821100
H	5.89563800	1.61429600	4.04341000
H	4.63734000	-0.03916300	2.70289800
N	3.44304400	-0.30093800	-0.20229900
N	2.76539600	0.75650300	0.30127200
H	3.26251800	1.47588500	0.81721000
O	-5.68331700	-2.73955900	3.39695000
C	-6.89306400	-3.48826700	3.29072200
H	-6.80570200	-4.26203400	2.52291500
H	-7.03833400	-3.95302400	4.26392300
H	-7.73911000	-2.83238900	3.06737200

Table S8. Depiction of calculated HOMOs and LUMOs over optimized ground-state geometries, transition energies (*E*), and oscillator strengths (*f*) for **26**.

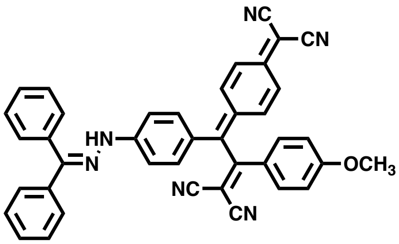

Exptl.: λ = 635 nm (in CH_2Cl_2)

**Excited state**	**ΔE (eV)**	**λ (nm)**	***f***	**assignments**
1	2.29	541	1.7509	H→L
2	3.07	403	0.1213	H→L+1
3	3.44	361	0.2784	H–1→L
4	3.52	352	0.1131	H–2→L

**Orbital**		***E* (eV)**
HOMO–1	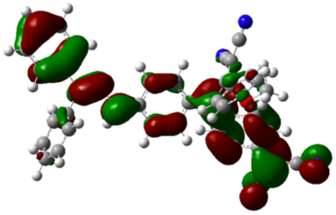	–7.75
HOMO	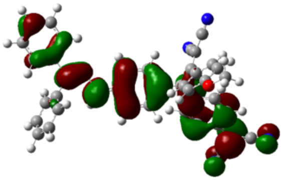	–6.80
LUMO	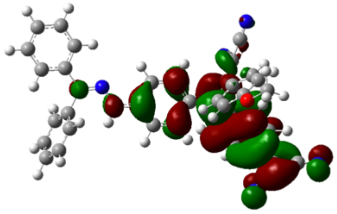	–2.65
LUMO+1	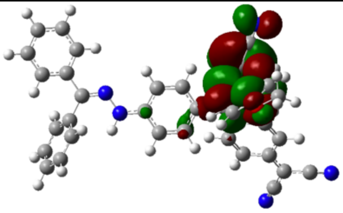	–1.86

26

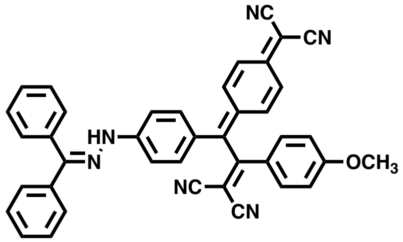

CAM-B3LYP/6-31G++(d,p) (CPCM solvation in DCM)

Sum of electronic and zero-point Energies= -1942.009492
Sum of electronic and thermal Energies= -1941.969058
Sum of electronic and thermal Enthalpies= -1941.968114
Sum of electronic and thermal Enthalpies= -1942.089892
Imaginary Freq = 0

Atom type, (x,y,z) coordinates

C	-1.08589000	-1.27966300	0.88671000
C	-2.03192800	-0.55846900	0.13370500
C	-1.57813600	0.36763400	-0.82280100
C	-0.22544500	0.54468300	-1.02219200
C	0.73838200	-0.20551500	-0.31580600
C	0.26144700	-1.10687000	0.66227900
C	2.16274700	-0.00625300	-0.53419300
C	2.58628000	1.38594100	-0.90554000
C	2.80861700	2.35354600	0.17019400
C	2.73450500	1.65571900	-2.23705900
C	2.10033000	2.25737200	1.38602700
C	2.31634500	3.15616100	2.40767900
C	3.27360700	4.17069700	2.26578000
C	4.01338900	4.26301800	1.08314500
C	3.77440500	3.36438400	0.05471100
N	3.11390600	3.99821800	-3.27457300
C	-5.55042700	-0.30825500	-0.05753600
C	-6.55758500	0.42215200	-0.86481400
C	-6.00407500	-1.26075500	1.00253400
C	-7.89402500	0.47580800	-0.45494500
C	-8.83821600	1.17532100	-1.20229100
C	-8.46228000	1.82723900	-2.37192000
C	-7.13262400	1.77462600	-2.79183400
C	-6.19006300	1.07817600	-2.04841900
C	-6.55848600	-2.49464100	0.65029100
C	-6.96789800	-3.38733400	1.63594500
C	-6.83186600	-3.05367600	2.98167100
C	-6.28255300	-1.82539500	3.33969200
C	-5.86706400	-0.93305400	2.35481300
C	2.95979800	2.96241700	-2.78163700
C	2.59440500	0.62567400	-3.22632700
N	2.48014900	-0.18052800	-4.04898800
H	-1.42060400	-1.96302600	1.66079500
H	-2.29725500	0.93717700	-1.39570100
H	0.08854400	1.26727100	-1.76638300
H	0.96427000	-1.63786900	1.29314400
H	1.35412900	1.48502800	1.52264900
H	1.75639200	3.09129400	3.33363900
H	4.78034100	5.01556300	0.95737800
H	4.38979000	3.43605700	-0.83328500
H	-8.20055200	-0.02536400	0.45640100
H	-9.86945200	1.20927800	-0.86598700
H	-9.19828500	2.36973400	-2.95642800
H	-6.83181700	2.27399700	-3.70733400
H	-5.15902000	1.03185800	-2.37917300
H	-6.67121600	-2.75378900	-0.39794600
H	-7.39544200	-4.34344700	1.35222100
H	-7.15463300	-3.74933900	3.74935700
H	-6.17775200	-1.55866400	4.38622800
H	-5.44294500	0.02634500	2.63591700
N	-4.30870400	-0.08790000	-0.31513300
N	-3.36373400	-0.76628100	0.37293700
H	-3.64029300	-1.44835100	1.07190100
C	3.13594200	-0.98162400	-0.41572800
C	4.54021000	-0.64176000	-0.37319900
C	2.82403800	-2.39214200	-0.38075100
C	5.50418500	-1.58104800	-0.23214000
H	4.83514800	0.40028100	-0.41351700
C	3.78663800	-3.33697800	-0.26381300
H	1.79798400	-2.70745600	-0.51849800
H	6.54383000	-1.28020900	-0.17236100
H	3.51391100	-4.38601100	-0.28020600
C	5.17618700	-2.98029300	-0.15383700
C	6.16445300	-3.94855800	-0.00300900
C	7.54212900	-3.60630000	0.08108200
C	5.84960300	-5.33290500	0.07799700
N	8.66526600	-3.32197900	0.15068100
N	5.58700600	-6.46152100	0.14430600
O	3.42034600	4.99542000	3.31975700
C	4.37938400	6.04878500	3.24588700
H	4.31011300	6.57757500	4.19450800
H	5.38955400	5.64842300	3.12210300
H	4.14448400	6.73303500	2.42582900

Table S9. Depiction of calculated HOMOs and LUMOs over optimized ground-state geometries, transition energies (*E*), and oscillator strengths (*f*) for **21**.

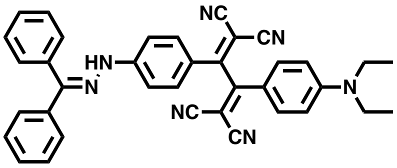

Exptl.: λ = 478 nm (in CH_2Cl_2)

Excited state	Δ*E* (eV)	*λ* (nm)	*f*	assignments
1	2.98	416	1.3143	H➔L
2	3.07	404	0.8947	H➔L+1
3	3.24	383	0.2301	H–1➔L
4	3.52	352	0.0889	H–1➔L+1

Orbital		*E* (eV)
HOMO–1	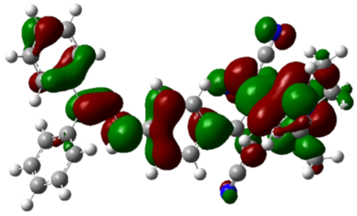	–7.16
HOMO	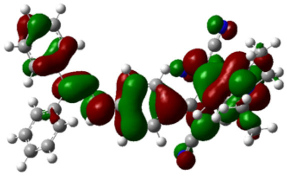	–7.01
LUMO	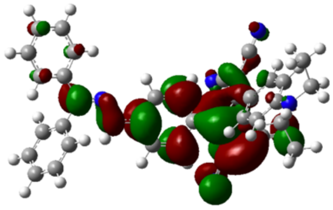	–1.93
LUMO+1	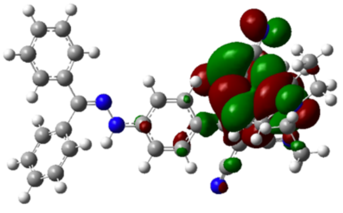	–1.80

21

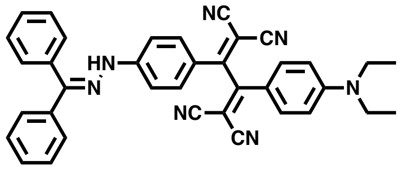

CAM-B3LYP/6-31G++(d,p) (CPCM solvation in DCM)

Sum of electronic and zero-point Energies= -1809.090587
Sum of electronic and thermal Energies= -1809.050675
Sum of electronic and thermal Enthalpies= -1809.049731
Sum of electronic and thermal Enthalpies= -1809.170242
Imaginary Freq = 0

Atom type, (x,y,z) coordinates

C	1.35228700	-1.66876900	-1.46954600
C	1.95904500	-0.89850700	-0.45758500
C	1.19860600	-0.53506200	0.66806100
C	-0.11141600	-0.94807900	0.77279800
C	-0.72742200	-1.75271800	-0.21078400
C	0.04778100	-2.08506700	-1.34479300
C	-2.10880200	-2.16437600	-0.04919500
C	-2.94405400	-1.41902100	0.96322600
C	-2.75917600	-3.17213300	-0.71720500
C	-4.15642600	-3.40980000	-0.50801700
C	-3.63408000	-0.24366700	0.50164000
C	-2.95089500	-1.96730400	2.22974700
N	-5.28550800	-3.62200600	-0.36136700
N	-1.70914700	-4.88863400	-2.34238000
C	-3.31867900	0.31801900	-0.75926100
C	-3.95526800	1.42969200	-1.25107400
C	-4.98987800	2.07205300	-0.51651200
C	-5.32744200	1.49639100	0.74050100
C	-4.67377600	0.39063100	1.22275200
N	-4.03102300	-0.96006100	4.35153100
C	5.11883600	0.55237400	0.15498800
C	5.79000500	1.29918700	1.24668800
C	5.88046500	0.22538000	-1.08942600
C	6.96923100	2.01060300	1.00106800
C	7.59072000	2.72708100	2.02049400
C	7.04656100	2.73834000	3.30059800
C	5.87436000	2.02632900	3.55666000
C	5.25292300	1.31201800	2.54163300
C	6.90876900	-0.72091500	-1.05640500
C	7.61072700	-1.03578700	-2.21561100
C	7.29649800	-0.40371600	-3.41671500
C	6.27562800	0.54217200	-3.45638500
C	5.56753600	0.85382600	-2.29869300
C	-2.15081400	-4.09897400	-1.61936800
C	-3.56714000	-1.39095900	3.38084300
C	-2.25414000	-3.18569800	2.50674200
N	-1.69428700	-4.16861400	2.75886100
H	1.91682300	-1.92874400	-2.35927300
H	1.64883400	0.06794600	1.44465600
H	-0.66693800	-0.64764200	1.65288800
H	-0.37928400	-2.65033100	-2.16117800
H	-2.54241400	-0.12587300	-1.37089400
H	-3.66435900	1.79641500	-2.22542200
H	-6.11040600	1.92912200	1.34724000
H	-5.00085100	-0.00064300	2.17558400
H	7.40247700	2.01222900	0.00702000
H	8.50203300	3.27753900	1.81024800
H	7.53279400	3.29329900	4.09638700
H	5.44800900	2.02224000	4.55473700
H	4.34757400	0.75124900	2.74296100
H	7.15985300	-1.20910500	-0.11981400
H	8.40567100	-1.77349100	-2.17987200
H	7.84723400	-0.64731100	-4.31939000
H	6.02915600	1.04047000	-4.38821700
H	4.77471800	1.59526100	-2.33101500
N	3.89223600	0.20896500	0.33961200
N	3.26228200	-0.51746100	-0.61189600
H	3.76888600	-0.81043100	-1.44135000
N	-5.62712800	3.16795700	-0.99148800
C	-6.76053300	3.77372300	-0.29072600
H	-7.38786600	4.24748200	-1.04957500
H	-7.37248000	2.98834700	0.15720800
C	-5.22663800	3.81946800	-2.23921800
H	-4.13968000	3.78227200	-2.33505900
H	-5.48451000	4.87706300	-2.14385000
C	-6.34180300	4.80376500	0.75440000
H	-7.22690800	5.24219500	1.22372400
H	-5.72823800	4.35080600	1.53735300
H	-5.76506400	5.61170300	0.29545500
C	-5.90447800	3.22876300	-3.47242400
H	-5.58884400	3.77030500	-4.36856100
H	-5.64849700	2.17405700	-3.60168400
H	-6.99241600	3.30698100	-3.39379500

Table S10. Depiction of calculated HOMOs and LUMOs over optimized ground-state geometries, transition energies (*E*), and oscillator strengths (*f*) for **27**.

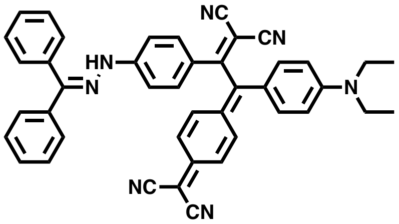

Exptl.: λ = 683 nm (in CH_2Cl_2)

**Excited state**	**Δ*E* (eV)**	***λ* (nm)**	***f***	**assignments**
1	2.24	555	1.5533	H➔L
2	2.60	477	0.1707	H–1➔L
3	3.19	389	0.6494	H–1➔L+1, H➔L+1
4	3.37	368	0.4459	H➔L+1, H–2➔L

**Orbital**		***E* (eV)**
HOMO–1	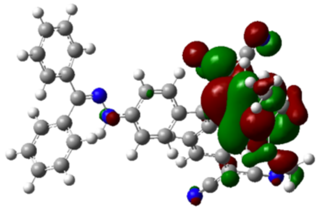	–7.08
HOMO	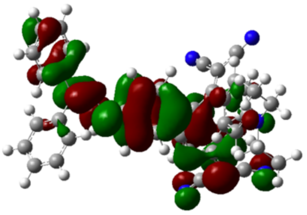	–6.77
LUMO	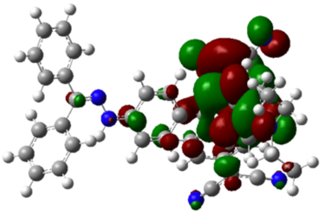	–2.70
LUMO+1	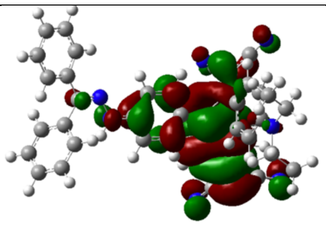	–1.61

27

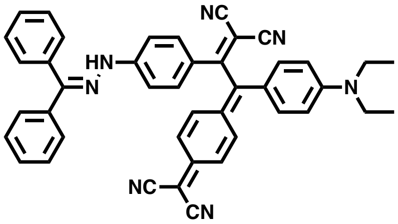

CAM-B3LYP/6-31G++(d,p) (CPCM solvation in DCM)

Sum of electronic and zero-point Energies= -2039.926288
Sum of electronic and thermal Energies= -2039.881623
Sum of electronic and thermal Enthalpies= -2039.880679
Sum of electronic and thermal Enthalpies= -2040.012199
Imaginary Freq = 0

Atom type, (x,y,z) coordinates

C	1.13417900	-1.47946900	-1.02141300
C	2.08491200	-0.96101700	-0.12341500
C	1.64049300	-0.32432400	1.04914000
C	0.28929300	-0.22752800	1.30787100
C	-0.67763800	-0.78162200	0.44432200
C	-0.21184600	-1.39331300	-0.73829200
C	-2.09598400	-0.66293100	0.74595400
C	-2.50495300	0.59394500	1.46116500
C	-2.45327400	1.83007900	0.71591600
C	-2.87465400	0.45365100	2.78111700
C	-2.51360600	1.80243400	-0.69502400
C	-2.49540400	2.94789600	-1.45390200
C	-2.38564100	4.22913100	-0.84890600
C	-2.28159200	4.25662400	0.56912000
C	-2.32326100	3.10239000	1.31407200
N	-3.87675400	2.30133400	4.28946000
C	5.60548600	-0.71080000	0.07502900
C	6.61550700	-0.22041000	1.04420100
C	6.05550800	-1.31327200	-1.21743500
C	7.93581100	0.00800700	0.64204700
C	8.88111600	0.48495200	1.54656900
C	8.52294600	0.73571400	2.86704100
C	7.21006000	0.50492900	3.27908000
C	6.26633000	0.02989600	2.37893400
C	6.66494100	-2.57115000	-1.23469600
C	7.07225200	-3.13797900	-2.43835600
C	6.87986900	-2.45128500	-3.63514500
C	6.27588100	-1.19680000	-3.62540000
C	5.86213000	-0.63082400	-2.42238900
C	-3.41237000	1.49835400	3.59508300
C	-2.77563300	-0.80554900	3.45541400
N	-2.69686600	-1.80188400	4.04190500
H	1.46279900	-1.93525700	-1.95010800
H	2.36472800	0.08818800	1.73848900
H	-0.02521300	0.26814800	2.22000800
H	-0.92120700	-1.75911200	-1.47139000
H	-2.60894700	0.85282000	-1.21019900
H	-2.58399900	2.85285400	-2.52711000
H	-2.13731000	5.19365000	1.08886500
H	-2.20332900	3.19399200	2.38559300
H	8.22830700	-0.18060900	-0.38496500
H	9.89933700	0.66063500	1.21470200
H	9.26015000	1.10382500	3.57321900
H	6.92358300	0.68951900	4.30957300
H	5.24885200	-0.15709300	2.70213800
H	6.82201000	-3.10496700	-0.30245200
H	7.54247500	-4.11590800	-2.44096200
H	7.20131500	-2.89276600	-4.57283400
H	6.12700500	-0.65555600	-4.55399900
H	5.39532300	0.34967400	-2.41662900
N	4.36562900	-0.59684700	0.40169000
N	3.41623700	-1.07396600	-0.43208200
H	3.68660800	-1.54128700	-1.29157100
C	-3.06278200	-1.59289800	0.41012200
C	-4.47021200	-1.26560300	0.46243700
C	-2.73963200	-2.94300600	0.00867600
C	-5.42760600	-2.14505700	0.08608800
H	-4.76939600	-0.27060100	0.77091200
C	-3.69544100	-3.83449700	-0.34369300
H	-1.70915200	-3.27095300	0.05311500
H	-6.46994500	-1.84840700	0.10809400
H	-3.41525600	-4.84970600	-0.60060100
C	-5.08856600	-3.47292200	-0.35361000
C	-6.06907900	-4.37681600	-0.75118200
C	-7.44981700	-4.03641800	-0.74391400
C	-5.74344100	-5.68898800	-1.19209000
N	-8.57559100	-3.75423600	-0.73828100
N	-5.47282400	-6.75858900	-1.55236700
N	-2.36857400	5.36773500	-1.58634300
C	-2.36086000	5.34602500	-3.04860800
H	-1.75096900	4.50971700	-3.39672500
H	-1.84562100	6.25146900	-3.37911800
C	-2.36654000	6.69304500	-0.96801800
H	-2.87728800	7.36929900	-1.65832700
H	-2.97607000	6.67146700	-0.06230300
C	-3.75860300	5.29301800	-3.65963400
H	-4.29104500	4.38684300	-3.35955200
H	-4.35334900	6.15546000	-3.34574600
H	-3.69186400	5.30555300	-4.75119100
C	-0.96498600	7.21839000	-0.66962600
H	-1.02409200	8.22009000	-0.23465700
H	-0.43771300	6.56982200	0.03472700
H	-0.36928100	7.28032800	-1.58478000

5. Electrochemistry

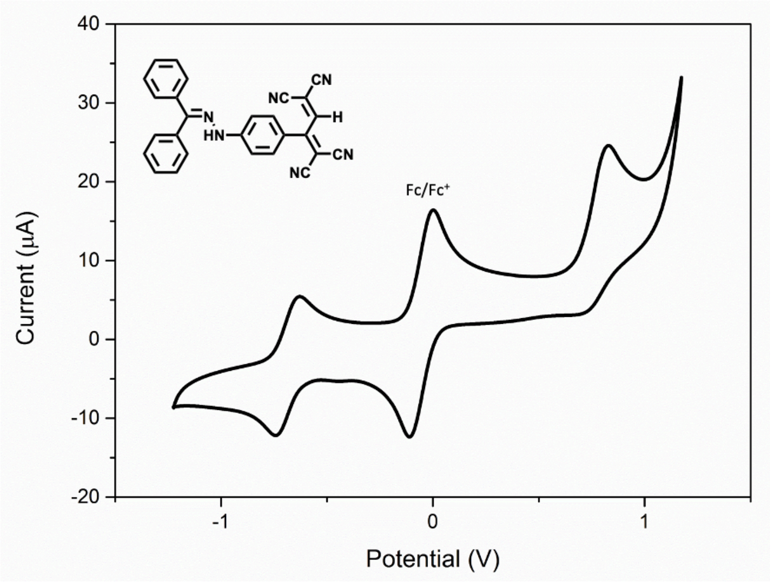

Figure S58. Cyclic voltammogram of **17** at a scan rate of 100 mVs^{-1} in DCM + 0.1 M Bu_4NPF_6. All potentials are indicated versus ferrocene/ferrocenium redox couple used as an internal reference (given as IUPAC convention).

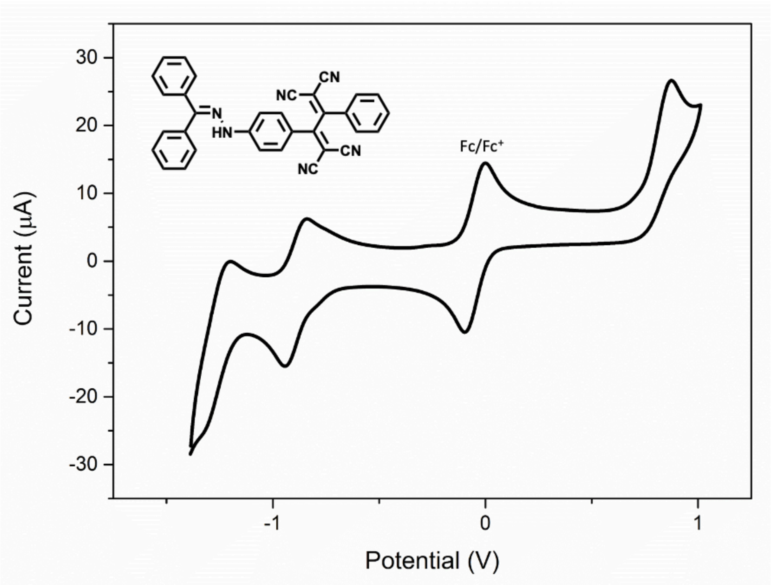

Figure S59. Cyclic voltammogram of **18** at a scan rate of 100 mVs^{-1} in DCM + 0.1 M Bu_4NPF_6. All potentials are indicated versus ferrocene/ferrocenium redox couple used as an internal reference (given as IUPAC convention).

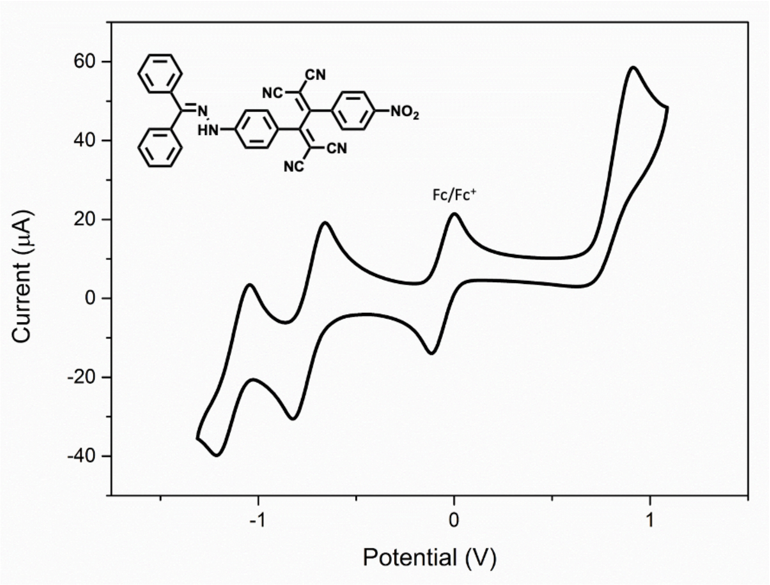

Figure S60. Cyclic voltammogram of **19** at a scan rate of 100 mVs^{-1} in DCM + 0.1 M Bu_4NPF_6. All potentials are indicated versus ferrocene/ferrocenium redox couple used as an internal reference (given as IUPAC convention).

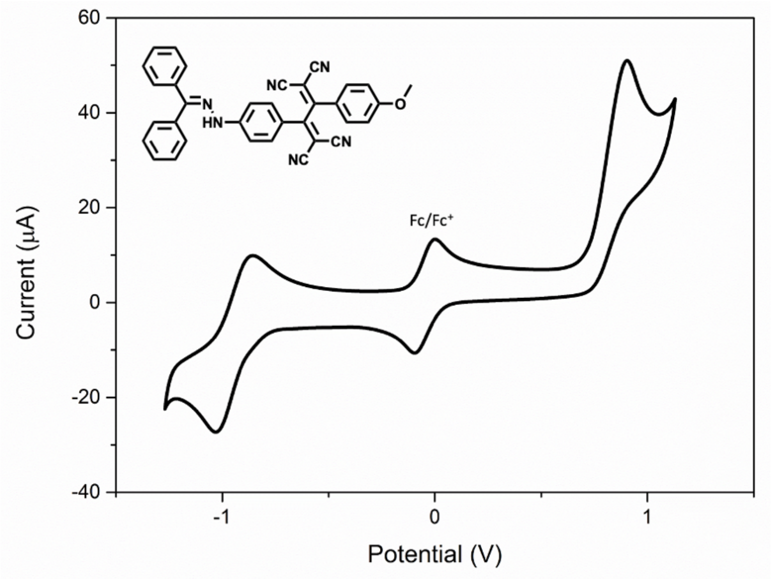

Figure S61. Cyclic voltammogram of **20** at a scan rate of 100 mVs^{-1} in DCM + 0.1 M Bu_4NPF_6. All potentials are indicated versus ferrocene/ferrocenium redox couple used as an internal reference (given as IUPAC convention).

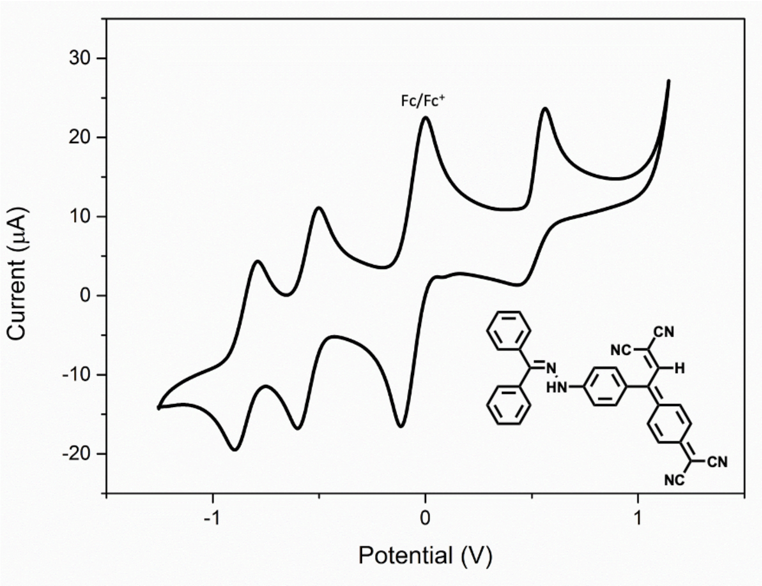

Figure S62. Cyclic voltammogram of **23** at a scan rate of 100 mVs^{-1} in DCM + 0.1 M Bu_4NPF_6. All potentials are indicated versus ferrocene/ferrocenium redox couple used as an internal reference (given as IUPAC convention).

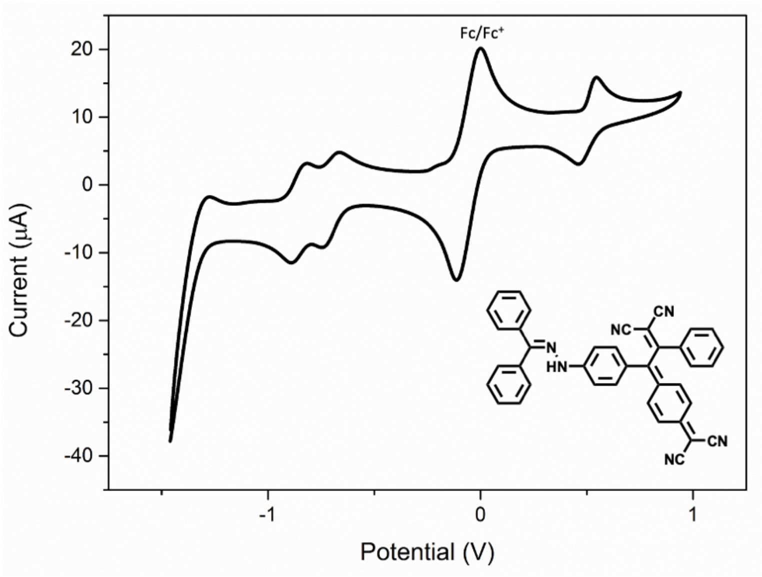

Figure S63. Cyclic voltammogram of **24** at a scan rate of 100 mVs^{-1} in DCM + 0.1 M Bu_4NPF_6. All potentials are indicated versus ferrocene/ferrocenium redox couple used as an internal reference (given as IUPAC convention).

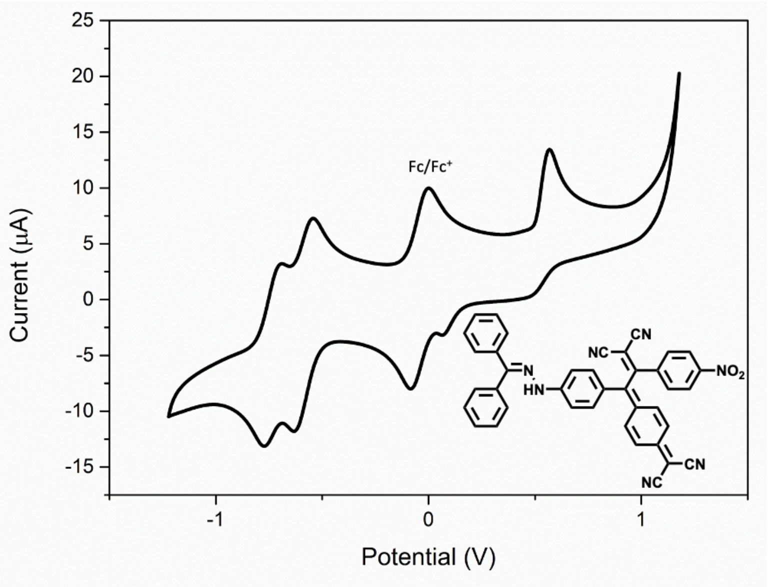

Figure S64. Cyclic voltammogram of **25** at a scan rate of 100 mVs^{-1} in DCM + 0.1 M Bu_4NPF_6. All potentials are indicated versus ferrocene/ferrocenium redox couple used as an internal reference (given as IUPAC convention).

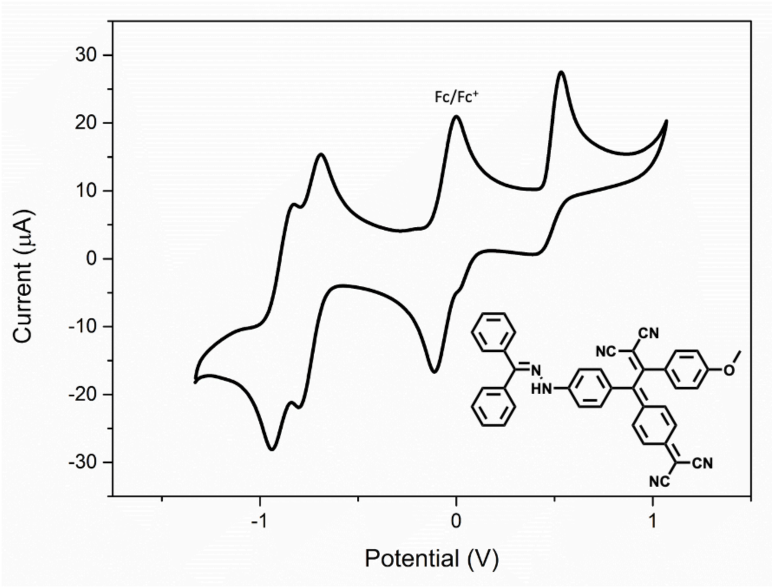

Figure S65. Cyclic voltammogram of **26** at a scan rate of 100 mVs^{-1} in DCM + 0.1 M Bu_4NPF_6. All potentials are indicated versus ferrocene/ferrocenium redox couple used as an internal reference (given as IUPAC convention).

Table S11. Electrochemical gaps by HOMO/LUMO calculations using cyclic voltammetry.

**Compounds**	**ΔE_{redox} (eV)**
**17**	1.15
**18**	1.41
**19**	1.29
**20**	1.50
**21**	–
**23**	0.85
**24**	0.98
**25**	0.92
**26**	0.99
**27**	0.92

6. X-ray Diffraction Analysis

Suitable crystal of **24** was selected for data collection which was performed on a Bruker D8-QUEST diffractometer equipped with a graphite-monochromatic Mo-K_{α} radiation at 293 K. We used these procedures for our analysis: solved by direct methods; SHELXS-2013 [1]; refined by full-matrix least-squares methods; SHELXL-2013 [2]; data collection: Bruker APEX2 [3]; molecular graphics: MERCURY [4]; solution: WinGX [5]. Details of data collection and crystal structure determinations are given in Table S12.

Table S12 Crystal data and structure refinement parameters for **24**.

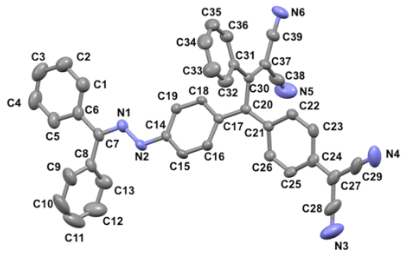

Empirical formula	$C_{39}H_{24}N_6$
Formula weight	576.64
Crystal system	Triclinic
Space group	P-1
a (Å)	11.4659 (9)
b (Å)	11.5841 (9)
c (Å)	14.5192 (11)
α (º)	111.000 (2)
β (º)	94.478 (3)
γ (º)	107.797 (3)
V (Å^3)	1675.6 (2)
Z	2
D_c (g cm^{-3})	1.143
μ (mm^{-1})	0.07
θ range (º)	2.2-25.5
Measured refls.	62979
Independent refls.	7164
R_{int}	0.060
S	0.89
R1/wR2	0.076/0.171
$\Delta\rho_{max}/\Delta\rho_{min}$ (eÅ^{-3})	0.23/-0.21

7. UV/Vis Studies

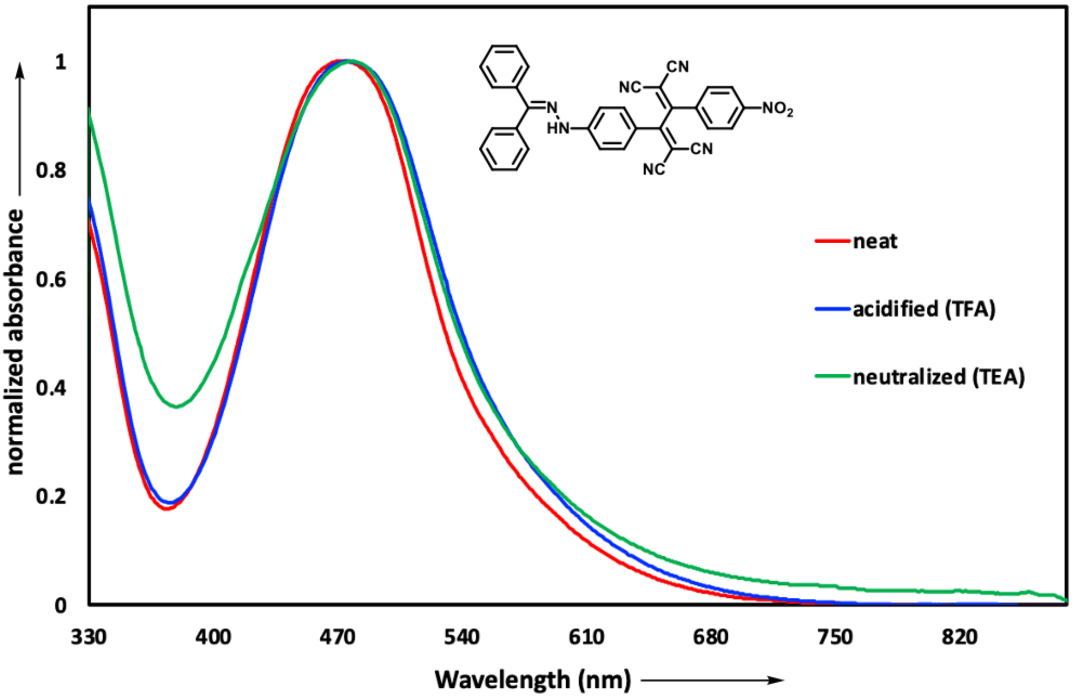

Figure S66. UV/Vis absorption spectra of **19** in CH_2Cl_2 at 298 K recorded neat, after acidification with trifluoroacetic acid (TFA), and after neutralization with triethylamine (Et_3N).

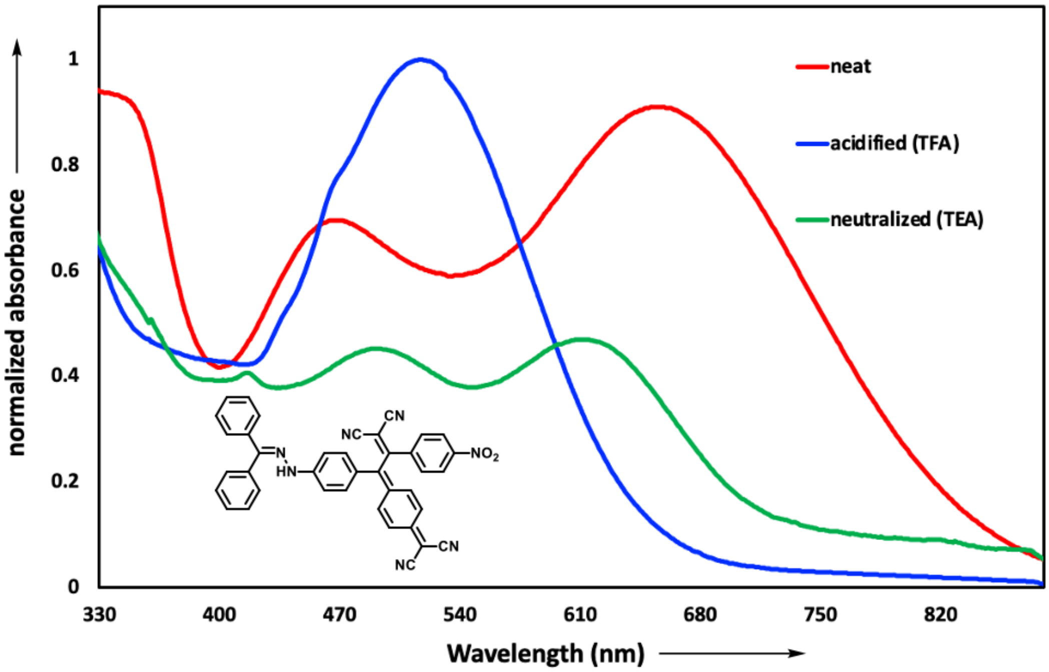

Figure S67. UV/Vis absorption spectra of **25** in CH_2Cl_2 at 298 K recorded neat, after acidification with trifluoroacetic acid (TFA), and after neutralization with triethylamine (Et_3N).

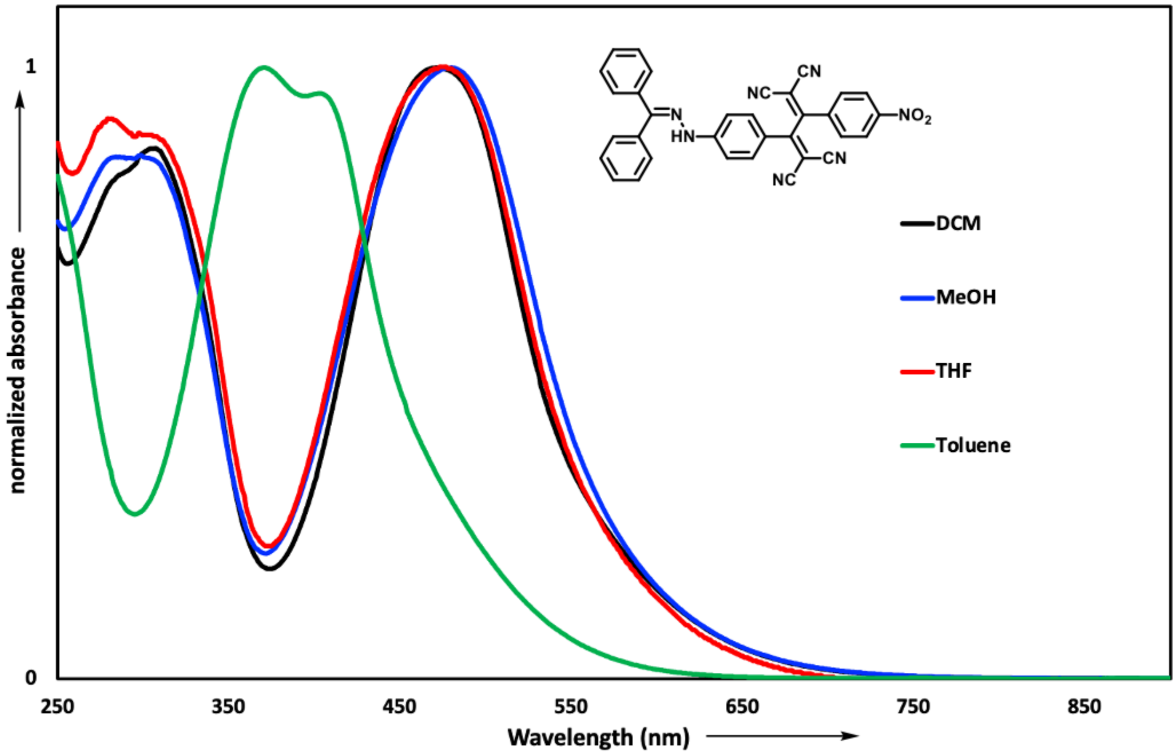

Figure S68. UV/Vis spectra of **19** in different solvents at 298 K.

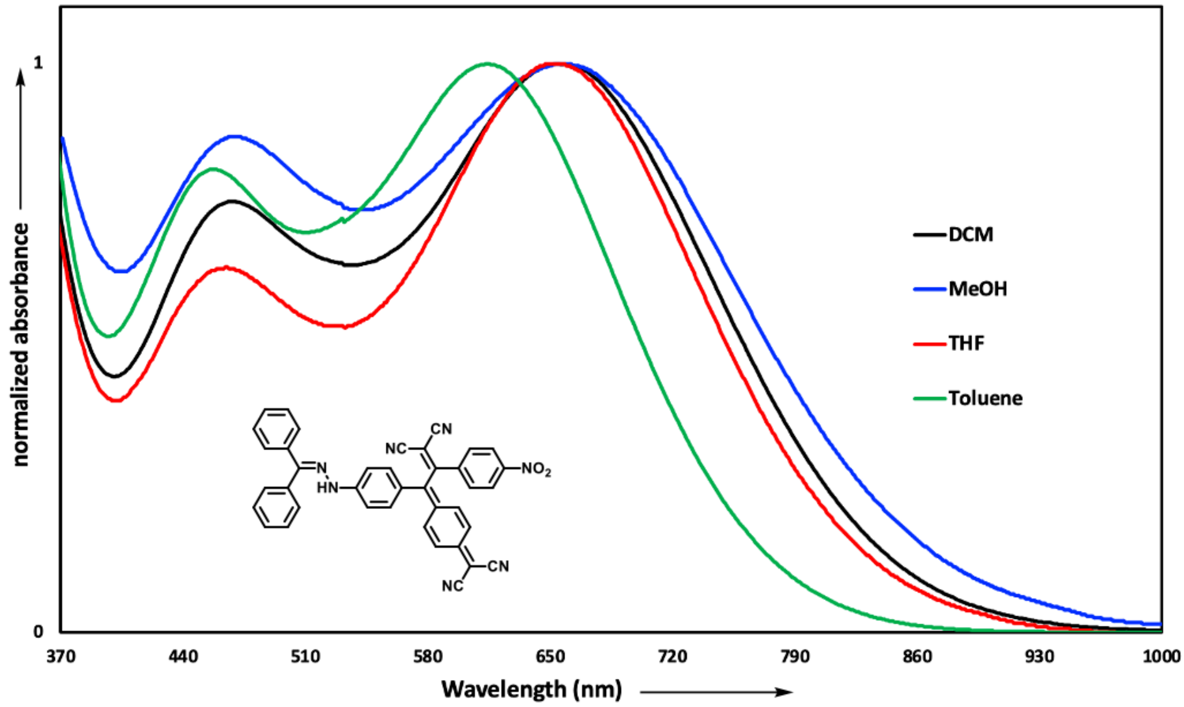

Figure S69. UV/Vis spectra of **25** in different solvents at 298 K.

8. References

[1] Sheldrick, G.M. *Acta Cryst*. **2008**, *A64*, 112.

[2] Sheldrick, G. M. *Acta Cryst*. **2015**, *C71*, 3.

[3] APEX2, Bruker AXS Inc. Madison Wisconsin USA (2013).

[4] Macrae, C. F., Sovago, I., Cottrell, S. J., Galek, P. T. A., McCabe, P., Pidcock, E., Platings, M., Shields, G. P., Stevens, J. S., Towler, M. & Wood, P. A. *J. Appl. Cryst*. **2020**, *53*, 226–235.

[5] Farrugia, L. J. J. Appl. Cryst. **2012**, *45*, 849–854.
